# Tuning Benzylic C−H Functionalization of (Thio)xanthenes with Electrochemistry

**DOI:** 10.3390/molecules28166139

**Published:** 2023-08-19

**Authors:** Changji Wang, Na Yang, Chao Li, Jian He, Hongji Li

**Affiliations:** 1 School of Chemical Engineering, Anhui University of Science and Technology, 168 Taifeng Road, Huainan 232001, China; 2 Key Laboratory of Green and Precise Synthetic Chemistry and Applications, Ministry of Education, School of Chemistry and Materials Science, Huaibei Normal University, Huaibei 235000, China; nayang33@163.com (N.Y.); lichaochem@163.com (C.L.); 3 Hefei New Online Technology Co., Ltd., Hefei 235000, China; hefeixinshangxian@163.com

**Keywords:** electrochemistry, (thio)xanthenes, benzylic C−H functionalization, alkynes, nitriles

## Abstract

Here, we report a tunable electrochemical benzylic C−H functionalization of (thio)xanthenes with terminal alkynes and nitriles in the absence of any catalyst or external chemical oxidant. The benzylic C−H functionalization can be well controlled by varying the electrochemical conditions, affording the specific coupling products via C−C and C−N bond formation.

## 1. Introduction

**Figure 1:**
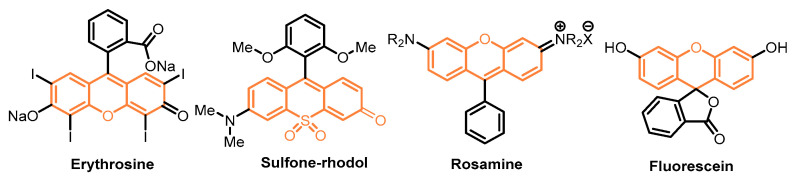
Representative molecules accessible from (thio)xanthenes.

**Scheme 1:**
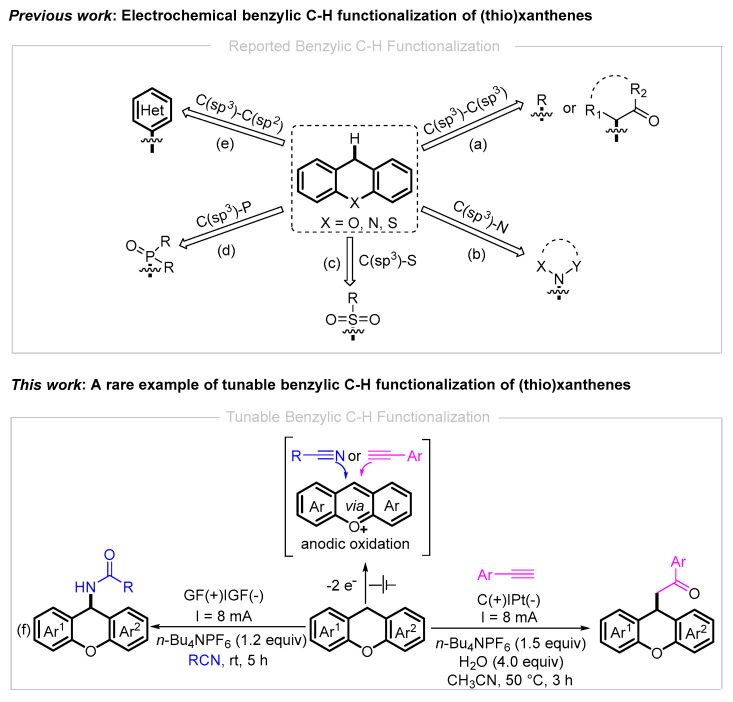
Electrochemical methods for the benzylic C(sp^3^)−H functionalization of (thio)xanthenes.

The C(sp^3^)−H functionalization represents a key challenge in the field of synthetic chemistry; in particular, the chemoselectivity is extremely difficult to be tuned and controlled even when some C(sp^3^)−H bonds are intrinsically reactive, such as the benzylic C(sp^3^)−H bond [1,2,3,4]. Currently, significant progress has been achieved in the direct functionalization of benzylic C(sp^3^)−H bond with strategies of transition metal catalysis, photochemistry and electrochemistry, thus effectively enhancing the efficiency of organic molecules syntheses [5,6,7,8,9,10]. Notably, (thio)xanthenes containing unique benzylic C(sp^3^)−H bonds are synthetic intermediates and also have received increasing attention due to their broad applications in the fields of photodynamic therapy, pharmaceuticals and fluorescent materials, among others (Figure 1) [11,12,13,14,15]. Accordingly, a number of methods have been established for the direct benzylic C(sp^3^)−H functionalization of (thio)xanthenes [16,17,18,19,20,21,22,23,24,25,26,27,28,29]. However, the success of previously reported methods heavily relied on the use of toxic Pd and Cu metal salts or stoichiometric amounts of chemical oxidants. The renaissance of electrochemical synthesis successfully avoids the above limitation that exists in the reported methods [30,31]. Hitherto, we [32,33,34,35] and some others [36,37,38,39,40] have reported several cross-coupling reactions of C−C, C−N, C−P and C−S with electrochemical methods that can enable the benzylic C(sp^3^)−H functionalization of (thio)xanthenes under given conditions (Scheme 1a–e) [32,33,34,35,36,37,38,39,40].

In addition, it is well known that tuning chemoselectivity involving unsaturated alkenes and alkynes is always a big challenge in the field of organic synthesis [41,42,43]. For alkyne addition, achieving high regioselectivity is very challenging, mainly due to critical conditions such as high temperature and transition metals. Additionally, most of the reported reactions involving nitrile often proceed in a sequence of distinct steps upon treatment with high temperatures, high pressure or strong inorganic acid, and overhydrolysis is not absolutely controlled. Although much advancement in benzylic C−H functionalization with electrochemistry has been achieved, investigation with an electrochemical method for tuning the electrochemical benzylic C−H functionalization of (thio)xanthenes with terminal alkynes and nitriles has been less explored. Herein, we will describe a rare approach for the direct benzylic C(sp^3^)−H functionalization of (thio)xanthenes that can be well tuned by varying the reaction temperature and reaction medium, leading to the formation of carbon−carbon and carbon−nitrogen bonds in an efficient manner, respectively (Scheme 1f).

## 2. Results and Discussion

Initially, we chose xanthene **1a** and phenylacetylene **2a** as coupling partners for the optimization of reaction conditions, and the results are listed in Table 1. Based on our recent findings regarding electrochemical benzylic C−H functionalization [32,33,34,35], acetonitrile was preferentially selected as a reaction medium for this investigation. With ^n^Bu_4_NPF_6_ as an electrolyte, the reaction of **1a** with **2a** was performed under constant-current conditions (8 mA) in an undivided cell equipped with a carbon anode and a platinum cathode. After proceeding at room temperature under an air atmosphere for 3 h, the product **3a** was isolated in 32% yield. Meanwhile, the formation of **4a** generated from the reaction of **1a** with CH_3_CN was obtained with a 40% yield (entry 1). The ratio of **3a**/**4a** was effectively improved by varying the reaction temperature from 30 to 60 °C (entries 2–4). Remarkably, reaction temperature could control the formation of **3a** as a major product, but higher temperature (60–70 °C) led to an inferior yield of **3a** (entries 5 and 6). Furthermore, the use of C(+)|Ni(−) or Pt(+)|C(−) still resulted in the mixture of **3a**/**4a** (entries 7 and 8). Additionally, the amount of water extremely affects the formation of **3a** (for details, see Table S1 in Supporting Information). Interestingly, we then found that the model reaction with GF(+)|GF(−) as electrode generated **4a** as a sole product, albeit with 43% isolated yield (entry 9). Screening of both electrolytes and constant current failed to enhance the yield of **3a** (entries 10–13). We clearly observed that the reaction temperature and electrode could affect the formation of **3a** and **4a** (entries 4, 7–9). Subsequently, with GF(+)|GF(−) as an electrode, **4a** was formed at rt to give a 35% yield (entry 14). The use of dry CH_3_CN could enhance the yield of **4a** (entries 15–16). Replacing *n*-Bu_4_NBF_4_ with *n*-Bu_4_NPF_6_ did not improve the benzylic C−H amination of xanthene electrolytes (entry 17). Our attempt to improve the yield of **4a** by varying the constant current from 5 to 10 mA was unsuccessful (entries 18–19). The above model reaction did not proceed without an electric current (entry 20). Notably, Faraday efficiency values for **3a** and **4a** were determined as 45.6% and 28.5%, respectively (for details, see the Supporting Information).

**Scheme 2:**
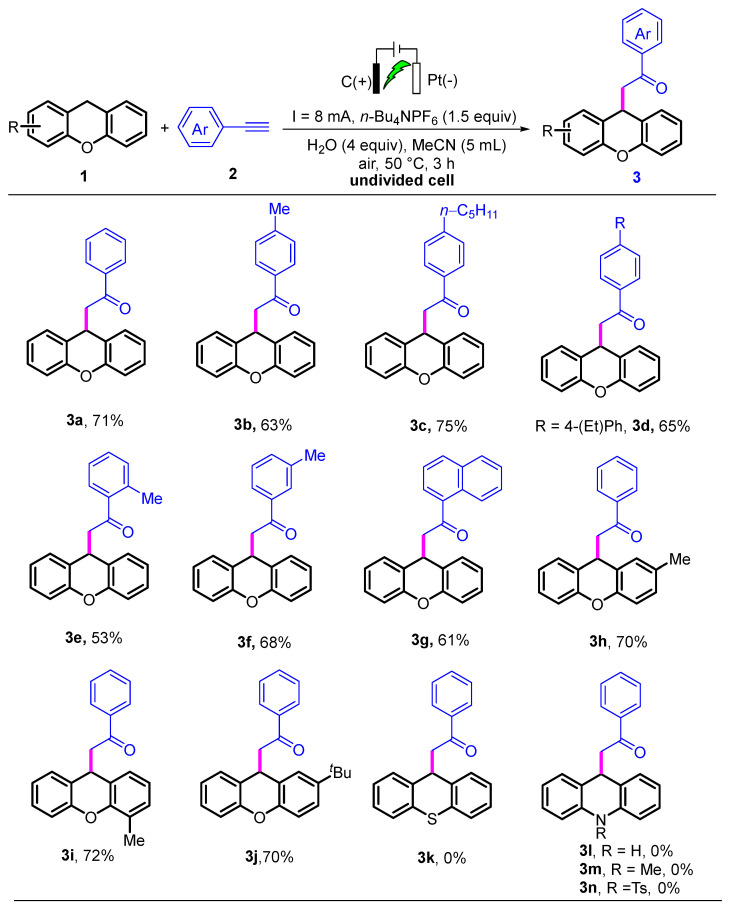
Cross-coupling reaction of xanthenes with terminal alkynes *^a,b^*. *^a^* Reaction conditions: **1** (0.45 mmol), **2** (0.3 mmol), *n*-Bu_4_NPF_6_ (1.2 equiv), H_2_O (4 equiv), CH_3_CN (5.0 mL), carbon rod (Φ 6 mm) anode, Pt plate (1 cm × 1 cm) cathode, constant current = 8 mA, 50 °C, 3 h (Q = 2.98 F mol^−1^). *^b^* Isolated yields are shown.

With the optimized reaction conditions in hand, we next focused on the scope of this tunable benzylic C−H functionalization of xanthenes (Scheme 2). We first examined the electrochemical reaction of the aromatic terminal alkynes with xanthenes under the optimized conditions described in entry 4 of Table 1. Several typical terminal alkynes were used to react with xanthene **1a** under standard conditions and produced the cross-coupling products in good yields. On the terminal alkyne substrates listed in Scheme 2, the ones having electron-rich groups, such as Me, *n*-amyl and 4-Et-Phenyl, incorporated at *para*-position reacted with **1a** to generate the asymmetric ɑ-alkylated aryl ketones **3b**-**d** in 63–75% isolated yields. However, the use of a methyl group at the *ortho*-, *meta*-position in alkynes led to decreasing yields of the target product **3e** and **3f** at 53% and 68%, respectively. The results indicate that there might be a detrimental steric effect in this electrochemical benzylic C−H functionalization. We found that this reaction was also applicable to 1-ethynylnaphthalene and produced **3g** in 61% yield. Several typical substituted xanthenes were then examined under the above system, showing that yield of the product was insensitive to the position of the alkyl group on the aromatic ring (**3h**–**j**). Unfortunately, it was found that 9*H*-thioxanthene and 9,10-dihydroacridines did not react with **2a** under the standard reaction conditions.

**Scheme 3:**
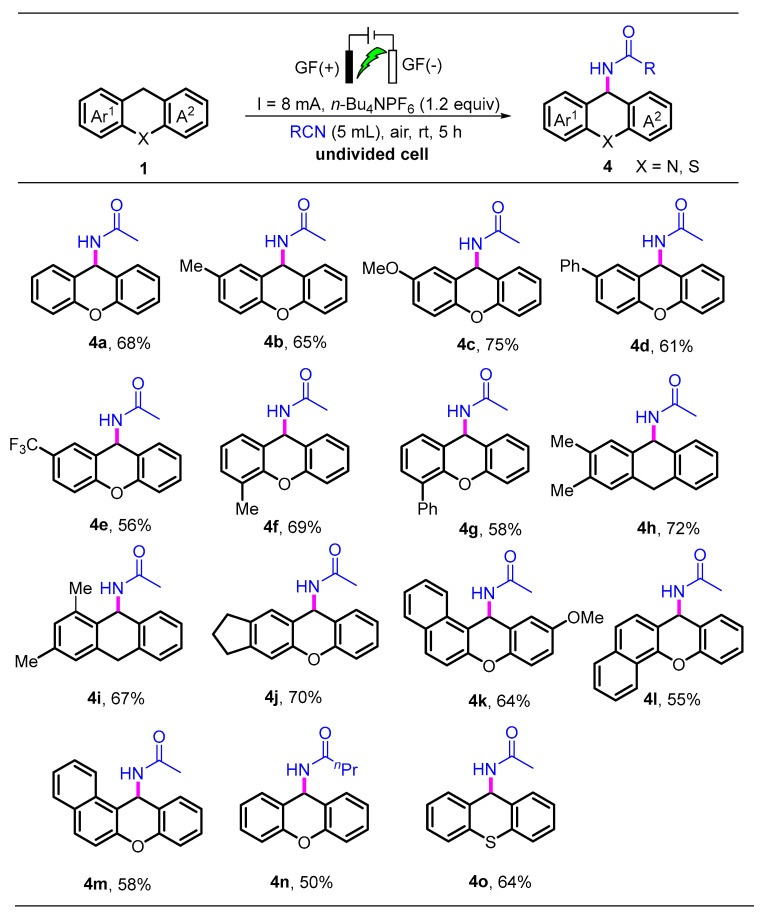
Direct benzylic C−H amination of (thio)xanthenes with nitriles ^*a,b*^. *^a^* Reaction conditions: **1a** (0.3 mmol), *n*-Bu_4_NPF_6_ (1.2 equiv), CH_3_CN (5.0 mL), graphite felt (1 cm × 1 cm) anode, graphite felt (1 cm × 1 cm) cathode, constant current = 8 mA, air, rt, 5 h. (Q = 4.97 F mol^−1^). *^b^* Isolated yields are shown.

We next explored the Ritter-type amination of xanthenes with nitriles under the optimal electrochemical conditions described in Table 1, and the results are listed in Scheme 3. With acetonitrile as a solvent and substrate, a variety of 2-substituted xanthenes including Me, Et, Ph and CF_3_ were all viable substrates and afforded amination products in 56–75% yields (**4b**–**e**). This result indicates that the introduction of an electron-withdrawing group does not favor this electrochemical process. Then, we examined the xanthene derivatives having Me or Ph substituents at the 4-position and found that the reaction proceeded smoothly to form the corresponding products in acceptable yields (**4f** and **4g**). Next, the reactions with dimethylsubstituted xanthenes generated the corresponding products in 67–72% yields (**4h**–**j**). Similarly, the reaction with asymmetric xanthenes having a naphthyl group also proceeded smoothly, affording the amination products in moderate yields (**4k**–**m**). Additionally, the common solvent *n*-butyronitrile was employed as a substrate to react with **1a** under optimal conditions, generating the compound **4n** in 50% yield. Finally, in the case of thioxanthene, the desired product **4o** was obtained in 64% yield.

**Figure 2:**
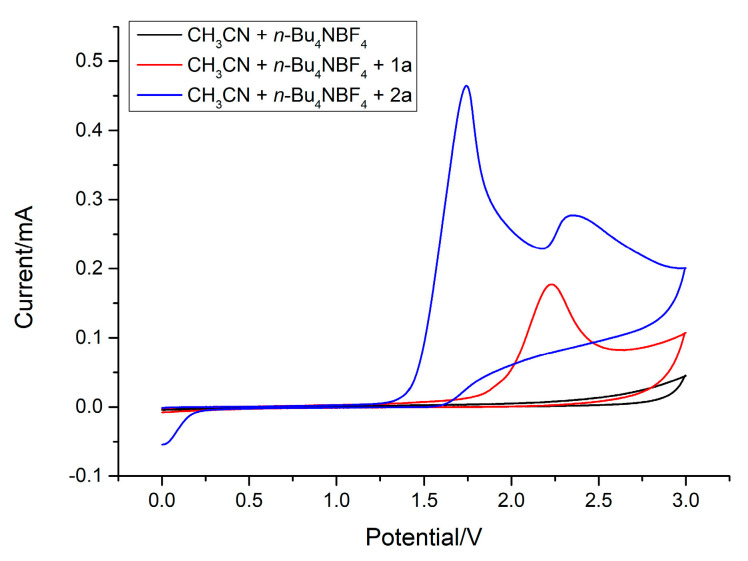
The cyclic voltammograms recorded in CH_3_CN with 0.1 M *n*-Bu_4_NBF_4_ as a supporting electrolyte (**1a** (1 mM), **2a** (1 mM)).

**Scheme 4:**
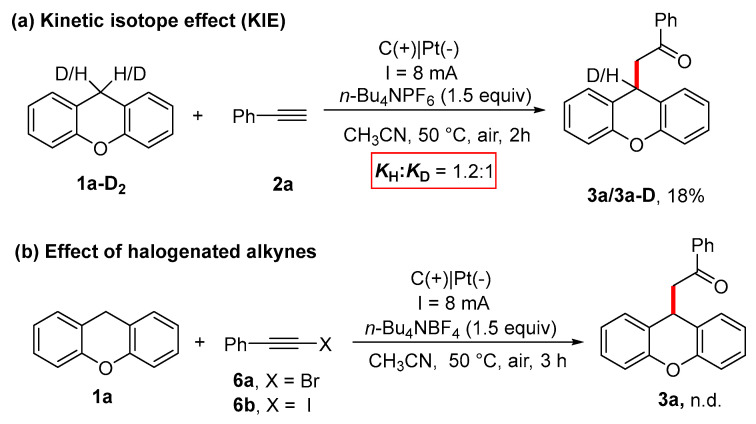
Mechanistic studies and control experiments.

To gain insight into the reaction mechanism, some experiments were specially conducted under given conditions. We observed that the oxidation potential of xanthene **2a** (first peak at Ep 1.74 V) is lower than that of **1a** (Ep 2.23 V) with the method of cyclic voltammograms (CVs), as shown in Figure 2, indicating that preferential oxidation of **2a** occurred and enabled the following electrophilic addition to alkyne (Scheme 4) [35,44]. Then, an experiment involving the mixture of **1a**/**1a-D_2_** and **2a** revealed a KIE value showing that cleavage of benzylic C(sp^3^)−H of xanthene may not be involved in the rate-determining step (Scheme 4a). Importantly, replacing **2a** with halogenated alkynes **6a**–**c** as substrates did not yield any product, thus showing the critical role of terminal alkynes (Scheme 4b).

**Scheme 5:**
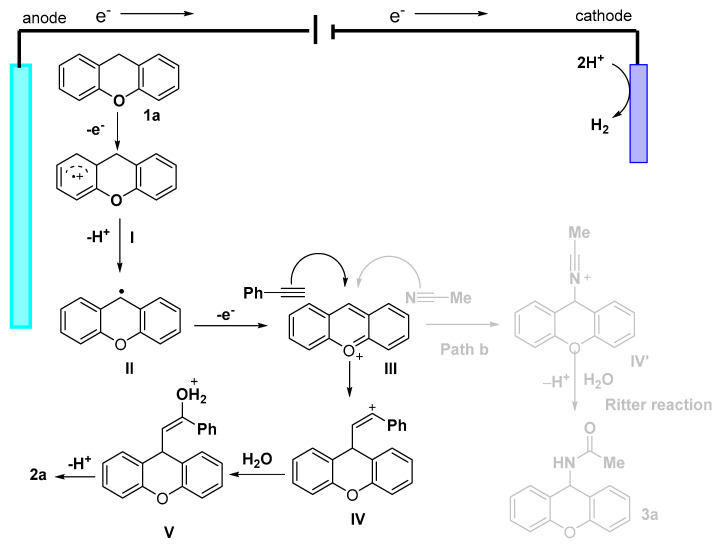
Possible reaction mechanism.

According to the experiment results and previous reports, Refs. [32,33,34,35,36,37,38,39,40], a possible mechanism for the direct electrochemical reaction of xanthenes with terminal alkynes is proposed (Scheme 5). Initially, xanthene **1a** was oxidized into **I** followed by losing a proton to yield **II**. Then, intermediate **II** underwent anodic oxidation to produce a key cationic intermediate **III**. On one hand, **III** could be attacked by terminal alkyne **1a** to generate **IV**, and **IV** would be trapped by H_2_O to give the **V**. Subsequently, V underwent deprotonation and isomerization to afford **2a** (Path a) [35,44]. On the other hand, intermediate **III** would be trapped by an acetonitrile molecule (Ritter-type reaction) to generate **3a** (Path b) [45].

## 3. Materials and Methods

### 3.1. General Considerations

NMR spectra were recorded on a Bruker-600 (Bruker, Germany (600 MHz for ^1^H; 151 MHz for ^13^C). ^1^H NMR spectra were referenced relative to internal Si(Me)_4_ (TMS) at δ 0.00 ppm or CDCl_3_ at δ 7.26 ppm. ^13^C NMR spectra were recorded at ambient temperature on Bruker-600 (151 MHz) spectrometers and are referenced relative to CDCl_3_ at δ 77.16 ppm. Data for ^1^H, ^13^C NMR are recorded as follows: chemical shift (δ, ppm), multiplicity (s = singlet, d = doublet, t = triplet, m = multiplet, q = quartet, quint = quintet, br = broad), integration and coupling constant (Hz). High-resolution mass spectra were recorded on a P-SIMS-Gly produced by Bruker Daltonics Inc. (Bruker, Germany) using electrospray-ionization time of flight (ESI-TOF) and an Agilent Technologies 7250 GCQTOF using EI-TOF (Agilent Technologies, CA, USA). *n*-Bu_4_NBF_4_, phenylacetylene and CH_3_CN were purchased from Energy Chemical Company and Taitan Chemical Company in China. Other substituted xanthenes and thioxanthenes were synthesized according to the known methods [32,34].

### 3.2. Typical Procedure for the Synthesis of ***3a***

To an undivided cell (10 mL columnar round-bottom flask with a 24# mouth) fitted with a carbon rod (Φ 6 mm) anode and a platinum cathode (10 mm × 10 mm × 0.3 mm), the solid reagents xanthene (0.45 mmol) and *n*-Bu_4_NPF_6_ (0.36 mmol) were added. Then, the liquid reagents phenylacetylene (0.3 mmol), H_2_O (1.2 mmol) and CH_3_CN (5 mL) were added in sequence via syringe. Electrolysis was carried out with a constant current (5 mA) at 50 °C for 5 h. Then, the solvent was evaporated to dryness under reduced pressure, and the residue was purified by column chromatography on silica gel to give product **3a** as a white solid (63.8 mg, 71% yield).

*1-Phenyl-2-(9H-xanthen-9-yl)ethan-1-one* (**3a**) was prepared following general procedure [46], and the reaction mixture was purified by flash column chromatography with petroleum ether and ethylacetate (PE/EA = 100:1) to afford the product **3a** (63.8 mg, 71% yield). ^1^H NMR (600 MHz, CDCl_3_) δ 7.81 (d, *J* = 7.2 Hz, 2H), 7.50 (t, *J* = 7.2 Hz, 1H), 7.37 (t, *J* = 7.8 Hz, 2H), 7.32 (dd, *J* = 7.2, 1.2 Hz, 2H), 7.23–7.19 (m, 2H), 7.12 (d, *J* = 8.4 Hz, 2H), 7.04–7.00 (m, 2H), 4.85 (t, *J* = 6.0 Hz, 1H), 3.35 (d, *J* = 6.6 Hz, 2H). ^13^C NMR (151 MHz, CDCl_3_) δ 197.9, 152.4, 137.0, 133.1, 128.8, 128.5, 128.1, 127.9, 125.5, 123.5, 116.6, 49.7, 34.7. HRMS (ESI) calcd. for C_21_H_16_NaO_2_^+^ ([M + Na]^+^): 323.1043, found: 323.1044.
*1-(p-tolyl)-2-(9H-Xanthen-9-yl)ethan-1-one* (**3b**) was prepared following general procedure, [47] and the reaction mixture was purified by flash column chromatography with petroleum ether and ethylacetate (PE/EA = 100:1) to afford the product **3b** (59.2 mg, 63% yield). White solid; m.p.: 108~109 °C. ^1^H NMR (600 MHz, CDCl_3_) δ 7.71 (d, *J* = 7.8 Hz, 2H), 7.32 (dd, *J* = 7.8, 1.2 Hz, 2H), 7.22–7.19 (m, 2H), 7.17 (d, *J* = 8.4 Hz, 2H), 7.11 (dd, *J* = 7.8, 1.2 Hz, 2H), 7.03–7.00 (m, 2H), 4.85 (t, *J* = 6.6 Hz, 1H), 3.32 (d, *J* = 6.6 Hz, 2H), 2.36 (s, 3H). ^13^C NMR (151 MHz, CDCl_3_) δ 197.69, 152.46, 144.09, 134.68, 129.33, 128.98, 128.36, 127.95, 125.75, 123.59, 116.65, 49.77, 34.81, 21.73. HRMS (ESI) calcd. for C_22_H_18_NaO_2_^+^ ([M + Na]^+^): 337.1199, found: 337.1203.
*1-(4-Pentylphenyl)-2-(9H-xanthen-9-yl)ethan-1-one* (**3c**) was prepared following general procedure, and the reaction mixture was purified by flash column chromatography with petroleum ether and ethylacetate (PE/EA = 100:1) to afford the product 3c (82.2 mg, 75% yield). White solid; m.p.: 117~118 °C. ^1^H NMR (600 MHz, CDCl_3_) δ 7.73 (d, *J* = 8.4 Hz, 2H), 7.32 (dd, *J* = 7.8, 1.2 Hz, 2H), 7.22–7.16 (m, 2H), 7.17 (d, *J* = 8.4 Hz, 2H), 7.11 (d, *J* = 8.4 Hz, 2H), 7.03–7.00 (m, 2H), 4.85 (t, *J* = 6.6 Hz, 1H), 3.33 (d, *J* = 6.6 Hz, 2H), 2.60 (t, *J* = 7.8 Hz, 2H), 1.58–1.32 (m, 2H), 1.31–1.25 (m, 4H), 0.88 (t, *J* = 7.2 Hz, 3H). ^13^C NMR (151 MHz, CDCl_3_) δ 197.5, 152.3, 148.9, 134.7, 128.9, 128.6, 128.3, 127.8, 125.7, 123.5, 116.5, 49.7, 34.6, 31.4, 30.8, 22.5, 22.3, 14.0. HRMS (ESI) calcd. for C_26_H_26_NaO_2_^+^ ([M + Na]^+^): 393.1825, found: 393.1826.
*1-(4′-Ethyl-[1,1′-biphenyl]-4-yl)-2-(9H-xanthen-9-yl)ethan-1-one* (**3d**) was prepared following general procedure, and the reaction mixture was purified by flash column chromatography with petroleum ether and ethylacetate (PE/EA = 100:1) to afford the product **3d** (78.6 mg, 65% yield). White solid; m.p.: 122~123 °C. ^1^H NMR (600 MHz, CDCl_3_) δ 7.86 (d, *J* = 8.4 Hz, 2H), 7.59 (d, *J* = 8.4 Hz, 2H), 7.51 (d, *J* = 7.8 Hz, 2H), 7.34 (dd, *J* = 7.8, 1.8 Hz, 2H), 7.29 (d, *J* = 8.4 Hz, 2H), 7.23–7.20 (m, 2H), 7.13 (dd, *J* = 7.8, 0.6 Hz, 2H), 7.03 (td, *J* = 7.2, 1.2 Hz, 2H), 4.88 (t, *J* = 6.6 Hz, 1H), 3.38 (d, *J* = 6.6 Hz, 2H), 2.70 (q, *J* = 7.8 Hz, 2H), 1.28 (s, 3H). ^13^C NMR (151 MHz, CDCl_3_) δ 197.6, 152.5, 145.9, 144.8, 137.2, 135.6, 129.0, 128.8, 128.6, 128.0, 127.3, 127.0, 125.7, 123.6, 116.7, 49.9, 34.9, 28.7, 15.7. HRMS (ESI) calcd. for C_29_H_24_NaO_2_^+^ ([M + Na]^+^): 427.1669, found: 427.1668.
*1-(o-tolyl)-2-(9H-Xanthen-9-yl)ethan-1-one* (**3e**) was prepared following general procedure, and the reaction mixture was purified by flash column chromatography with petroleum ether and ethylacetate (PE/EA = 100:1) to afford the product **3e** (49.8 mg, 53% yield). White solid; m.p.: 105~106 °C. ^1^H NMR (600 MHz, CDCl_3_) δ 7.33 (d, *J* = 7.2 Hz, 2H), 7.30–7.27 (m, 2H), 7.21 (t, *J* = 7.8 Hz, 2H), 7.18 (d, *J* = 7.2 Hz, 1H), 7.12–7.09 (m, 3H), 7.04 (t, *J* = 7.2 Hz, 2H), 4.84 (t, *J* = 6.6 Hz, 1H), 3.26 (d, *J* = 6.6 Hz, 2H), 2.43 (s, 3H). ^13^C NMR (151 MHz, CDCl_3_) δ 202.1, 152.5, 138.2, 132.0, 131.4, 128.9, 128.6, 128.0, 125.7, 125.7, 123.6, 116.7, 52.7, 35.0, 21.2. HRMS (ESI) calcd. for C_22_H_18_NaO_2_^+^ ([M + Na]^+^): 337.1199, found: 337.1201.
*1-(m-tolyl)-2-(9H-Xanthen-9-yl)ethan-1-one* (**3f**) was prepared following general procedure, and the reaction mixture was purified by flash column chromatography with petroleum ether and ethylacetate (PE/EA = 100:1) to afford the product **3f** (60.0 mg, 68% yield). White solid; m.p.: 111~112 °C. ^1^H NMR (600 MHz, CDCl_3_) δ 7.54–7.50 (m, 2H), 7.24 (t, *J* = 7.2 Hz, 2H), 7.20–7.16 (m, 2H), 7.13 (t, *J* = 7.8 Hz, 2H), 7.04 (d, *J* = 7.8 Hz, 2H), 6.95 (t, *J* = 7.2 Hz, 2H), 4.77 (t, *J* = 6.6 Hz, 1H), 3.26 (d, *J* = 6.6 Hz, 2H), 2.26 (s, 3H). ^13^C NMR (151 MHz, CDCl_3_) 198.3, 152.6, 138. 6, 137.2, 134.1, 129.1, 128.9, 128.6, 128.1, 125.8, 125.5, 123.7, 116.8, 50.0, 34.9, 21.5.
*1-(Naphthalen-1-yl)-2-(9H-xanthen-9-yl)ethan-1-one* (**3g**) was prepared following general procedure, and the reaction mixture was purified by flash column chromatography with petroleum ether and ethylacetate (PE/EA = 100:1) to afford the product **3g** (64.2 mg, 61% yield). White solid; m.p.: 116~117 °C. ^1^H NMR (600 MHz, CDCl_3_) δ 8.50 (d, *J* = 8.4 Hz, 1H), 7.91 (d, *J* = 7.8 Hz, 1H), 7.84 (d, *J* = 7.8 Hz, 1H), 7.59–7.55 (m, 1H), 7.59–7.50 (m, 1H), 7.48 (dd, *J* = 7.2, 1.1 Hz, 1H), 7.38 (dd, *J* = 7.2, 1.2 Hz, 2H), 7.36–7.32 (m, 1H), 7.24–7.21 (m, 2H), 7.12 (dd, *J* = 8.4, 1.2 Hz, 2H), 7.06–7.03 (m, 2H), 4.94 (t, *J* = 6.6 Hz, 1H), 3.42 (d, *J* = 6.6 Hz, 2H). ^13^C NMR (151 MHz, CDCl_3_) δ 202.2, 152.5, 136.1, 134.0, 132.9, 130.2, 128.9, 128.6, 128.1, 127.9, 126.6, 125.8, 125.5, 124.4, 123.7, 53.2, 35.5. HRMS (ESI) calcd. for C_25_H_18_NaO_2_^+^ ([M+Na]^+^): 373.1199, found: 373.1200.
*2-(2-Methyl-9H-xanthen-9-yl)-1-phenylethan-1-one* (**3h**) was prepared following general procedure, and the reaction mixture was purified by flash column chromatography with petroleum ether and ethylacetate (PE/EA = 100:1) to afford the product **3h** (65.8 mg, 70% yield). White solid; m.p.: 102~103 °C. ^1^H NMR (600 MHz, CDCl_3_) δ 7.81 (d, *J* = 7.8 Hz, 2H), 7.50 (t, *J* = 7.2 Hz, 1H), 7.37 (t, *J* = 7.2 Hz, 2H), 7.31 (d, *J* = 7.8 Hz, 1H), 7.19 (t, *J* = 7.2 Hz, 1H), 7.10 (d, *J* = 7.8 Hz, 2H), 7.02–6.89 (m, 3H), 4.80 (t, *J* = 6.6 Hz, 1H), 3.35 (d, *J* = 6.6 Hz, 2H), 2.27 (s, 3H). ^13^C NMR (151 MHz, CDCl_3_) δ 198.2, 152.6, 150.3, 137.2, 133.21, 133.0, 129.2, 129.0, 128.6, 128.6, 128.2, 127.9, 125.7, 125.3, 123.4, 116.6, 116.4, 49.9, 34.8, 20.8.
*2-(4-Methyl-9H-xanthen-9-yl)-1-phenylethan-1-one* (**3i**) was prepared following general procedure, and the reaction mixture was purified by flash column chromatography with petroleum ether and ethylacetate (PE/EA = 100:1) to afford the product **3i** (67.8 mg, 72% yield). White solid; m.p.: 107~108 °C. ^1^H NMR (600 MHz, CDCl_3_) δ 7.71 (d, *J* = 8.4 Hz, 2H), 7.33 (d, *J* = 7.8 Hz, 2H), 7.23 –7.20 (m, 2H), 7.17 (d, *J* = 8.4 Hz, 2H), 7.12 (d, *J* = 8.4 Hz, 2H), 7.02 (t, *J* = 7.8 Hz, 2H), 4.86 (t, *J* = 6.6 Hz, 1H), 3.33 (d, *J* = 6.6 Hz, 2H), 2.36 (s, 3H). ^13^C NMR (151 MHz, CDCl_3_) δ 197.5, 152.3, 143.9, 134.5, 130.0, 129.8, 129.5, 129.5, 129.2, 128.8, 128.3, 128.2, 127.8, 127.3, 125.6, 123.4, 116.5, 49.6, 34.7, 21.6. HRMS (ESI) calcd. for C_22_H_18_NaO_2_^+^ ([M + Na]^+^): 337.1199, found: 337.1196.
*2-(4-(tert-Butyl)-9H-xanthen-9-yl)-1-phenylethan-1-one* (**3j**) was prepared following general procedure, and the reaction mixture was purified by flash column chromatography with petroleum ether and ethylacetate (PE/EA = 100:1) to afford the product **3j** (74.0 mg, 70% yield). White solid; m.p.: 113~114 °C. ^1^H NMR (600 MHz, CDCl_3_) δ 7.78–7.75 (m, 2H), 7.41–7.38 (m, 2H), 7.32 (dd, *J* = 7.8, 1.2 Hz, 2H), 7.22–7.19 (m, 2H), 7.12 (dd, *J* = 8.4, 1.2 Hz, 2H), 7.02 (td, *J* = 7.2, 1.2 Hz, 2H), 4.87 (t, *J* = 6.6 Hz, 1H), 3.34 (d, *J* = 6.6 Hz, 2H), 1.30 (s, 9H). ^13^C NMR (151 MHz, CDCl_3_) δ 196.4, 155.9, 151.3, 133.4, 128.8, 128.7, 127.8, 127.3, 127.0, 126.8, 126.3, 124.7, 124.6, 124.4, 122.4, 121.8, 115.5, 48.7, 34.0, 33.5, 29.9. HRMS (ESI) calcd. for C_25_H_24_NaO_2_^+^ ([M + Na]^+^): 379.1669, found: 379.1670.

### 3.3. Typical Procedure for the Synthesis of ***4a***

To an undivided cell (10 mL columnar round-bottom flask with a 24# mouth) fitted with a graphite felt anode (10 mm × 10 mm × 3 cm) and a graphite felt cathode (10 mm × 10 mm × 3 cm), the solid reagents xanthene (0.3 mmol) and *n*-Bu_4_NPF_6_ (0.36 mmol) were added. Then, the liquid CH_3_CN (5 mL) was added in sequence via syringe. The electrolysis was carried out with a constant current (5 mA) at room temperature for 5 h. Then, the solvent was evaporated to dryness under reduced pressure, and the residue was purified by column chromatography on silica gel to give product **4a** as a white solid (48.7 mg, 68% yield).

*N-(9H-xanthen-9-yl)acetamide* (**4a**) was prepared following general procedure, and the reaction mixture was purified by flash column chromatography with petroleum ether and ethylacetate (PE/EA = 5:1) to afford the product **4a** (48.7 mg, 68% yield). White solid; m.p.: 238~239 °C. ^1^H NMR (600 MHz, CDCl_3_) δ 7.48–7.46 (m, 2H), 7.32–7.28 (m, 2H), 7.13–7.10 (m, 4H), 6.49 (d, *J* = 9.0 Hz, 1H), 6.05 (br, d, *J* = 9.0 Hz, 1H), 2.00 (s, 3H). ^13^C NMR (151 MHz, CDCl_3_) δ 169.2, 151.3, 129.8, 129.4, 123.7, 121.3, 116.8, 44.0, 23.5. HRMS (ESI) calcd. for C_15_H_13_NNaO_2_^+^ ([M + Na]^+^): 262.0838, found: 262.0839.
*N-(2-Methyl-9H-xanthen-9-yl)acetamide* (**4b**) was prepared following general procedure, and the reaction mixture was purified by flash column chromatography with petroleum ether and ethylacetate (PE/EA = 5:1) to afford the product **4b** (49.5 mg, 65% yield). White solid; m.p.: 242~243 °C. ^1^H NMR (600 MHz, CDCl_3_) δ 7.46 (d, *J* = 7.8 Hz, 1H), 7.31–7.27 (m, 1H), 7.11–7.08 (m, 3H), 7.00 (d, *J* = 8.4 Hz, 1H), 6.46 (d, *J* = 9.0 Hz, 1H), 6.00 (br, d, *J* = 9.0 Hz, 1H), 2.32 (s, 3H), 2.01 (s, 3H). ^13^C NMR (151 MHz, CDCl_3_) δ 169.2, 151.4, 149.2, 133.2, 130.2, 129.8, 129.8, 129.3, 123.5, 121.3, 120.8, 116.7, 116.5, 44.1, 23.6, 20.8. HRMS (ESI) calcd. for C_16_H_15_NNaO_3_^+^ ([M + Na]^+^): 276.0095, found: 276.0092.
*N-(2-Methoxy-9H-xanthen-9-yl)acetamide* (**4c**) was prepared following general procedure, and the reaction mixture was purified by flash column chromatography with petroleum ether and ethylacetate (PE/EA = 5:1) to afford the product **4c** (60.3 mg, 75% yield). White solid; m.p.: 251~252 °C. ^1^H NMR (600 MHz, CDCl_3_) δ 7.46 (d, *J* = 7.2 Hz, 1H), 7.31–7.27 (m, 1H), 7.11–7.07 (m, 2H), 7.04 (d, *J* = 9.0 Hz, 1H), 6.98 (d, *J* = 3.0 Hz, 1H), 6.87 (dd, *J* = 9.0, 3.0 Hz, 1H), 6.47 (d, *J* = 9.0 Hz, 1H), 6.01 (br, d, *J* = 9.0 Hz, 1H), 3.79 (s, 3H), 2.01 (s, 3H). ^13^C NMR (151 MHz, CDCl_3_) δ 169.2, 155.7, 151.5, 145.4, 129.8, 129.4, 123.5, 121.7, 120.7, 117.7, 116.7, 116.4, 112.8, 55.9, 44.5, 23.6. HRMS (ESI) calcd. for C_16_H_15_NNaO_3_^+^ ([M + Na]^+^): 292.0944, found 292.0947.
*N-(2-Phenyl-9H-xanthen-9-yl)acetamide* (**4d**) was prepared following general procedure, and the reaction mixture was purified by flash column chromatography with petroleum ether and ethylacetate (PE/EA = 5:1) to afford the product **4d** (57.7 mg, 61% yield). White solid; m.p.: 247~248 °C. ^1^H NMR (600 MHz, CDCl_3_) δ 7.70 (d, *J* = 1.8 Hz, 1H), 7.58–7.56 (m, 2H), 7.54 (dd, *J* = 9.0, 2.4 Hz, 1H), 7.51–7.49 (m, 1H), 7.43 (t, *J* = 7.2 Hz, 2H), 7.36–7.30 (m, 2H), 7.18 (d, *J* = 8.4 Hz, 1H), 7.14–7.11 (m, 2H), 6.57 (d, *J* = 9.6 Hz, 1H), 6.06 (br, *J* = 9.6 Hz, 1H), 2.01 (s, 3H). ^13^C NMR (151 MHz, CDCl_3_) δ 169.2, 151.2, 150.8, 140.2, 136.9, 129.9, 129.5, 129.0, 128.2, 128.2, 127.4, 127.0, 123.8, 121.5, 121.2, 117.2, 116.8, 44.1, 23.6. HRMS (ESI) calcd. for C_21_H_17_NNaO_2_^+^ ([M + Na]^+^): 338.1151, found: 338.1147.
*N-(2-(Trifluoromethyl)-9H-xanthen-9-yl)acetamide* (**4e**) was prepared following general procedure, and the reaction mixture was purified by flash column chromatography with petroleum ether and ethylacetate (PE/EA = 5:1) to afford the product **4e** (51.6 mg, 56% yield). White solid; m.p.: 244~245 °C. ^1^H NMR (600 MHz, CDCl_3_) δ 7.75 (s, 1H), 7.55 (dd, *J* = 8.4, 1.2 Hz, 1H), 7.45 (d, *J* = 8.4 Hz, 1H), 7.35–7.31 (m, 1H), 7.20 (d, *J* = 8.4 Hz, 1H), 7.15 (t, *J* = 7.8 Hz, 1H), 7.13 (d, *J* = 8.4 Hz, 1H), 6.52 (d, *J* = 9.0 Hz, 1H), 6.10 (br, s, 1H), 2.04 (s, 3H). ^13^C NMR (151 MHz, CDCl_3_) δ 169.4, 153.5, 150.7, 129.8, 129.64, 127.4 (q, *J* = 3.6), 126.5 (q, *J* = 3.2), 125.9 (q, *J* = 271.5), 124.5, 123.1, 121.8, 121.3, 120.7, 117.4, 116.9, 43.7, 23.5. ^19^F NMR (565 MHz, CDCl_3_) δ − 77.30. HRMS (ESI) calcd. for C_16_H_12_F_3_NNaO_2_^+^ ([M + Na]^+^): 330.0172, found: 330.0175.
*N-(4-Methyl-9H-xanthen-9-yl)acetamide* (**4f**) was prepared following general procedure, and the reaction mixture was purified by flash column chromatography with petroleum ether and ethylacetate (PE/EA = 5:1) to afford the product **4f** (52.5 mg, 69% yield). White solid; m.p.: 239~240 °C. ^1^H NMR (600 MHz, CDCl_3_) 7.50 (d, *J* = 7.2 Hz, 1H), 7.31 (t, *J* = 8.4 Hz, 2H), 7.16 (d, *J* = 8.4 Hz, 2H), 7.13–7.10 (m, 1H), 7.02 (t, *J* = 7.8 Hz, 1H), 6.50 (d, *J* = 9.0 Hz, 1H), 5.92 (br, d, *J* = 8.4 Hz, 1H), 2.40 (s, 3H), 2.00 (s, 3H). ^13^C NMR (151 MHz, CDCl_3_) δ 169.0, 151.2, 151.0, 139.6, 129.7, 129.4, 129.2, 124.6, 123.5, 121.3, 118.1, 116.9, 116.6, 43.8, 23.4, 21.2. HRMS (ESI) calcd. for C_16_H_15_NNaO_2_^+^ ([M + Na]^+^): 276.0995, found: 276.0997.
*N-(4-Phenyl-9H-xanthen-9-yl)acetamide* (**4g**) was prepared following general procedure, and the reaction mixture was purified by flash column chromatography with petroleum ether and ethylacetate (PE/EA = 5:1) to afford the product 4g (54.7 mg, 58% yield). White solid; m.p.: 251~152 °C. ^1^H NMR (600 MHz, CDCl_3_) δ 7.60 (d, *J* = 7.0 Hz, 2H), 7.51–7.46 (m, 4H), 7.42–7.38 (m, 1H), 7.36 (dd, *J* = 7.8, 1.6 Hz, 1H), 7.29–7.26 (m, 1H), 7.19 (t, *J* = 7.8 Hz, 1H), 7.12 (td, *J* = 7.8, 1.2 Hz, 1H), 7.03 (d, *J* = 8.4 Hz, 1H), 6.56 (d, *J* = 9.0 Hz, 1H), 6.06 (br, *J* = 8.4 Hz, 1H), 2.02 (s, 3H). ^13^C NMR (151 MHz, CDCl_3_) δ 169.2, 151.3, 148.3, 137.5, 130.8, 130.2, 129.8, 129.6, 129.3, 129.1, 128.3, 127.5, 123.9, 123.7, 122.0, 121.3, 116.9, 44.5, 23.6. HRMS (ESI) calcd. for C_21_H_17_NNaO_2_^+^ ([M + Na]^+^): 338.1151, found: 338.1151.
*N-(2,3-Dimethyl-9,10-dihydroanthracen-9-yl)acetamide* (**4h**) was prepared following general procedure, and the reaction mixture was purified by flash column chromatography with petroleum ether and ethylacetate (PE/EA = 5:1) to afford the product **4h** (57.2 mg, 72% yield). White solid; m.p.: 247~248 °C. ^1^H NMR (600 MHz, CDCl_3_) δ 7.66 (dd, *J* = 7.8, 0.6 Hz, 1H), 7.30–7.27 (m, 1H), 7.12 (d, *J* = 8.4 Hz, 1H), 7.10–7.07 (m, 2H), 6.92 (d, *J* = 8.4 Hz, 1H), 6.55 (d, *J* = 9.0 Hz, 1H), 5.85 (br, d, *J* = 7.8 Hz, 1H), 2.27 (s, 3H), 2.26 (s, 3H), 1.91 (s, 3H). ^13^C NMR (151 MHz, CDCl_3_) δ 168.4, 151.1, 150.8, 136.8, 131.9, 130.7, 130.2, 129.2, 123.6, 122.8, 119.0, 116.3, 114.1, 42.9, 23.3, 20.2, 15.2. HRMS (ESI) calcd. for C_17_H_17_NNaO_2_^+^ ([M + Na]^+^): 290.1151, found: 290.1152.
*N-(1,3-Dimethyl-9,10-dihydroanthracen-9-yl)acetamide* (**4i**) was prepared following general procedure, and the reaction mixture was purified by flash column chromatography with petroleum ether and ethylacetate (PE/EA = 5:1) to afford the product **4i** (51.6 mg, 67% yield). White solid; m.p.: 244~245 °C. ^1^H NMR (600 MHz, CDCl_3_) δ 7.66–7.63 (m, 1H), 7.29 (td, *J* = 7.8, 1.8 Hz, 1H), 7.11–7.08 (m, 2H), 6.82 (d, *J* = 4.8 Hz, 2H), 6.47 (d, *J* = 9.6 Hz, 1H), 5.78 (br, d, *J* = 9.0 Hz, 1H), 2.34 (s, 3H), 2.32 (s, 3H), 1.91 (s, 3H). ^13^C NMR (151 MHz, CDCl_3_) δ 168.4, 152.4, 151.3, 139.4, 138.6, 130.2, 129.2, 126.4, 123.7, 122.9, 116.5, 116.2, 115.1, 42.4, 23.4, 21.2, 18.7. HRMS (ESI) calcd. for C_17_H_17_NNaO_2_^+^ ([M + Na]^+^): 290.1151, found: 290.1155.
*N-(1,2,3,10-Tetrahydrocyclopenta[b]xanthen-10-yl)acetamide* (**4j**) was prepared following general procedure, and the reaction mixture was purified by flash column chromatography with petroleum ether and ethylacetate (PE/EA = 5:1) to afford the product **4j** (51.9 mg, 70% yield). White solid; m.p.: 236~237 °C. ^1^H NMR (600 MHz, CDCl_3_) δ 7.58 (dd, *J* = 8.4, 1.8 Hz, 1H), 7.30–7.27 (m, 1H), 7.18 (d, *J* = 8.4 Hz, 1H), 7.11–7.07 (m, 2H), 6.94 (d, *J* = 8.4 Hz, 1H), 6.48 (d, *J* = 9.6 Hz, 1H), 5.81 (br, d, *J* = 9.6 Hz, 1H), 2.99–2.94 (m, 1H), 2.90 (t, *J* = 8.4 Hz, 2H), 2.87–2.81 (m, 1H), 2.17–2.08 (m, 2H), 1.96 (s, 3H). ^13^C NMR (151 MHz, CDCl_3_) δ 168.5, 151.327, 150.6, 144.8, 139.4, 130.2, 129.3, 125.1, 123.6, 122.2, 116.6, 116.4, 114.9, 42.8, 32.5, 31.4, 25.7, 23.4. HRMS (ESI) calcd. for C_18_H_17_NNaO_2_^+^ ([M + Na]^+^): 302.1151, found 302.1154.
*N-(10-Methoxy-12H-benzo[a]xanthen-12-yl)acetamide* (**4k**) was prepared following general procedure, and the reaction mixture was purified by flash column chromatography with petroleum ether and ethylacetate (PE/EA = 3:1) to afford the product **4k** (61.3 mg, 64% yield). White solid; m.p.: 258~259 °C. ^1^H NMR (600 MHz, CDCl_3_) δ 8.07 (d, *J* = 8.4 Hz, 1H), 7.84 (d, *J* = 6.6 Hz, 1H), 7.82 (d, *J* = 8.4 Hz, 1H), 7.60 (t, *J* = 6.6 Hz, 1H), 7.46 (t, *J* = 7.2 Hz, 1H), 7.30 (d, *J* = 9.0 Hz, 1H), 7.25 (d, *J* = 3.0 Hz, 1H), 7.13 (d, *J* = 9.0 Hz, 1H), 7.08 (d, *J* = 9.4 Hz, 1H), 6.92 (dd, *J* = 8.4, 3.0 Hz, 1H), 5.85 (br, d, *J* = 9.6 Hz, 1H), 3.83 (s, 3H), 1.92 (s, 3H). ^13^C NMR (151 MHz, CDCl_3_) δ 168.7, 156.2, 150.3, 144.8, 131.8, 130. 5, 130.4, 128.7, 127.8, 124.8, 123.0, 122.5, 118.0, 117.6, 116.8, 112.6, 111.5, 56.0, 42.2, 23.4. HRMS (ESI) calcd. for C_20_H_17_NNaO_2_^+^ ([M + Na]^+^): 342.1101, found 342.1102.
*N-(7H-benzo[c]xanthen-7-yl)acetamide* (**4l**) was prepared following general procedure, and the reaction mixture was purified by flash column chromatography with petroleum ether and ethylacetate (PE/EA = 5:1) to afford the product **4l** (47.6 mg, 55% yield). White solid; m.p.: 250~251 °C. ^1^H NMR (600 MHz, CDCl_3_)δ 8.43 (d, *J* = 8.4 Hz, 1H), 7.83 (d, *J* = 7.2 Hz, 1H), 7.60 –7.54 (m, 4H), 7.51–7.48 (m, 1H), 7.38–7.35 (m, 1H), 7.30 (dd, *J* = 8.4, 1.2 Hz, 1H), 7.19–7.16 (m, 1H), 6.66 (d, *J* = 9.0 Hz, 1H), 6.04 (br, d, *J* = 9.0 Hz, 1H), 2.02 (s, 3H). **^13^C** NMR (151 MHz, CDCl_3_) δ 169.1, 151.0, 146.3, 134.0, 129.9, 129.3, 127.6, 127.0, 126.3, 126.3, 124.0, 123.9, 123.3, 121.8, 121.2, 116.8, 114.8, 44.2, 23.5. HRMS (ESI) calcd. for C_19_H_15_
*N-(12H-benzo[a]xanthen-12-yl)acetamide* (**4m**) was prepared following general procedure, and the reaction mixture was purified by flash column chromatography with petroleum ether and ethylacetate (PE/EA = 5:1) to afford the product **4m** (50.3 mg, 58% yield). White solid; m.p.: 249~250 °C. ^1^H NMR (600 MHz, CDCl_3_) δ 8.43 (d, *J* = 8.4 Hz, 1H), 7.83 (d, *J* = 7.8 Hz, 1H), 7.60–7.54 (m, 4H), 7.50 (d, *J* = 8.4 Hz, 1H), 7.37 (t, *J* = 7.8 Hz, 1H), 7.30 (d, *J* = 7.8 Hz, 1H), 7.17 (t, *J* = 7.2 Hz, 1H), 6.67 (d, *J* = 9.6 Hz, 1H), 6.01 (br, *J* = 9.0 Hz, 1H), 2.02 (s, 3H). ^13^C NMR (151 MHz, CDCl_3_) δ 169.1, 151.1, 146.4, 134.1, 130.0, 129.4, 127.8, 127.1, 126.4, 126.4, 124.1, 123.4, 121.9, 121.4, 116.9, 114.9, 44.4, 23.6. HRMS (ESI) calcd. for C_19_H_15_NNaO_2_^+^ ([M + Na]^+^): 312.0095, found 312.0098.
*N-(9H-xanthen-9-yl)butyramide* (**4n**) was prepared following general procedure, and the reaction mixture was purified by flash column chromatography with petroleum ether and ethylacetate (PE/EA = 9:1) to afford the product **4n** (40.1 mg, 50% yield). White solid; m.p.: 262~263 °C. ^1^H NMR (600 MHz, CDCl_3_) δ 7.45 (d, *J* = 7.8 Hz, 2H), 7.30 (t, *J* = 7.2 Hz, 2H), 7.12–7.09 (m, 4H), 6.52 (d, *J* = 9.0 Hz, 1H), 6.05 (br, d, *J* = 8.4 Hz, 1H), 2.17 (t, *J* = 7.8 Hz, 2H), 1.72–1.67 (m, 2H), 0.94 (t, *J* = 7.2 Hz, 3H). ^13^C NMR (151 MHz, CDCl_3_) δ 172.2, 151.2, 129.8, 129.4, 123.7, 121.4, 116.8, 43.8, 38.9, 19.3, 13.9. HRMS (ESI) calcd. for C_18_H_19_NO_2_^+^ ([M + H]^+^): 267.1379, found: 267.1377.
*N-(9H-thioxanthen-9-yl)acetamide* (**4o**) was prepared following general procedure, and the reaction mixture was purified by flash column chromatography with petroleum ether and ethylacetate (PE/EA = 5:1) to afford the product **4o** (48.9 mg, 64% yield). White solid; m.p.: 237~238 °C. ^1^H NMR (600 MHz, CDCl_3_) δ 7.58 (dd, *J* = 6.0, 2.4 Hz, 2H), 7.47–7.45 (m, 2H), 7.28–7.25 (m, 4H), 6.30 (d, *J* = 9.0 Hz, 1H), 6.23 (br, d, *J* = 7.2 Hz, 1H), 1.88 (s, 3H). ^13^C NMR (151 MHz, CDCl_3_) δ 169.0, 134.9, 133.0, 129.6, 128.0, 127.2, 127.2, 53.4, 23.5. HRMS (ESI) calcd. for C_15_H_13_NNaOS^+^ ([M + Na]^+^): 278.0610, found: 278.0613.

## 4. Conclusions

In summary, we have developed an electrochemical benzylic C−H functionalization of (thio)xanthenes with terminal alkynes and nitriles in the absence of any catalyst or external chemical oxidant. The method enables selective benzylic C−H bond functionalization by varying electrochemical conditions, providing an efficient approach to synthesizing xanthene derivatives under mild conditions. Efforts in our laboratory are ongoing to explore other challenging inert C−H functionalizations.

## Figures and Tables

**Table 1:** Optimization of the reaction conditions *^a^*^,^*^b^*.

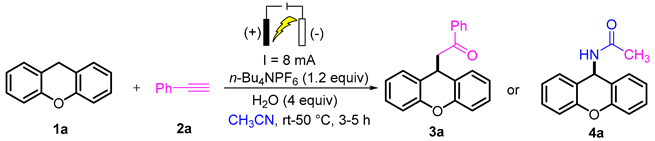				
Entry	Electrode	Temp (°C)	Yield (%)	
			3a	4a
1	C(+)|Pt(−)	rt	32	40
2	C(+)|Pt(−)	30	38	37
3	C(+)|Pt(−)	40	45	21
4	C(+)|Pt(−)	50	71	6
5	C(+)|Pt(−)	60	65	trace
6	C(+)|Pt(−)	70	52	trace
7	C(+)|Ni(−)	50	14	25
8	Pt(+)|C(−)	50	46	17
9	GF(+)|GF(−)	50	n.d.	43
10 *^c^*	C(+)|Pt(−)	50	55	8
11 *^d^*	C(+)|Pt(−)	50	37	10
12 *^e^*	C(+)|Pt(−)	50	48	trace
13 *^f^*	C(+)|Pt(−)	50	22	trace
14	GF(+)|GF(−)	rt	n.d.	35
15 *^f^*	GF(+)|GF(−)	rt	n.d.	50
16 ^*f,g*^	GF(+)|GF(−)	rt	n.d.	77
17 *^c,f,g^*	GF(+)|GF(−)	rt	n.d.	61
18 *^d,f,g^*	GF(+)|GF(−)	rt	n.d.	33
19 *^e,f,g^*	GF(+)|GF(−)	rt	n.d.	58
20 *^h^*	GF(+)|GF(−)	rt	n.d.	n.d.

*^a^* Reaction conditions: **1a** (0.45 mmol), **2a** (0.3 mmol), *n*-Bu_4_NPF_6_ (1.2 equiv.), H_2_O (4.0 equiv.), MeCN (5 mL), constant current = 8 mA, air, 3–5 h. *^b^* Isolated yields. *^c^ n*-Bu_4_NBF_4_ (1.2 equiv.). *^d^* Constant current = 5 mA. *^e^* Constant current = 10 mA. *^f^* Five hours. *^g^* Dry CH_3_CN. *^h^* No electric current. n.d. = Not detected.

## Data Availability

Not applicable.

## Supplementary Materials

The following supporting information can be downloaded at: https://www.mdpi.com/article/10.3390/molecules28166139/s1, Figure S1: Experiment setup for electrochemical benzylic C-H functionalization; Experiment procedure for the CV experiments; ^1^H, ^13^C, ^19^F NMR spectra of the products; Determination of Faradaic efficiency; Figure S2: The cyclic voltammograms recorded in CH_3_CN with 0.1 M *n*-Bu_4_NBF_4_ as the supporting electrolyte [1a (10 mM), 2a (10 mM)]; Table S1: Effect of water on the synthesis of **3a**.

## Sample Availability

Samples of the compounds are available from the authors.
