# Supplementary material for: Tuning Benzylic C−H Functionalization of (Thio)xanthenes with Electrochemistry
Source: Molecules. 2023 Aug 19;28(16):6139. doi: 10.3390/molecules28166139 (PMC10459638; doi:10.3390/molecules28166139)
Supplement: Supplementary file 1 [file molecules-28-06139-s001.zip › molecules-2555395-supplementary.pdf]

Supporting Information

Tuning Benzylic C−H Functionalization of (Thio)xanthenes with Electrochemiatry

Changji Wang,[1] Na Yang,[2] Chao Li,[2] Jian He,[3] and Hongji Li[2,*]

[1] School of Chemical Engineering, Anhui University of Science and Technology, 168 Taifeng Road, Huainan 232001, P. R. China, E-mail address: wangchangji214@126.com.

[2] Key Laboratory of Green and Precise Synthetic Chemistry and Application, Ministry of Education; School of Chemistry and Material Science, Huaibei Normal University, Huaibei, Anhui 235000, P. R. China, E-mail address: hongjili@chnu.edu.cn.

[3] Hefei New Online Technology Co., Ltd, Hefei, Anhui 235000, P. R. China.

Table of Contents

1. General Information ……………………………………………………………S1

2. General Procedure for the Reactions………………….………………………...S1

2.1 Graphical Guide for the Set-up ……………………………….……………S1

2.2 Typical Procedure for the Synthesis of **3a**……………………………………..S2

2.3 Typical Procedure for the Synthesis of **4a**……………………………………..S2

2.4 Effect of Water on the Electrochemical Synthesis of **3a**……………….………S3

3. Mechanistic Experiments……………………………………………………..……S3

3.1 Cyclic Voltammetry Studies …………………………………..….………S3

3.2 Kinetic Isotope Effect Experiment……………..…...………...........................S3

4. Characterization Data for the Products……………….…………………………S5

5. NMR Spectra of the Products………...…...…...…………………………...….S22

6. Determination of Faradaic Efficiency……..…...…...………………….…..…..S47

1. General Information

NMR spectra were recorded on Bruker-600 (Bruker, Germany, 600 MHz for ^{1}H; 151 MHz for ^{13}C). ^{1}H NMR spectra were referenced relative to internal $Si(Me)_4$ (TMS) at δ 0.00 ppm or $CDCl_3$ at δ 7.26 ppm. ^{13}C NMR spectra were recorded at ambient temperature on Bruker-600 (151 MHz) spectrometers and are referenced relative to $CDCl_3$ at δ 77.16 ppm. Data for ^{1}H, ^{13}C NMR are recorded as follows: chemical shift (δ, ppm), multiplicity (s = singlet, d = doublet, t = triplet, m = multiplet, q = quartet, quint = quintet, br = broad), integration, and coupling constant (Hz). High resolution mass spectra were recorded on P-SIMS-Gly of Bruker Daltonics Inc.(Bruker, Germany) using ESI-TOF (electrospray ionization-time of flight) and Aglient Technologies 7250 GCQTOF using EI-TOF (Agilent Technologies, California, America). *n*-Bu_4NBF_4, phenylacetylene and CH_3CN were purchased from Energy Chemical Company and Taitan Chemical Company in China. Some other substituted xanthenes and thioxanthenes were synthesized according to the known methods.

2. General Procedure for the Reactions

2.1 Graphical Guide for the Set-up

As experimental setup, we used a platinum plate anode (10 mm×10 mm×0.3 mm) and a platinum plate cathode (10 mm×10 mm×0.3 mm), rubber stoppers, an undivided 15 mL single-necked flask, a DC adjustable power supply regulator (HY3005MT) (Made in China) and a magnetic stirrer.

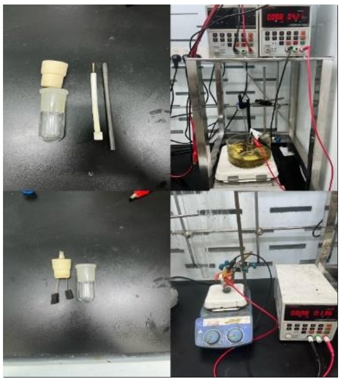

Figure S1 Electrochemical benzylic C−H functionalization of (thio)xanthenes with terminal

alkynes and nitriles

2.2 Typical Procedure for the Synthesis of 3a

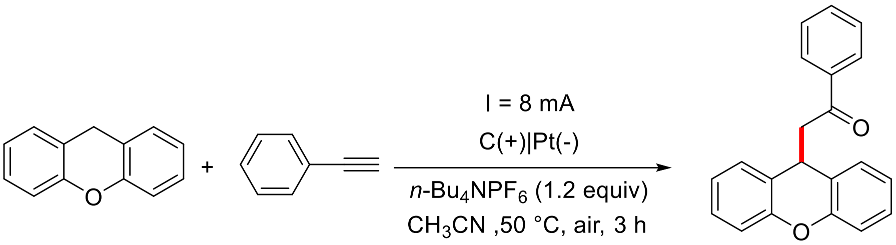

1a **2a** **3a**

To an undivided cell (10 mL columnar round-bottom flask with a 24# mouth) fitted with a carbon rod anode (Φ 6 mm) and a platinum cathode (10 mm×10 mm×0.3 mm), the solid reagents xanthene (0.45 mmol) and *n*-Bu_4NPF_6 (0.36 mmol) were added. Then, the liquid reagents phenylacetylene (0.3 mmol), H_2O (4 equiv) and CH_3CN (5 mL) were added in sequence via syringe. The electrolysis was carried out with constant current (8 mA) at 50 °C for 3 h. Then the solvent was evaporated to dryness under reduced pressure and the residue was purified by column chromatography on silica gel to give product **3a**.

2.3 Typical Procedure for the Synthesis of 4a

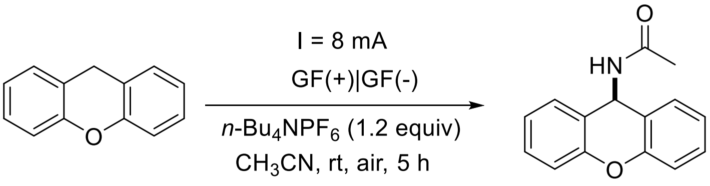

1a **4a**

To an undivided cell (10 mL columnar round-bottom flask with a 24# mouth) fitted with a graphite felt anode (10 mm×10 mm×3 cm) and a graphite felt cathode (10 mm×10 mm×3 cm), the solid reagents xanthene (0.3 mmol) and *n*-Bu_4NPF_6 (0.36 mmol) were added. Then, CH_3CN (5 mL) were added in sequence via a syringe. The electrolysis was carried out with constant current (8 mA) at room temperature for 5 h. Then the solvent was evaporated to dryness under reduced pressure and the residue was purified by column chromatography on silica gel to give product **4a**.

2.4 Effect of Water on the Electrochemical Synthesis of 3a

Table S1. Effect of water on the synthesis of 3a [a,b]

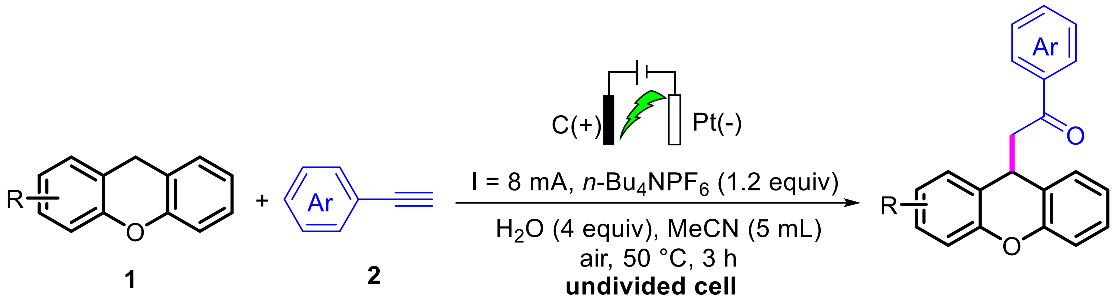

Entry	H_2O (x equiv)	Yield (%)
1	none	trace
2	1	45
3	2	59
4	3	66
5	4	71
6	5	68

[a] Reaction conditions: **1** (0.3 mmol), **2a** (0.2 mmol), *n*-Bu_4NPF_6 (1.2 equiv), dry CH_3CN (5.0 mL), H_2O (x equiv), carbon rod (Φ 6 mm) anode, Pt plate (1 cm × 1 cm) cathode, constant current = 8 mA, 50 °C, air, 3 h. [b] Isolated yields.

3. Mechanistic Experiments

3.1 Cyclic Voltammetry Studies

Cyclic voltammetry was performed in a three electrode cell connected to a Schlenk line at room temperature. The working electrode was a glassy carbon electrode, and the counter electrode was a platinum electrode. The reference was an Ag/AgCl. 10 mL of CH_3CN containing 0.1 M *n*-Bu_4NPF_6 were poured into the electrochemical cell in all experiments. The scan rate is 0.1 V/s, ranging from 0 V to 3.5 V. The test concentrations of **1a** and **2a** are 10 mM.

3.2 Kinetic Isotope Effect Experiment

Xanthene (**1a**, 41.0 mg, 0.225 mmol), [D]-xanthene (**[D]-1a**, 41.5 mg, 0.225 mmol), phenylacetylene (**2a**, 30.6 mg, 0.3 mmol, 1.0 equiv), *n*-Bu_4NBF_4 (118.5 mg, 0.45 mmol, 1.2 equiv), CH_3CN (5 mL) was sequentially added to an undivided cell (10 mL columnar round-bottom flask with a 24# mouth) fitted with a carbon rod anode (Φ

6 mm) and a platinum cathode (10 mm×10 mm×0.3 mm). The reaction mixture was stirred and electrolyzed at a constant current of 8 mA under air at 50 °C for 1 h. After that, the mixture in reaction tube was detected by TLC. The crude product was purified by flash chromatography (silica gel, petroleum ether/ethyl acetate = 20:1), to give the desired product **3a/[D]-3a** in 18% yield.

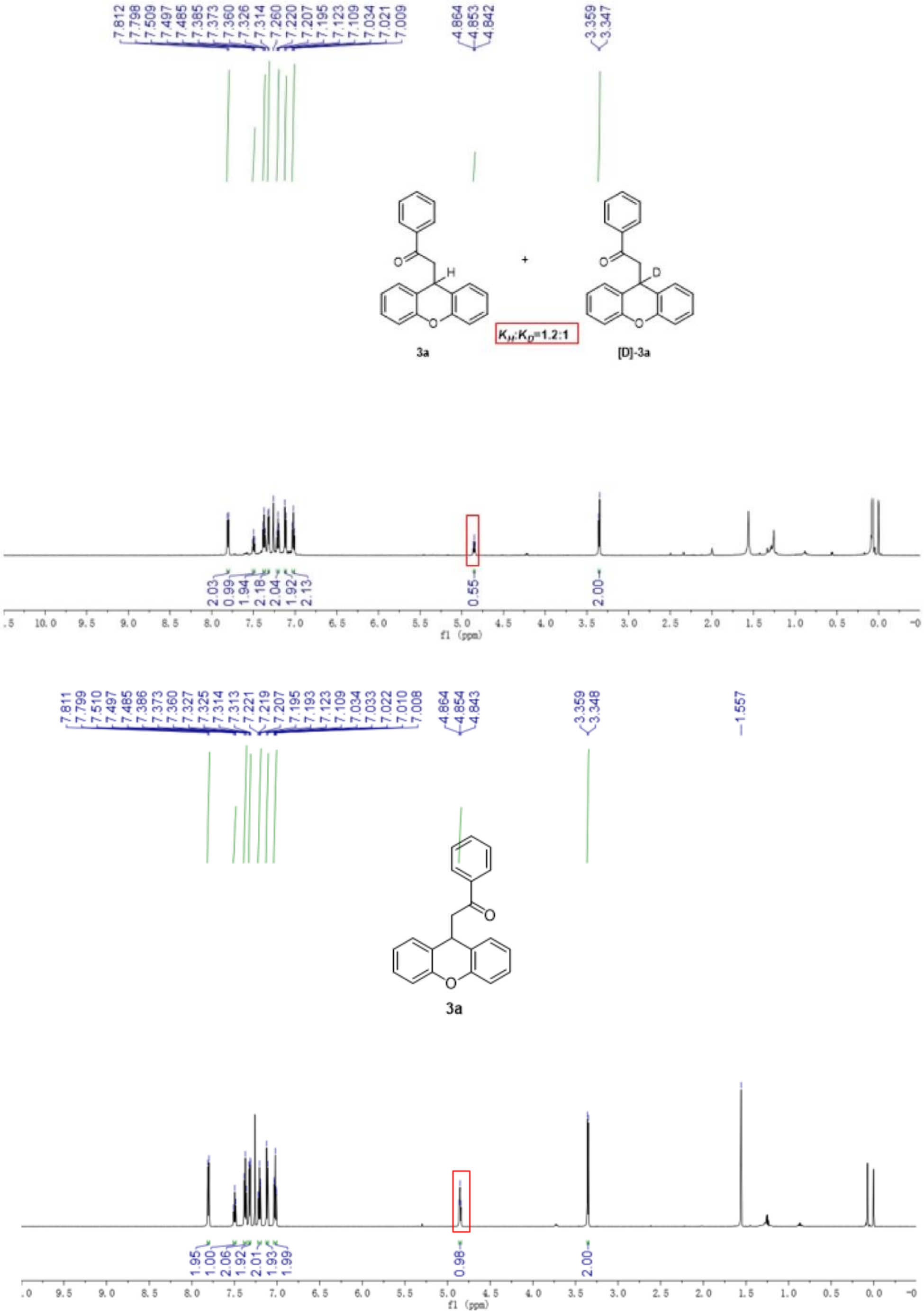

Figure S2 NMR spectra of **3a/[D]-3a**

4. Characterization Data for the Products

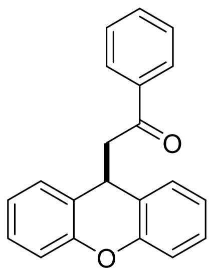

3a

1-Phenyl-2-(9*H*-xanthen-9-yl)ethan-1-one (**3a**)

Prepared following general procedure and the reaction mixture was purified by flash column chromatography with petroleum ether and ethylacetate (PE/EA = 100:1) to afford the product **3a** (63.8 mg, 71% yield).

White solid; m.p.: 105~106 ^{o}C.

^{1}H NMR (600 MHz, $CDCl_3$) δ 7.81 (d, *J* = 7.2 Hz, 2H), 7.50 (t, *J* = 7.2 Hz, 1H), 7.37 (t, *J* = 7.8 Hz, 2H), 7.32 (dd, *J* = 7.2, 1.2 Hz, 2H), 7.23–7.19 (m, 2H), 7.12 (d, *J* = 8.4 Hz, 2H), 7.04–7.00 (m, 2H), 4.85 (t, *J* = 6.0 Hz, 1H), 3.35 (d, *J* = 6.6 Hz, 2H).

^{13}C NMR (151 MHz, $CDCl_3$) δ 197.9, 152.4, 137.0, 133.1, 128.8, 128.5, 128.1, 127.9, 125.5, 123.5, 116.6, 49.7, 34.7.

HRMS (ESI) calcd. for $C_{21}H_{16}NaO_2^+$ ($[M+Na]^+$): 323.1043, found: 323.1044.

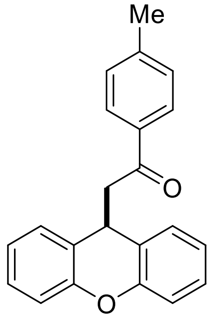

3b

1-(*p*-Tolyl)-2-(9*H*-xanthen-9-yl)ethan-1-one (**3b**)

Prepared following general procedure and the reaction mixture was purified by flash column chromatography with petroleum ether and ethylacetate (PE/EA = 100:1) to afford the product **3b** (59.2 mg, 63% yield).

White solid; m.p.: 108~109 °C.

^{1}H NMR (600 MHz, $CDCl_3$) δ 7.71 (d, *J* = 7.8 Hz, 2H), 7.32 (dd, *J* = 7.8, 1.2 Hz, 2H), 7.22–7.19 (m, 2H), 7.17 (d, *J* = 8.4 Hz, 2H), 7.11 (dd, *J* = 7.8, 1.2 Hz, 2H), 7.03–7.00 (m, 2H), 4.85 (t, *J* = 6.6 Hz, 1H), 3.32 (d, *J* = 6.6 Hz, 2H), 2.36 (s, 3H).

^{13}C NMR (151 MHz, $CDCl_3$) δ 197.69, 152.46, 144.09, 134.68, 129.33, 128.98, 128.36, 127.95, 125.75, 123.59, 116.65, 49.77, 34.81, 21.73.

HRMS (ESI) calcd. for $C_{22}H_{18}NaO_2^+$ ($[M+Na]^+$): 337.1199, found: 337.1203.

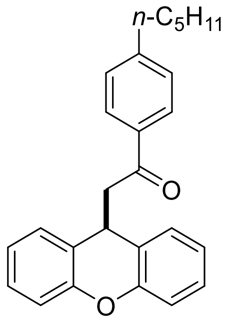

3c

1-(4-Pentylphenyl)-2-(9*H*-xanthen-9-yl)ethan-1-one (3c)

Prepared following general procedure and the reaction mixture was purified by flash column chromatography with petroleum ether and ethylacetate (PE/EA = 100:1) to afford the product **3c** (82.2 mg, 75% yield).

White solid; m.p.: 117~118 °C.

^{1}H NMR (600 MHz, $CDCl_3$) δ 7.73 (d, *J* = 8.4 Hz, 2H), 7.32 (dd, *J* = 7.8, 1.2 Hz, 2H), 7.22–7.16 (m, 2H), 7.17 (d, *J* = 8.4 Hz, 2H), 7.11 (d, *J* = 8.4 Hz, 2H), 7.03–7.00 (m, 2H), 4.85 (t, *J* = 6.6 Hz, 1H), 3.33 (d, *J* = 6.6 Hz, 2H), 2.60 (t, *J* = 7.8 Hz, 2H), 1.58–1.32 (m, 2H), 1.31–1.25 (m, 4H), 0.88 (t, *J* = 7.2 Hz, 3H).

^{13}C NMR (151 MHz, $CDCl_3$) δ 197.5, 152.3, 148.9, 134.7, 128.9, 128.6, 128.3, 127.8, 125.7, 123.5, 116.5, 49.7, 34.6, 31.4, 30.8, 22.5, 22.3, 14.0.

HRMS (ESI) calcd. for $C_{26}H_{26}NaO_2^+$ ([M+Na]$^+$): 393.1825, found: 393.1826.

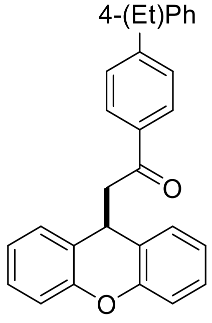

3d

1-(4'-Ethyl-[1,1'-biphenyl]-4-yl)-2-(9*H*-xanthen-9-yl)ethan-1-one (3d)

Prepared following general procedure and the reaction mixture was purified by flash column chromatography with petroleum ether and ethylacetate (PE/EA = 100:1) to afford the product **3d** (78.6 mg, 65% yield).

White solid; m.p.: 122~123 °C.

^{1}H NMR (600 MHz, $CDCl_3$) δ 7.86 (d, *J* = 8.4 Hz, 2H), 7.59 (d, *J* = 8.4 Hz, 2H), 7.51 (d, *J* = 7.8 Hz, 2H), 7.34 (dd, *J* = 7.8, 1.8 Hz, 2H), 7.29 (d, *J* = 8.4 Hz, 2H), 7.23–7.20 (m, 2H), 7.13 (dd, *J* = 7.8, 0.6Hz, 2H), 7.03 (td, *J* = 7.2, 1.2 Hz, 2H), 4.88 (t, *J* = 6.6 Hz, 1H), 3.38 (d, *J* = 6.6 Hz, 2H), 2.70 (q, *J* = 7.8 Hz, 2H), 1.28 (s, 3H).

^{13}C NMR (151 MHz, $CDCl_3$) δ197.6, 152.5, 145.9, 144.8, 137.2, 135.6, 129.0, 128.8, 128.6, 128.0, 127.3, 127.0, 125.7, 123.6, 116.7, 49.9, 34.9, 28.7, 15.7.

HRMS (ESI) calcd. for $C_{29}H_{24}NaO_2^+$ ([M+Na]$^+$): 427.1669, found: 427.1668.

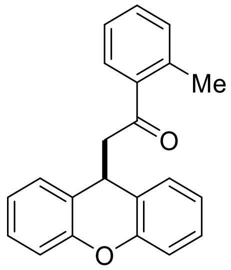

3e

1-(*o*-Tolyl)-2-(9*H*-xanthen-9-yl)ethan-1-one (3e)

Prepared following general procedure and the reaction mixture was purified by flash column chromatography with petroleum ether and ethylacetate (PE/EA = 100:1) to afford the product **3e** (49.8 mg, 53% yield).

White solid; m.p.: 105~106 °C.

^{1}H NMR (600 MHz, $CDCl_3$) δ 7.33 (d, *J* = 7.2 Hz, 2H), 7.30–7.27 (m, 2H), 7.21 (t, *J* = 7.8 Hz, 2H), 7.18 (d, *J* = 7.2 Hz, 1H), 7.12–7.09 (m, 3H), 7.04 (t, *J* = 7.2 Hz, 2H), 4.84 (t, *J* = 6.6 Hz, 1H), 3.26 (d, *J* = 6.6 Hz, 2H), 2.43 (s, 3H).

^{13}C NMR (151 MHz, $CDCl_3$) δ 202.1, 152.5, 138.2, 132.0, 131.4, 128.9, 128.6, 128.0, 125.7, 125.7, 123.6, 116.7, 52.7, 35.0, 21.2.

HRMS (ESI) calcd. for $C_{22}H_{18}NaO_2^+$ ($[M+Na]^+$): 337.1199, found: 337.1201.

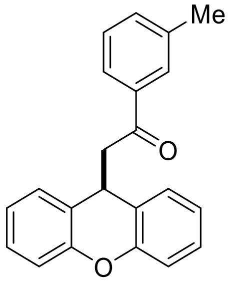

3f

1-(*m*-Tolyl)-2-(9*H*-xanthen-9-yl)ethan-1-one (3f)

Prepared following general procedure and the reaction mixture was purified by flash column chromatography with petroleum ether and ethylacetate (PE/EA = 100:1) to afford the product **3f** (60.0 mg, 68% yield).

White solid; m.p.: 111~112 °C.

^{1}H NMR (600 MHz, $CDCl_3$) δ 7.54–7.50 (m, 2H), 7.24 (t, *J* = 7.2 Hz, 2H), 7.20–7.16 (m, 2H), 7.13 (t, *J* = 7.8 Hz, 2H), 7.04 (d, *J* = 7.8 Hz, 2H), 6.95 (t, *J* = 7.2 Hz, 2H), 4.77 (t, *J* = 6.6 Hz, 1H), 3.26 (d, *J* = 6.6 Hz, 2H), 2.26 (s, 3H).

^{13}C NMR (151 MHz, $CDCl_3$) 198.3, 152.6, 138. 6, 137.2, 134.1, 129.1, 128.9, 128.6, 128.1, 125.8, 125.5, 123.7, 116.8, 50.0, 34.9, 21.5.

HRMS (ESI) calcd. for $C_{22}H_{18}NaO_2^+$ ($[M+Na]^+$): 337.1199, found 337.1198.

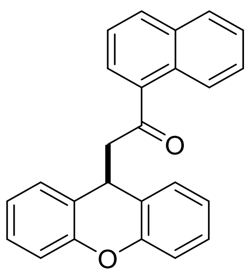

3g

1-(Naphthalen-1-yl)-2-(9*H*-xanthen-9-yl)ethan-1-one (3g)

Prepared following general procedure and the reaction mixture was purified by flash column chromatography with petroleum ether and ethylacetate (PE/EA = 100:1) to afford the product **3g** (64.2 mg, 61% yield).

White solid; m.p.: 116~117 ^{o}C.

^{1}H NMR (600 MHz, $CDCl_3$) δ 8.50 (d, *J* = 8.4 Hz, 1H), 7.91 (d, *J* = 7.8 Hz, 1H), 7.84 (d, *J* = 7.8 Hz, 1H), 7.59–7.55 (m, 1H), 7.59–7.50 (m, 1H), 7.48 (dd, *J* = 7.2, 1.1 Hz, 1H), 7.38 (dd, *J* = 7.2, 1.2 Hz, 2H), 7.36–7.32 (m, 1H), 7.24–7.21 (m, 2H), 7.12 (dd, *J* = 8.4, 1.2 Hz, 2H), 7.06–7.03 (m, 2H), 4.94 (t, *J* = 6.6 Hz, 1H), 3.42 (d, *J* = 6.6 Hz, 2H).

^{13}C NMR (151 MHz, $CDCl_3$) δ 202.2, 152.5, 136.1, 134.0, 132.9, 130.2, 128.9, 128.6, 128.1, 127.9, 126.6, 125.8, 125.5, 124.4, 123.7, 53.2, 35.5.

HRMS (ESI) calcd. for $C_{25}H_{18}NaO_2^+$ ([M+Na]$^+$): 373.1199, found: 373.1200.

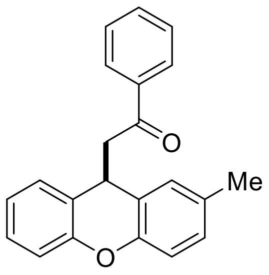

3h

2-(2-Methyl-9*H*-xanthen-9-yl)-1-phenylethan-1-one (3h)

Prepared following general procedure and the reaction mixture was purified by flash column chromatography with petroleum ether and ethylacetate (PE/EA = 100:1) to afford the product **3h** (65.8 mg, 70% yield).

White solid; m.p.: 102~103 °C.

^{1}H NMR (600 MHz, $CDCl_3$) δ 7.81 (d, *J* = 7.8 Hz, 2H), 7.50 (t, *J* = 7.2 Hz, 1H), 7.37 (t, *J* = 7.7 Hz, 2H), 7.31 (d, *J* = 7.8 Hz, 1H), 7.19 (t, *J* = 7.2 Hz, 1H), 7.10 (d, *J* = 7.5 Hz, 2H), 7.02–6.89 (m, 3H), 4.80 (t, *J* = 6.6 Hz, 1H), 3.35 (d, *J* = 6.6 Hz, 2H), 2.27 (s, 3H).

^{13}C NMR (151 MHz, $CDCl_3$) δ 198.2, 152.6, 150.3, 137.2, 133.21, 133.0, 129.2, 129.0, 128.6, 128.6, 128.2, 127.9, 125.7, 125.3, 123.4, 116.6, 116.4, 49.9, 34.8, 20.8.

HRMS (ESI) calcd. for $C_{22}H_{18}NaO_2^+$ ($[M+Na]^+$): 337.1199, found: 337.1199.

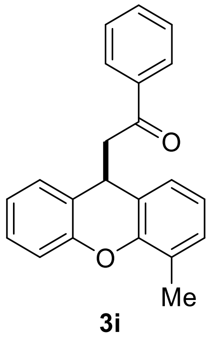

3i

2-(4-Methyl-9*H*-xanthen-9-yl)-1-phenylethan-1-one (3i)

Prepared following general procedure and the reaction mixture was purified by flash column chromatography with petroleum ether and ethylacetate (PE/EA = 100:1) to afford the product **3i** (67.8 mg, 72% yield).

White solid; m.p.: 107~108 °C.

^{1}H NMR (600 MHz, $CDCl_3$) δ 7.71 (d, *J* = 8.2 Hz, 2H), 7.33 (d, *J* = 7.6 Hz, 2H), 7.23 –7.20 (m, 2H), 7.17 (d, *J* = 8.2 Hz, 2H), 7.12 (d, *J* = 8.1 Hz, 2H), 7.02 (t, *J* = 7.4 Hz, 2H), 4.86 (t, *J* = 6.6 Hz, 1H), 3.33 (d, *J* = 6.6 Hz, 2H), 2.36 (s, 3H).

^{13}C NMR (151 MHz, $CDCl_3$) δ197.5, 152.3, 143.9, 134.5, 130.0, 129.8, 129.5, 129.5, 129.2, 128.8, 128.3, 128.2, 127.8, 127.3, 125.6, 123.4, 116.5, 49.6, 34.7, 21.6.

HRMS (ESI) calcd. for $C_{22}H_{18}NaO_2^+$ ($[M+Na]^+$): 337.1199, found: 337.1196.

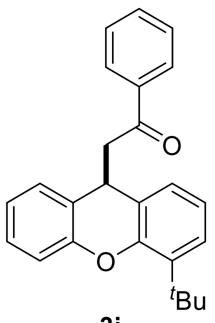

3j

2-(4-(*tert*-Butyl)-9*H*-xanthen-9-yl)-1-phenylethan-1-one (3j)

Prepared following general procedure and the reaction mixture was purified by flash column chromatography with petroleum ether and ethylacetate (PE/EA = 100:1) to afford the product **3j** (74.0 mg, 70% yield).

White solid; m.p.: 113~114 ^{o}C.

^{1}H NMR (600 MHz, $CDCl_3$) δ 7.78–7.75 (m, 2H), 7.41–7.38 (m, 2H), 7.32 (dd, J = 7.8, 1.2 Hz, 2H), 7.22–7.19 (m, 2H), 7.12 (dd, J = 8.4, 1.2 Hz, 2H), 7.02 (td, J = 7.2, 1.2 Hz, 2H), 4.87 (t, J = 6.6 Hz, 1H), 3.34 (d, J = 6.6 Hz, 2H), 1.30 (s, 9H).

^{13}C NMR (151 MHz, $CDCl_3$) δ 196.4, 155.9, 151.3, 133.4, 128.8, 128.7, 127.8, 127.3, 127.0, 126.8, 126.3, 124.7, 124.6, 124.4, 122.4, 121.8, 115.5, 48.7, 34.0, 33.5, 29.9.

HRMS (ESI) calcd. for $C_{25}H_{24}NaO_2^+$ ($[M+Na]^+$): 379.1669, found: 379.1670.

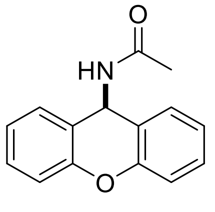

4a

***N*-(9*H*-xanthen-9-yl)acetamide** (**4a**)

Prepared following general procedure and the reaction mixture was purified by flash column chromatography with petroleum ether and ethylacetate (PE/EA=5:1) to afford the product **2a** (48.7 mg, 68% yield).

White solid; m.p.: 238~239 °C.

^{1}H NMR (600 MHz, $CDCl_3$) δ 7.48–7.46 (m, 2H), 7.32–7.28 (m, 2H), 7.13–7.10 (m, 4H), 6.49 (d, *J* = 9.0 Hz, 1H), 6.05 (br, d, *J* = 9.0 Hz, 1H), 2.00 (s, 3H).

^{13}C NMR (151 MHz, $CDCl_3$) δ 169.2, 151.3, 129.8, 129.4, 123.7, 121.3, 116.8, 44.0, 23.5.

HRMS (ESI) calcd. for $C_{15}H_{13}NNaO_2^+$ ([M+Na]$^+$): 262.0838, found: 262.0839.

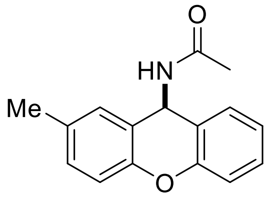

4b

***N*-(2-Methyl-9*H*-xanthen-9-yl)acetamide** (**4b**)

Prepared following general procedure and the reaction mixture was purified by flash column chromatography with petroleum ether and ethylacetate (PE/EA=5:1) to afford the product **2b** (49.5 mg, 65% yield).

White solid; m.p.: 242~243 °C.

^{1}H NMR (600 MHz, $CDCl_3$) δ 7.46 (d, *J* = 7.8 Hz, 1H), 7.31–7.27 (m, 1H), 7.11–7.08 (m, 3H), 7.00 (d, *J* = 8.4 Hz, 1H), 6.46 (d, *J* = 9.0 Hz, 1H), 6.00 (br, d, *J* = 9.0 Hz , 1H), 2.32 (s, 3H), 2.01 (s, 3H).

^{13}C NMR (151 MHz, $CDCl_3$) δ 169.2, 151.4, 149.2, 133.2, 130.2, 129.8, 129.8, 129.3, 123.5, 121.3, 120.8, 116.7, 116.5, 44.1, 23.6, 20.8.

HRMS (ESI) calcd. for $C_{16}H_{15}NNaO_3^+$ ([M+Na]$^+$): 276.0095, found: 276.0092.

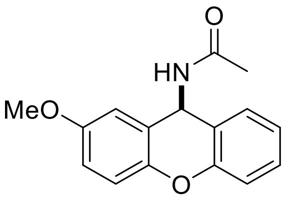

4c

***N*-(2-Methoxy-9*H*-xanthen-9-yl)acetamide (4c)**

Prepared following general procedure and the reaction mixture was purified by flash column chromatography with petroleum ether and ethylacetate (PE/EA = 5:1) to afford the product **4c** (60.3 mg, 75% yield).

White solid; m.p.: 251~252 ^{o}C.

^{1}H NMR (600 MHz, $CDCl_3$) δ 7.46 (d, *J* = 7.2 Hz, 1H), 7.31–7.27 (m, 1H), 7.11–7.07 (m, 2H), 7.04 (d, *J* = 9.0 Hz, 1H), 6.98 (d, *J* = 3.0 Hz, 1H), 6.87 (dd, *J* = 9.0, 3.0 Hz, 1H), 6.47 (d, *J* = 9.0 Hz, 1H), 6.01 (br, d, *J* = 9.0 Hz, 1H), 3.79 (s, 3H), 2.01 (s, 3H).

^{13}C NMR (151 MHz, $CDCl_3$) δ 169.2, 155.7, 151.5, 145.4, 129.8, 129.4, 123.5, 121.7, 120.7, 117.7, 116.7, 116.4, 112.8, 55.9, 44.5, 23.6.

HRMS (ESI) calcd. for $C_{16}H_{15}NNaO_3^+$ ([M+Na]$^+$): 292.0944, found 292.0947.

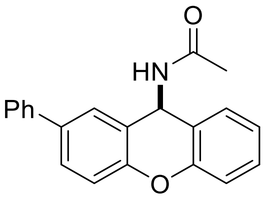

4d

***N*-(2-Phenyl-9*H*-xanthen-9-yl)acetamide (4d)**

Prepared following general procedure and the reaction mixture was purified by flash column chromatography with petroleum ether and ethylacetate (PE/EA = 5:1) to afford the product **4d** (57.7 mg, 61% yield).

White solid; m.p.: 247~248 ^{o}C.

^{1}H NMR (600 MHz, $CDCl_3$) δ 7.70 (d, *J* = 1.8 Hz, 1H), 7.58–7.56 (m, 2H), 7.54 (dd, *J* = 9.0, 2.4 Hz, 1H), 7.51–7.49 (m, 1H), 7.43 (t, *J* = 7.2 Hz, 2H), 7.36–7.30 (m, 2H), 7.18 (d, *J* = 8.4 Hz, 1H), 7.14–7.11 (m, 2H), 6.57 (d, *J* = 9.6 Hz, 1H), 6.06 (br, *J* = 9.6 Hz, 1H), 2.01 (s, 3H).

^{13}C NMR (151 MHz, $CDCl_3$) δ169.2, 151.2, 150.8, 140.2, 136.9, 129.9, 129.5, 129.0, 128.2, 128.2, 127.4, 127.0, 123.8, 121.5, 121.2, 117.2, 116.8, 44.1, 23.6.

HRMS (ESI) calcd. for $C_{21}H_{17}NNaO_2^+$ ($[M+Na]^+$): 338.1151, found: 338.1147.

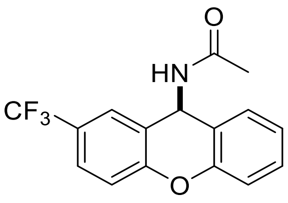

4e

***N*-(2-(Trifluoromethyl)-9*H*-xanthen-9-yl)acetamide (4e)**

Prepared following general procedure and the reaction mixture was purified by flash column chromatography with petroleum ether and ethylacetate (PE/EA = 5:1) to afford the product **4e** (51.6 mg, 56% yield).

White solid; m.p.: 244~245 ^{o}C.

^{1}H NMR (600 MHz, $CDCl_3$) δ7.75 (s, 1H), 7.55 (dd, *J* = 8.4, 1.2 Hz, 1H), 7.45 (d, *J* = 8.4 Hz, 1H), 7.35–7.31 (m, 1H), 7.20 (d, *J* = 8.4 Hz, 1H), 7.15 (t, *J* = 7.8 Hz, 1H), 7.13 (d, *J* = 8.4 Hz, 1H), 6.52 (d, *J* = 9.0Hz, 1H), 6.10 (br, s, 1H), 2.04 (s, 3H).

^{13}C NMR (151 MHz, $CDCl_3$) δ169.4, 153.5, 150.7, 129.8, 129.64, 127.4 (q, *J* = 3.6), 126.5 (q, *J* = 3.2), 125.9 (q, *J* = 271.5), 124.5, 123.1, 121.8, 121.3, 120.7, 117.4, 116.9, 43.7, 23.5.

^{19}F NMR (565 MHz, $CDCl_3$) δ -77.30.

HRMS (ESI) calcd. for $C_{16}H_{12}F_3NNaO_2^+$ ($[M+Na]^+$): 330.0172, found: 330.0175.

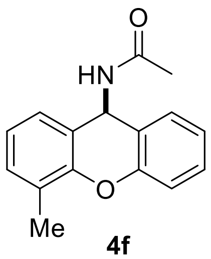

4f

N-(4-Methyl-9*H*-xanthen-9-yl)acetamide (4f)

Prepared following general procedure and the reaction mixture was purified by flash column chromatography with petroleum ether and ethylacetate (PE/EA = 5:1) to afford the product **4f** (52.5 mg, 69% yield).

White solid; m.p.: 239~240 ^{o}C.

^{1}H NMR (600 MHz, $CDCl_3$) 7.50 (d, *J* = 7.2 Hz, 1H), 7.31 (t, *J* = 8.4 Hz, 2H), 7.16 (d, *J* = 8.4 Hz, 2H), 7.13–7.10 (m, 1H), 7.02 (t, *J* = 7.8 Hz, 1H), 6.50 (d, *J* = 9.0 Hz, 1H), 5.92 (br, d, *J* = 8.4 Hz, 1H), 2.40 (s, 3H), 2.00 (s, 3H).

^{13}C NMR (151 MHz, $CDCl_3$) δ 169.0, 151.2, 151.0, 139.6, 129.7, 129.4, 129.2, 124.6, 123.5, 121.3, 118.1, 116.9, 116.6, 43.8, 23.4, 21.2.

HRMS (ESI) calcd. for $C_{16}H_{15}NNaO_2^+$ ($[M+Na]^+$): 276.0995, found: 276.0997.

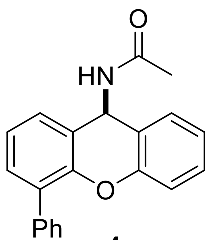

4g

N-(4-Phenyl-9*H*-xanthen-9-yl)acetamide (4g)

Prepared following general procedure and the reaction mixture was purified by flash column chromatography with petroleum ether and ethylacetate (PE/EA = 5:1) to afford the product **4g** (54.7 mg, 58% yield).

White solid; m.p.: 251~152 ^{o}C.

^{1}H NMR (600 MHz, $CDCl_3$) δ 7.60 (d, *J* = 7.0 Hz, 2H), 7.51–7.46 (m, 4H), 7.42–7.38 (m, 1H), 7.36 (dd, *J* = 7.8, 1.6 Hz, 1H), 7.29–7.26 (m, 1H), 7.19 (t, *J* = 7.8 Hz, 1H), 7.12 (td, *J* = 7.8, 1.2 Hz, 1H), 7.03 (d, *J* = 8.4 Hz, 1H), 6.56 (d, *J* = 9.0 Hz, 1H), 6.06 (br, *J* = 8.4 Hz, 1H), 2.02 (s, 3H).

^{13}C NMR (151 MHz, $CDCl_3$) δ169.2, 151.3, 148.3, 137.5, 130.8, 130.2, 129.8, 129.6, 129.3, 129.1, 128.3, 127.5, 123.9, 123.7, 122.0, 121.3, 116.9, 44.5, 23.6.

HRMS (ESI) calcd. for $C_{21}H_{17}NNaO_2^+$ ($[M+Na]^+$): 338.1151, found: 338.1151.

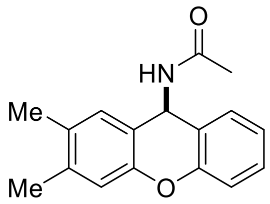

4h

***N*-(2,3-Dimethyl-9,10-dihydroanthracen-9-yl)acetamide (4h)**

Prepared following general procedure and the reaction mixture was purified by flash column chromatography with petroleum ether and ethylacetate (PE/EA = 5:1) to afford the product **4h** (57.2 mg, 72% yield).

White solid; m.p.: 247~248 °C.

^{1}H NMR (600 MHz, $CDCl_3$) δ 7.66 (dd, *J* = 7.8, 0.6 Hz, 1H), 7.30–7.27 (m, 1H), 7.12 (d, *J* = 8.4 Hz, 1H), 7.10–7.07 (m, 2H), 6.92 (d, *J* = 8.4 Hz, 1H), 6.55 (d, *J* = 9.0 Hz, 1H), 5.85 (br, d, *J* = 7.8 Hz, 1H), 2.27 (s, 3H), 2.26 (s, 3H), 1.91 (s, 3H).

^{13}C NMR (151 MHz, $CDCl_3$) δ168.4, 151.1, 150.8, 136.8, 131.9, 130.7, 130.2, 129.2, 123.6, 122.8, 119.0, 116.3, 114.1, 42.9, 23.3, 20.2, 15.2.

HRMS (ESI) calcd. for $C_{17}H_{17}NNaO_2^+$ ($[M+Na]^+$): 290.1151, found: 290.1152.

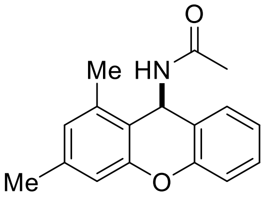

4i

N-(1,3-Dimethyl-9,10-dihydroanthracen-9-yl)acetamide (4i)

Prepared following general procedure and the reaction mixture was purified by flash column chromatography with petroleum ether and ethylacetate (PE/EA = 5:1) to afford the product **4i** (51.6 mg, 67% yield).

White solid; m.p.: 244~245 ^{o}C.

^{1}H NMR (600 MHz, $CDCl_3$) δ 7.66–7.63 (m, 1H), 7.29 (td, *J* = 7.8, 1.8 Hz, 1H), 7.11–7.08 (m, 2H), 6.82 (d, *J* = 4.8 Hz, 2H), 6.47 (d, *J* = 9.6 Hz, 1H), 5.78 (br, d, *J* = 9.0 Hz, 1H), 2.34 (s, 3H), 2.32 (s, 3H), 1.91 (s, 3H).

^{13}C NMR (151 MHz, $CDCl_3$) δ 168.4, 152.4, 151.3, 139.4, 138.6, 130.2, 129.2, 126.4, 123.7, 122.9, 116.5, 116.2, 115.1, 42.4, 23.4, 21.2, 18.7.

HRMS (ESI) calcd. for $C_{17}H_{17}NNaO_2^+$ ([M+Na]$^+$): 290.1151, found: 290.1155.

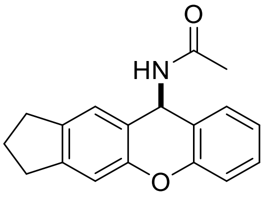

4j

N-(1,2,3,10-Tetrahydrocyclopenta[*b*]xanthen-10-yl)acetamide (4j)

Prepared following general procedure and the reaction mixture was purified by flash column chromatography with petroleum ether and ethylacetate (PE/EA = 5:1) to afford the product **4j** (51.9 mg, 70% yield).

White solid; m.p.: 236~237 ^{o}C.

^{1}H NMR (600 MHz, $CDCl_3$) δ 7.58 (dd, *J* = 8.4, 1.8 Hz, 1H), 7.30–7.27 (m, 1H), 7.18 (d, *J* = 8.4 Hz, 1H), 7.11–7.07 (m, 2H), 6.94 (d, *J* = 8.4 Hz, 1H), 6.48 (d, *J* = 9.6 Hz, 1H), 5.81 (br, d, *J* = 9.6 Hz, 1H), 2.99–2.94 (m, 1H), 2.90 (t, *J* = 8.4 Hz, 2H), 2.87–2.81 (m, 1H), 2.17–2.08 (m, 2H), 1.96 (s, 3H).

^{13}C NMR (151 MHz, $CDCl_3$) δ 168.5, 151.327, 150.6, 144.8, 139.4, 130.2, 129.3, 125.1, 123.6, 122.2, 116.6, 116.4, 114.9, 42.8, 32.5, 31.4, 25.7, 23.4.

HRMS (ESI) calcd. for $C_{18}H_{17}NNaO_2^+$ ($[M+Na]^+$): 302.1151, found 302.1154.

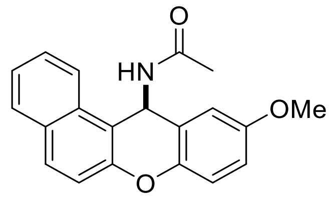

4k

***N*-(10-Methoxy-12*H*-benzo[a]xanthen-12-yl)acetamide (4k)**

Prepared following general procedure and the reaction mixture was purified by flash column chromatography with petroleum ether and ethylacetate (PE/EA = 3:1) to afford the product **4k** (61.3 mg, 64% yield).

White solid; m.p.: 258~259 °C.

^{1}H NMR (600 MHz, $CDCl_3$) δ 8.07 (d, *J* = 8.4 Hz, 1H), 7.84 (d, *J* = 6.6 Hz, 1H), 7.82 (d, *J* = 8.4 Hz, 1H), 7.60 (t, *J* = 6.6 Hz, 1H), 7.46 (t, *J* = 7.2Hz, 1H), 7.30 (d, *J* = 9.0 Hz, 1H), 7.25 (d, *J* = 3.0 Hz, 1H), 7.13 (d, *J* = 9.0 Hz, 1H), 7.08 (d, *J* = 9.4 Hz, 1H), 6.92 (dd, *J* = 8.4, 3.0 Hz, 1H), 5.85 (br, d, *J* = 9.6 Hz, 1H), 3.83 (s, 3H), 1.92 (s, 3H).

^{13}C NMR (151 MHz, $CDCl_3$) δ 168.7, 156.2, 150.3, 144.8, 131.8, 130. 5, 130.4, 128.7, 127.8, 124.8, 123.0, 122.5, 118.0, 117.6, 116.8, 112.6, 111.5, 56.0, 42.2, 23.4.

HRMS (ESI) calcd. for $C_{20}H_{17}NNaO_2^+$ ($[M+Na]^+$): 342.1101, found 342.1102.

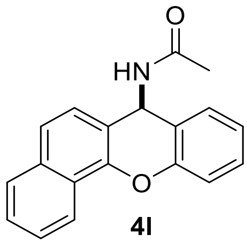

4l

N-(7H-benzo[c]xanthen-7-yl)acetamide (4l)

Prepared following general procedure and the reaction mixture was purified by flash column chromatography with petroleum ether and ethylacetate (PE/EA = 5:1) to afford the product **4l** (47.6 mg, 55% yield).

White solid; m.p.: 250~251 °C.

^{1}H NMR (600 MHz, $CDCl_3$)δ 8.43 (d, *J* = 8.4 Hz, 1H), 7.83 (d, *J* = 7.2 Hz, 1H), 7.60 –7.54 (m, 4H), 7.51–7.48 (m, 1H), 7.38–7.35 (m, 1H), 7.30 (dd, *J* = 8.4, 1.2 Hz, 1H), 7.19–7.16 (m, 1H), 6.66 (d, *J* = 9.0 Hz, 1H), 6.04 (br, d, *J* = 9.0 Hz, 1H), 2.02 (s, 3H).

^{13}C NMR (151 MHz, $CDCl_3$) δ 169.1, 151.0, 146.3, 134.0, 129.9, 129.3, 127.6, 127.0, 126.3, 126.3, 124.0, 123.9, 123.3, 121.8, 121.2, 116.8, 114.8, 44.2, 23.5.

HRMS (ESI) calcd. for $C_{19}H_{15}NNaO_2^+$ ([M+Na]$^+$): 312.0095, found 312.0098.

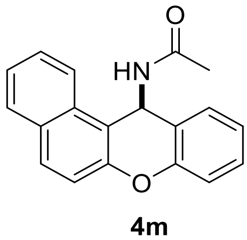

4m

N-(12H-benzo[a]xanthen-12-yl)acetamide (4m)

Prepared following general procedure and the reaction mixture was purified by flash column chromatography with petroleum ether and ethylacetate (PE/EA = 5:1) to afford the product **4m** (50.3 mg, 58% yield).

White solid; m.p.: 249~250 °C.

^{1}H NMR (600 MHz, $CDCl_3$) δ 8.43 (d, *J* = 8.4 Hz, 1H), 7.83 (d, *J* = 7.8 Hz, 1H), 7.60–7.54 (m, 4H), 7.50 (d, *J* = 8.4 Hz, 1H), 7.37 (t, *J* = 7.8 Hz, 1H), 7.30 (d, *J* = 7.8 Hz, 1H), 7.17 (t, *J* = 7.2 Hz, 1H), 6.67 (d, *J* = 9.6 Hz, 1H), 6.01 (br, *J* = 9.0 Hz, 1H), 2.02 (s, 3H).

^{13}C NMR (151 MHz, $CDCl_3$) δ 169.1, 151.1, 146.4, 134.1, 130.0, 129.4, 127.8, 127.1, 126.4, 126.4, 124.1, 123.4, 121.9, 121.4, 116.9, 114.9, 44.4, 23.6.

HRMS (ESI) calcd. for $C_{19}H_{15}NNaO_2^+$ ($[M+Na]^+$): 312.0095, found 312.0098.

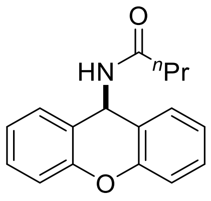

4n

***N*-(9*H*-xanthen-9-yl)butyramide (4n)**

Prepared following general procedure and the reaction mixture was purified by flash column chromatography with petroleum ether and ethylacetate (PE/EA = 9:1) to afford the product **4n** (40.1 mg, 50% yield).

White solid; m.p.: 262~263 °C.

^{1}H NMR (600 MHz, $CDCl_3$) δ 7.45 (d, *J* = 7.8 Hz, 2H), 7.30 (t, *J* = 7.2 Hz, 2H), 7.12–7.09 (m, 4H), 6.52 (d, *J* = 9.0 Hz, 1H), 6.05 (br, d, *J* = 8.4 Hz, 1H), 2.17 (t, *J* = 7.8 Hz, 2H), 1.72–1.67 (m, 2H), 0.94 (t, *J* = 7.2 Hz, 3H).

^{13}C NMR (151 MHz, $CDCl_3$) δ 172.2, 151.2, 129.8, 129.4, 123.7, 121.4, 116.8, 43.8, 38.9, 19.3, 13.9.

HRMS (ESI) calcd. for $C_{18}H_{19}NO_2^+$ ($[M+H]^+$): 267.1379, found: 267.1377.

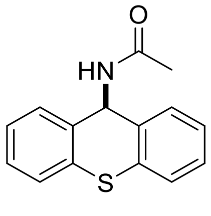

4o

***N*-(9*H*-thioxanthen-9-yl)acetamide (4o)**

Prepared following general procedure and the reaction mixture was purified by flash column chromatography with petroleum ether and ethylacetate (PE/EA = 5:1) to afford the product **4o** (48.9 mg, 64% yield).

White solid; m.p.: 237~238 ^{o}C.

^{1}H NMR (600 MHz, $CDCl_3$) δ 7.58 (dd, *J* = 6.0, 2.4 Hz, 2H), 7.47–7.45 (m, 2H), 7.28–7.25 (m, 4H), 6.30 (d, *J* = 9.0 Hz, 1H), 6.23 (br, d, *J* = 7.2 Hz, 1H), 1.88 (s, 3H).

^{13}C NMR (151 MHz, $CDCl_3$) δ169.0, 134.9, 133.0, 129.6, 128.0, 127.2, 127.2, 53.4, 23.5.

HRMS (ESI) calcd. for $C_{15}H_{13}NNaOS^+$ ($[M+Na]^+$): 278.0610, found: 278.0613.

5. NMR Spectra of the Products

NMR spectra of 1-phenyl-2-(9*H*-xanthen-9-yl)ethan-1-one (**3a**)

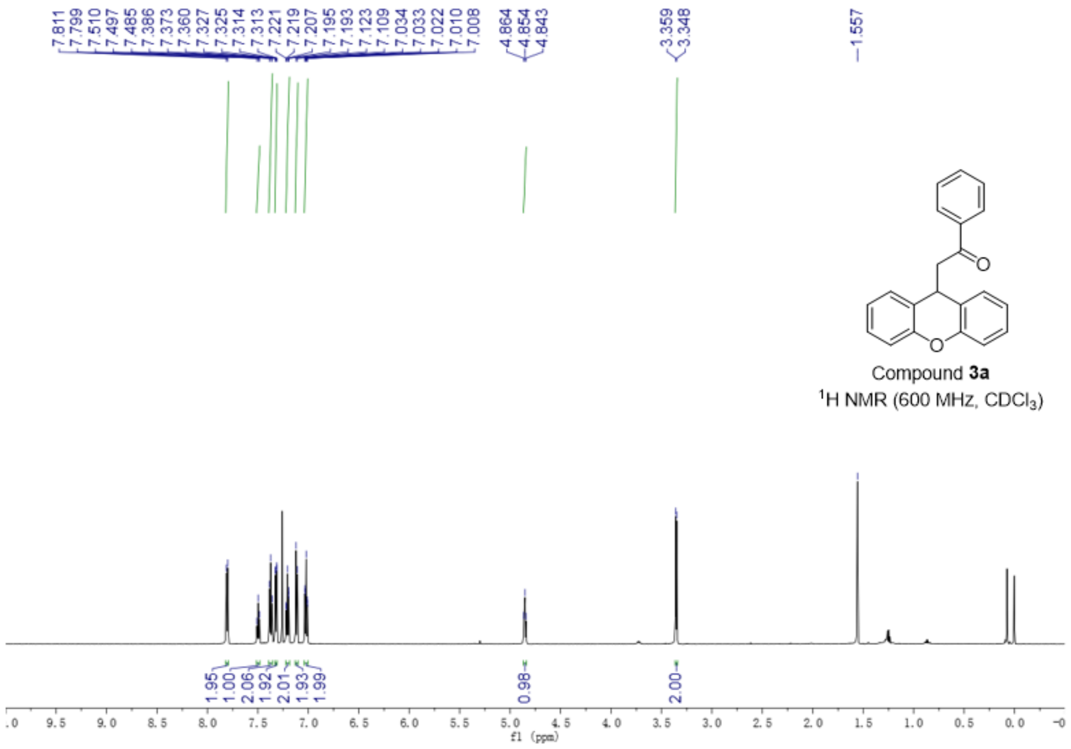

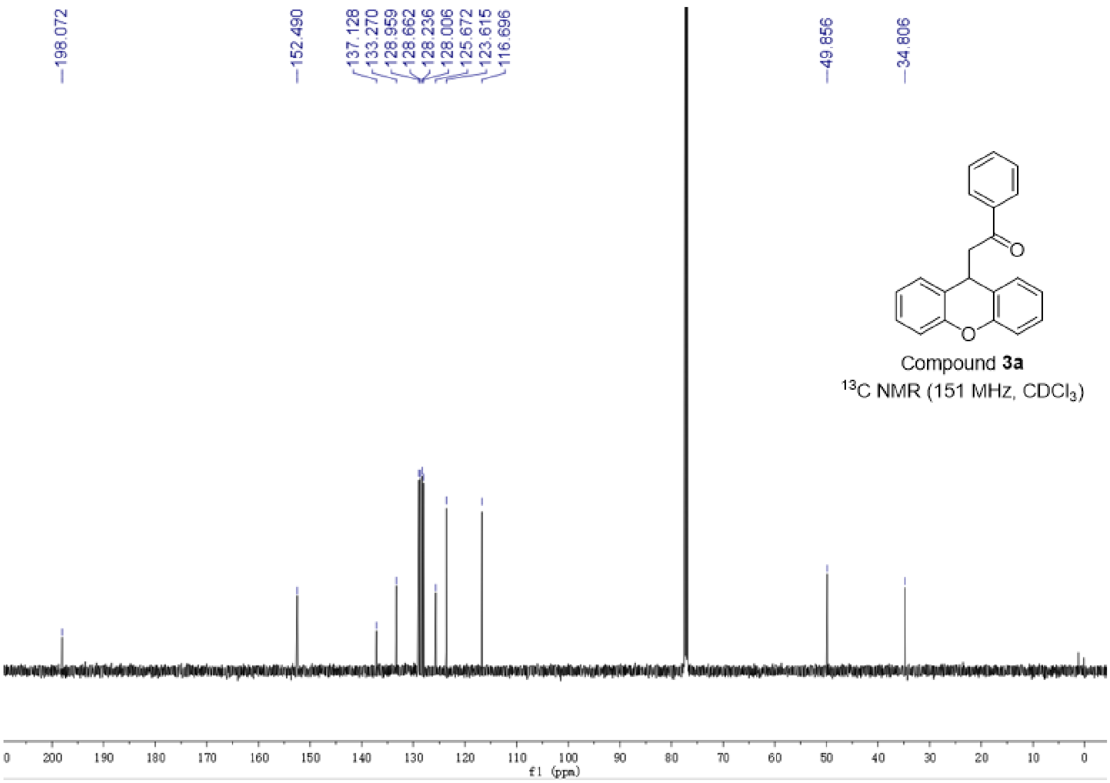

NMR spectra of 1-(*p*-tolyl)-2-(9*H*-xanthen-9-yl)ethan-1-one (**3b**)

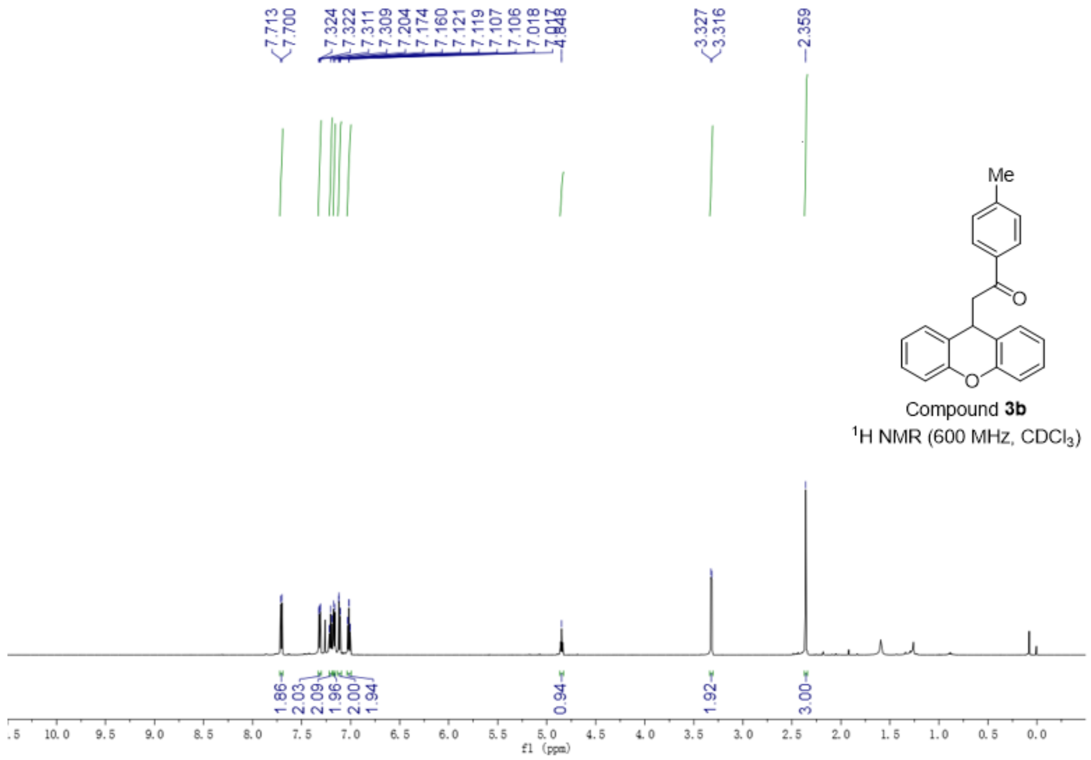

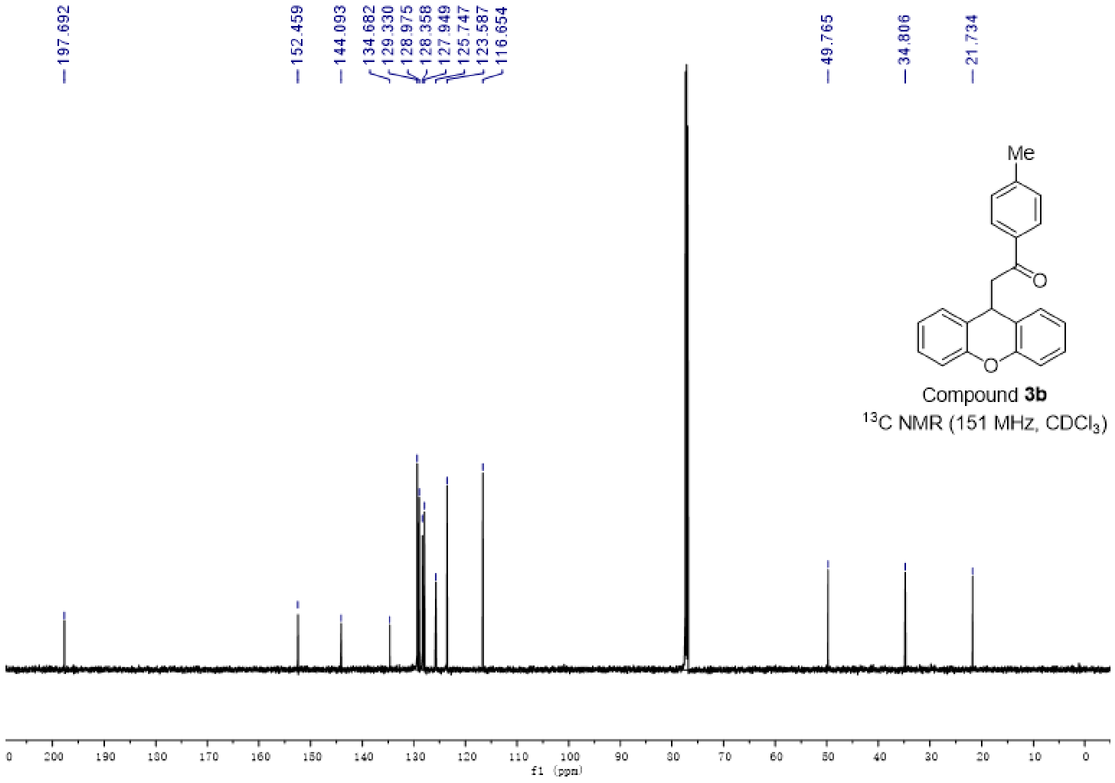

NMR spectra of 1-(4-pentylphenyl)-2-(9*H*-xanthen-9-yl)ethan-1-one (**3c**)

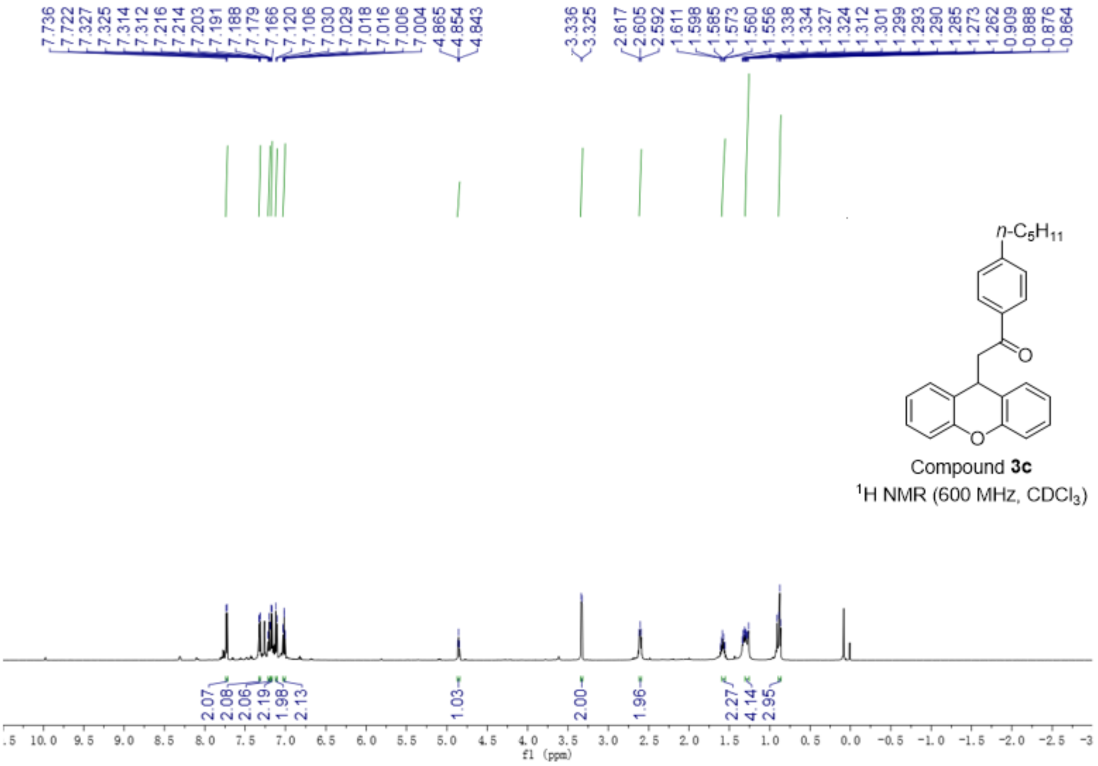

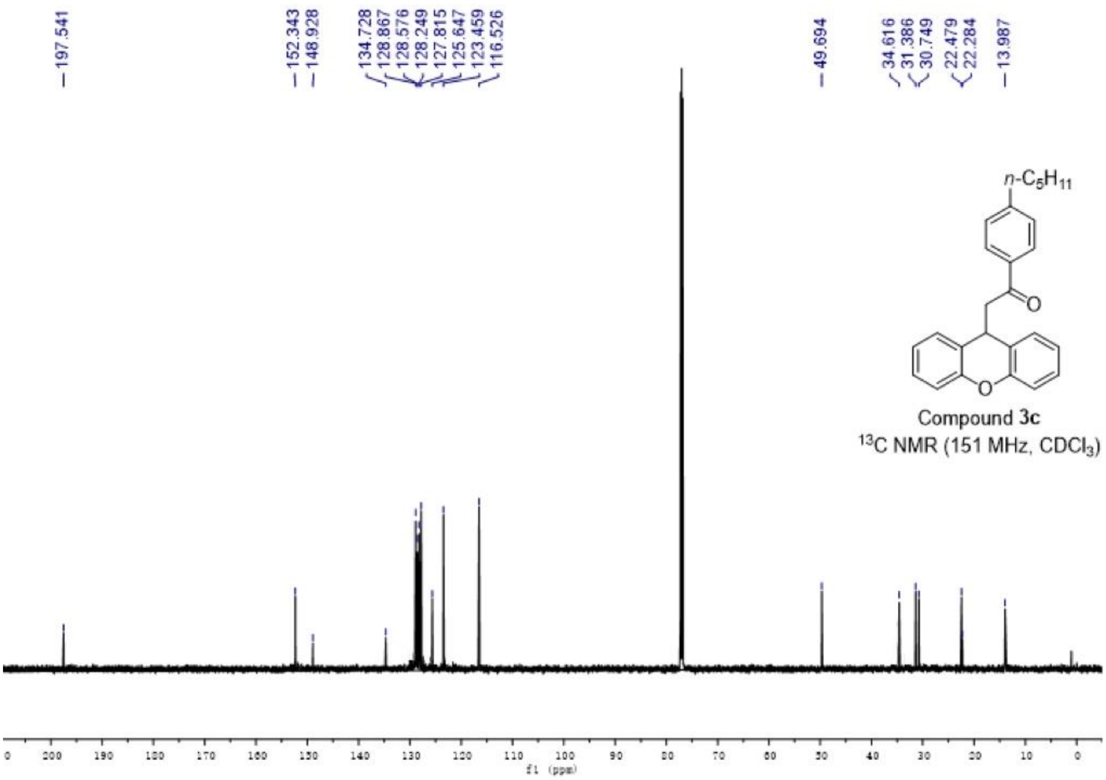

NMR spectra of 1-(4'-ethyl-[1,1'-biphenyl]-4-yl)-2-(9*H*-xanthen-9-yl)ethan-1-one (**3d**)

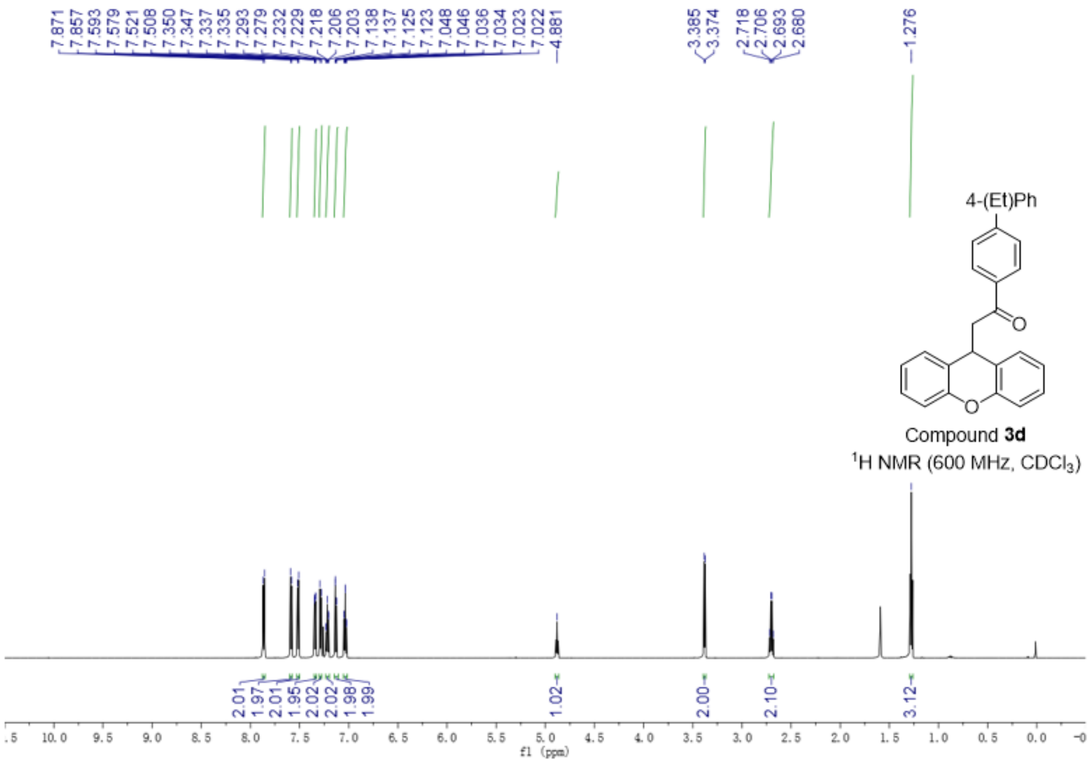

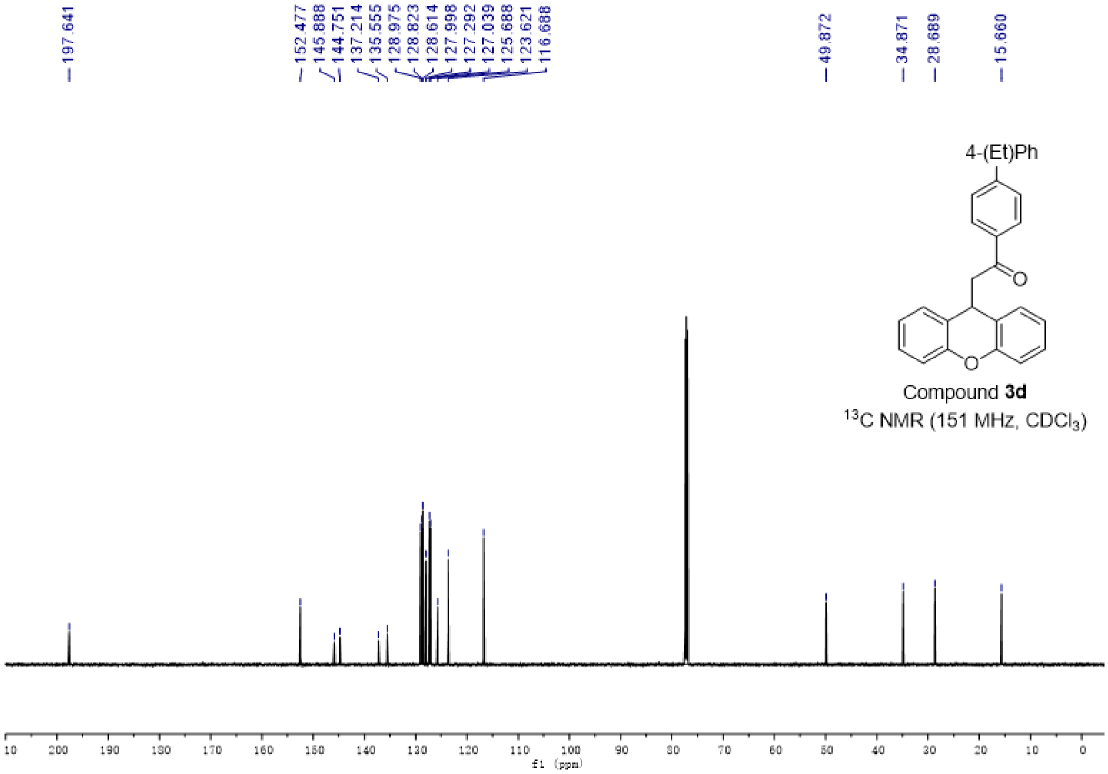

NMR spectra of 1-(*o*-tolyl)-2-(9*H*-xanthen-9-yl)ethan-1-one **(3e)**

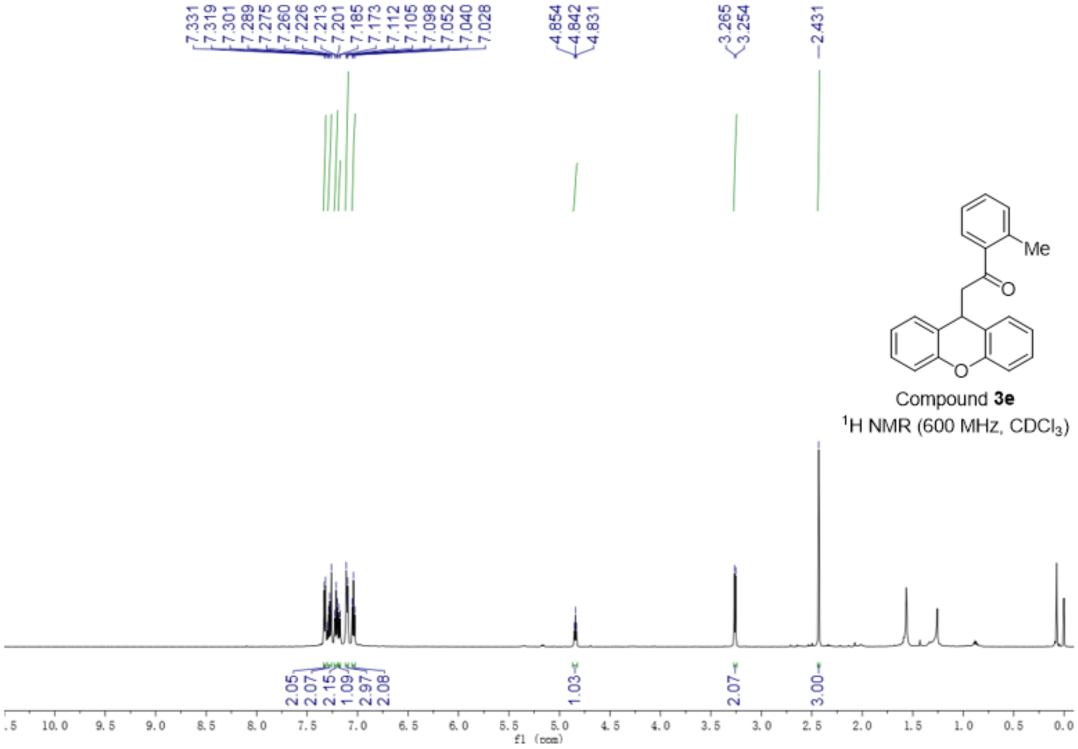

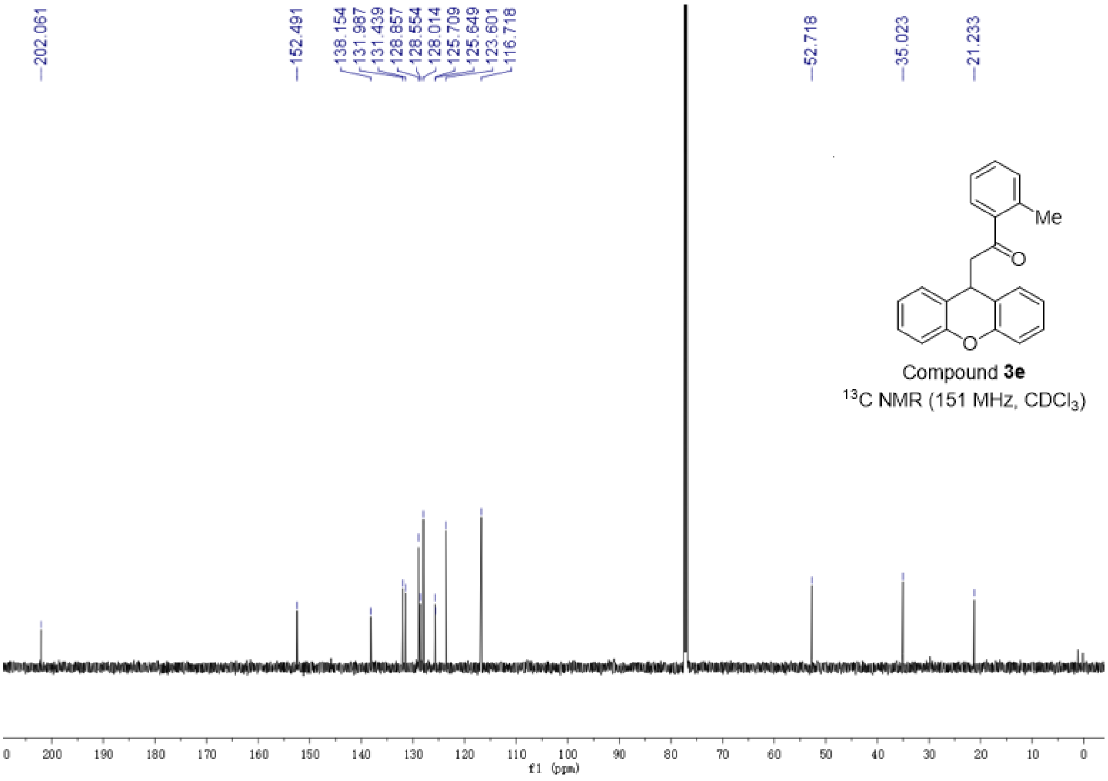

NMR spectra of 1-(*m*-tolyl)-2-(9*H*-xanthen-9-yl)ethan-1-one (**3f**)

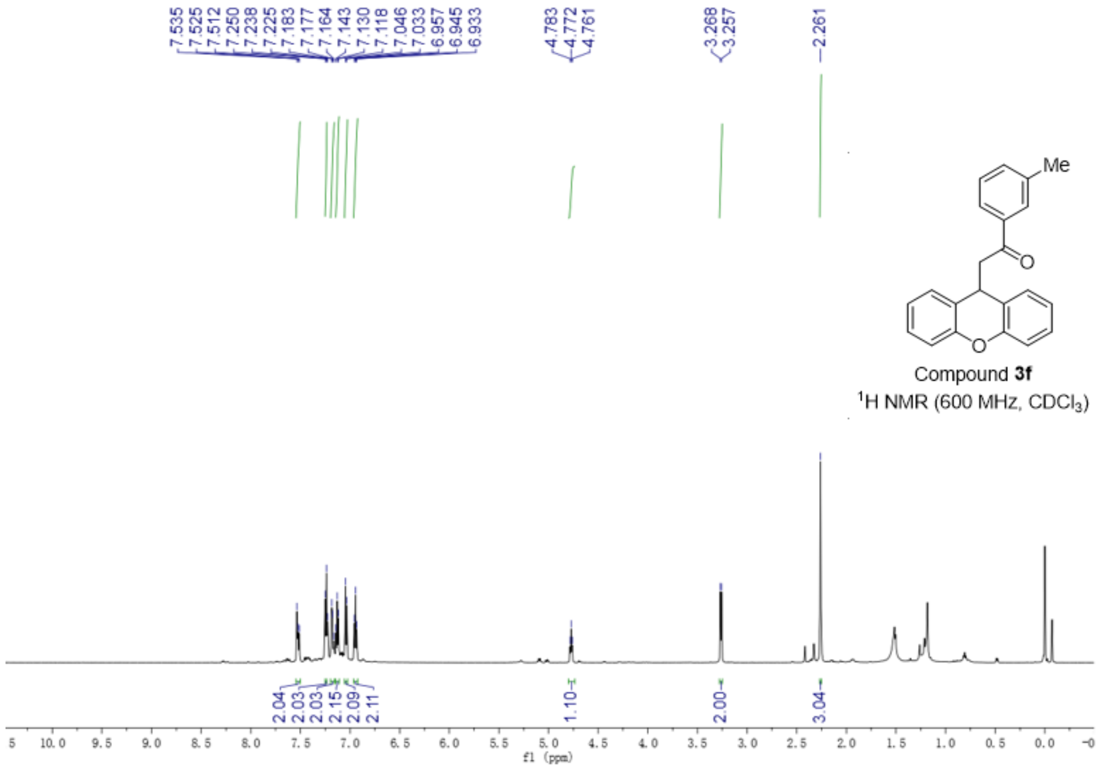

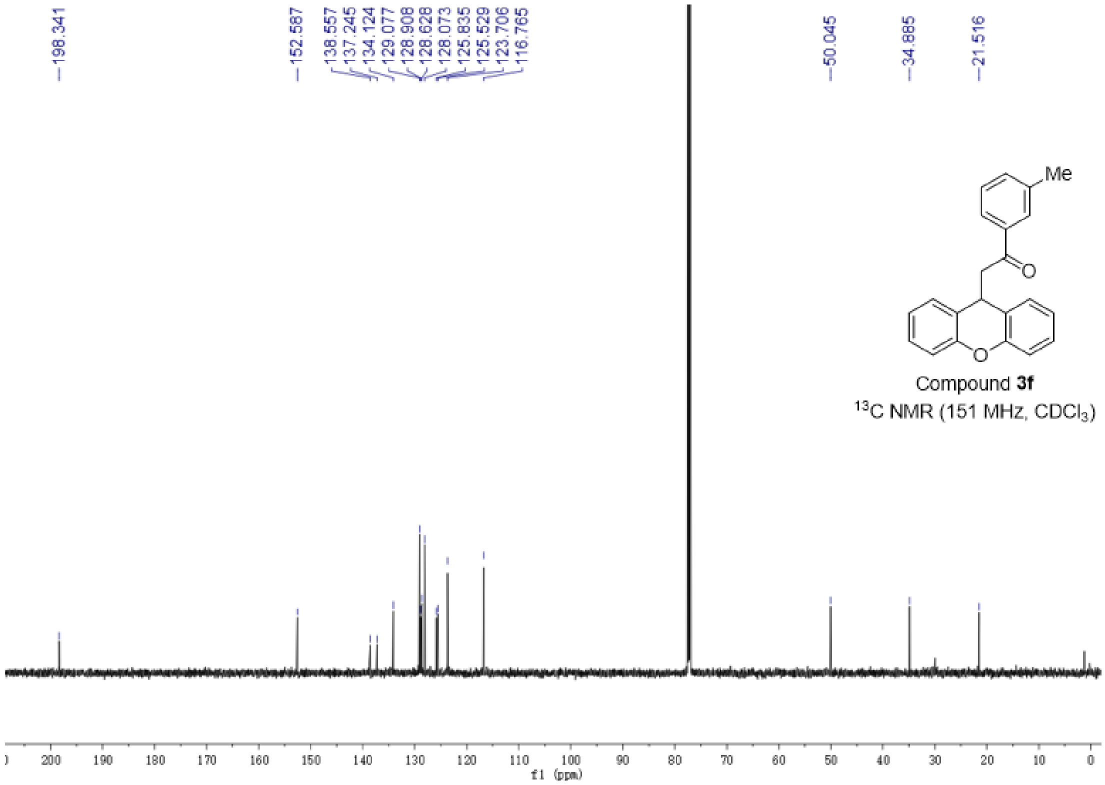

NMR spectra of 1-(naphthalen-1-yl)-2-(9*H*-xanthen-9-yl)ethan-1-one (**3g**)

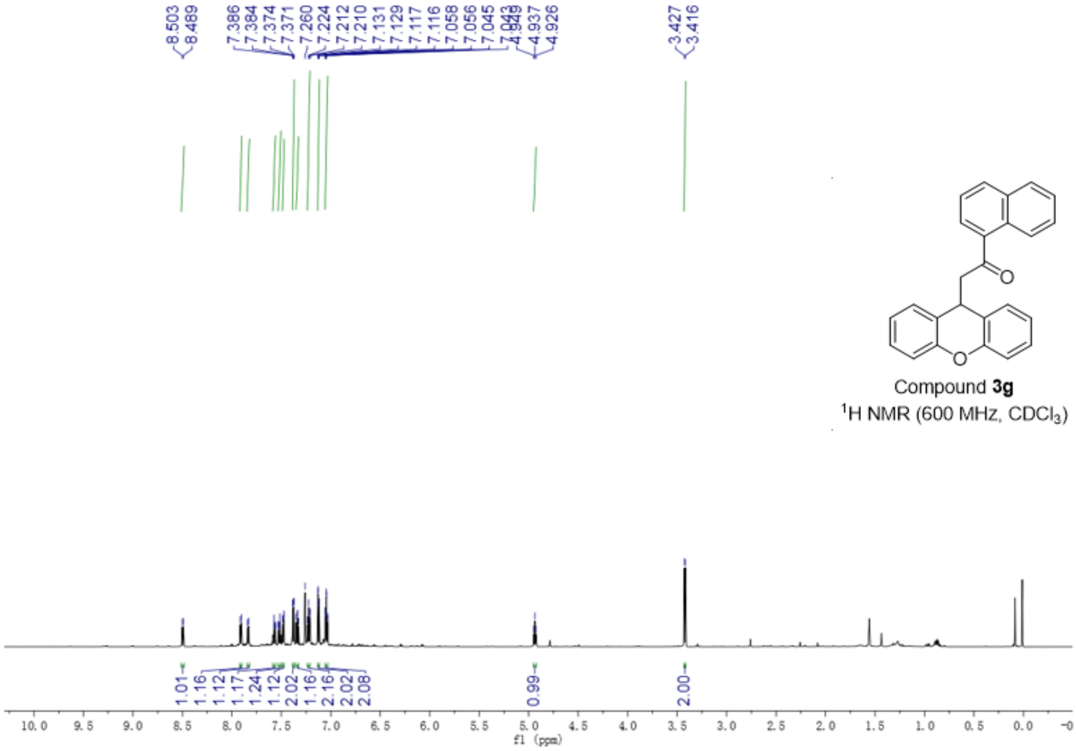

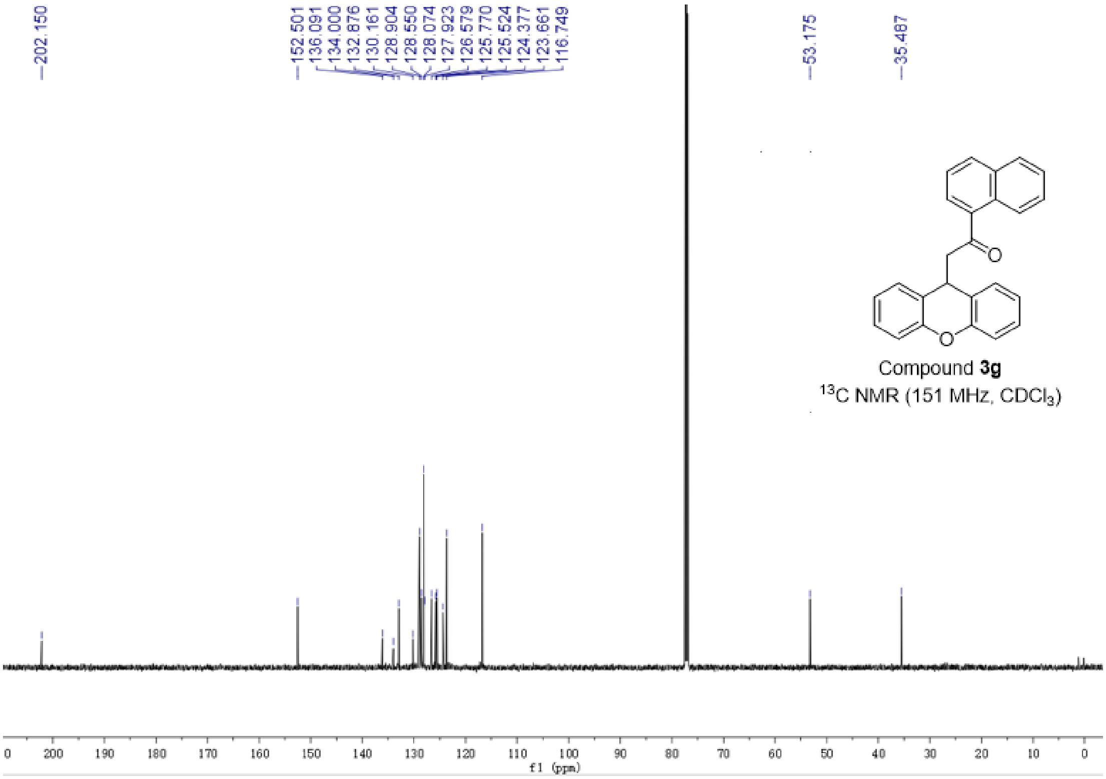

NMR spectra of 2-(2-methyl-9*H*-xanthen-9-yl)-1-phenylethan-1-one (**3h**)

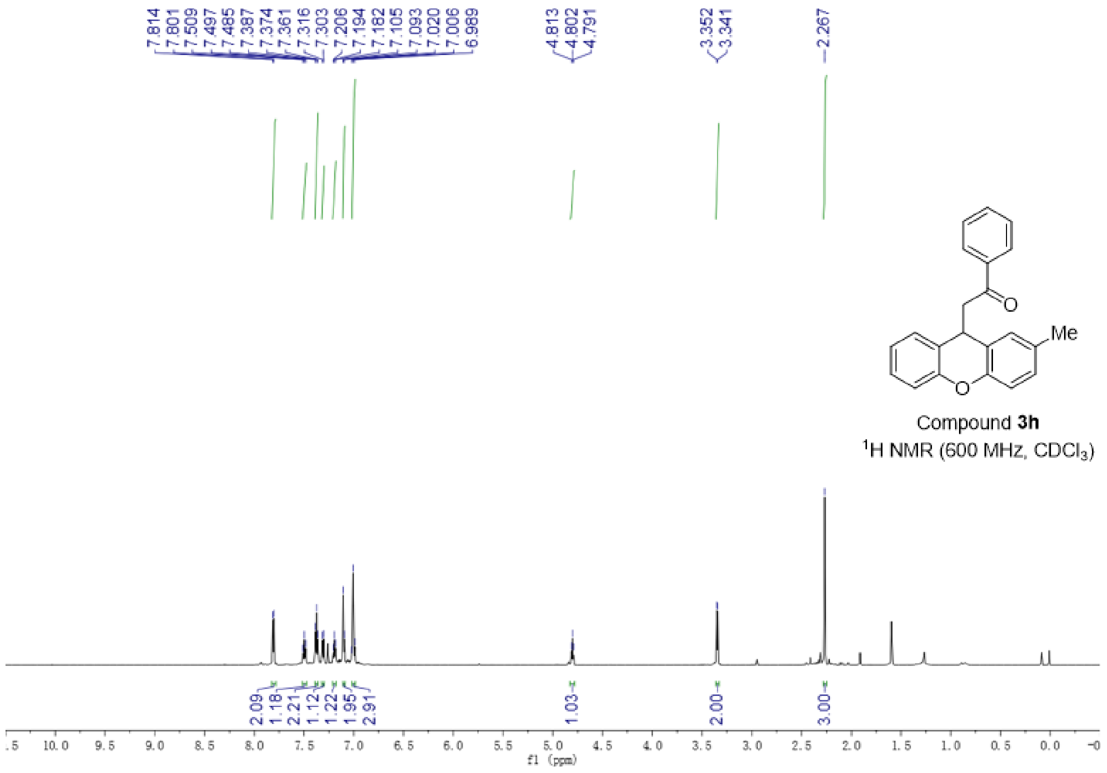

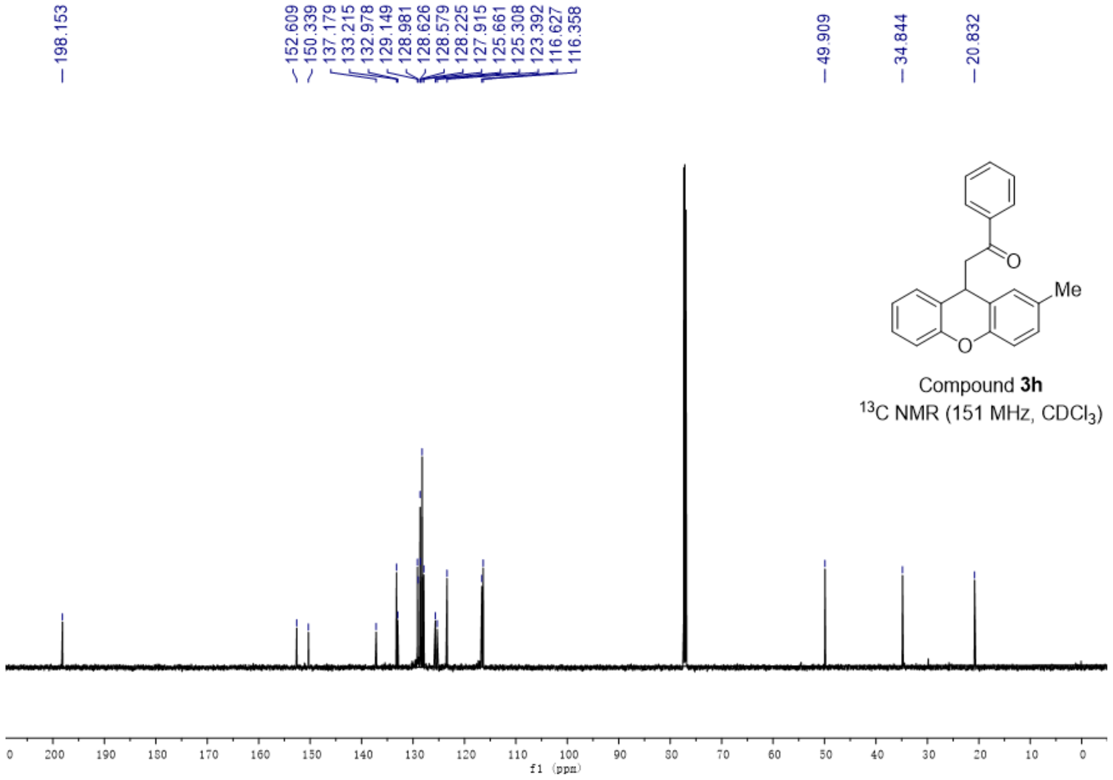

NMR spectra of 2-(4-methyl-9*H*-xanthen-9-yl)-1-phenylethan-1-one (**3i**)

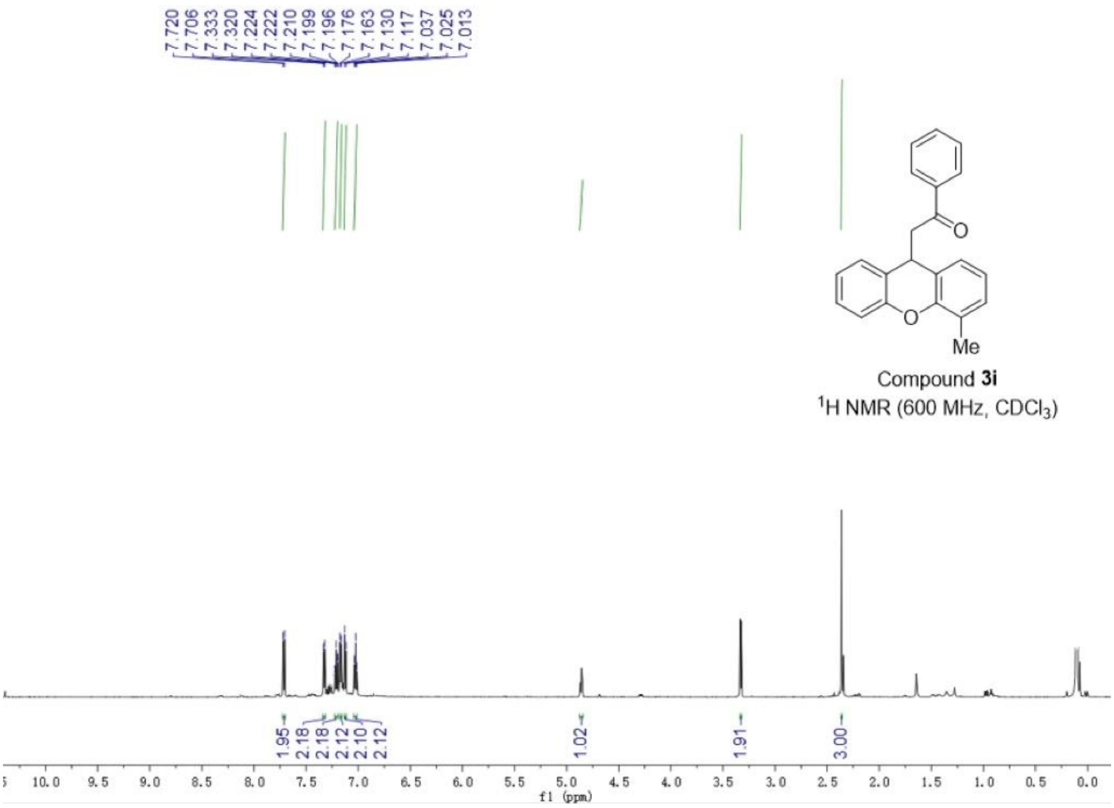

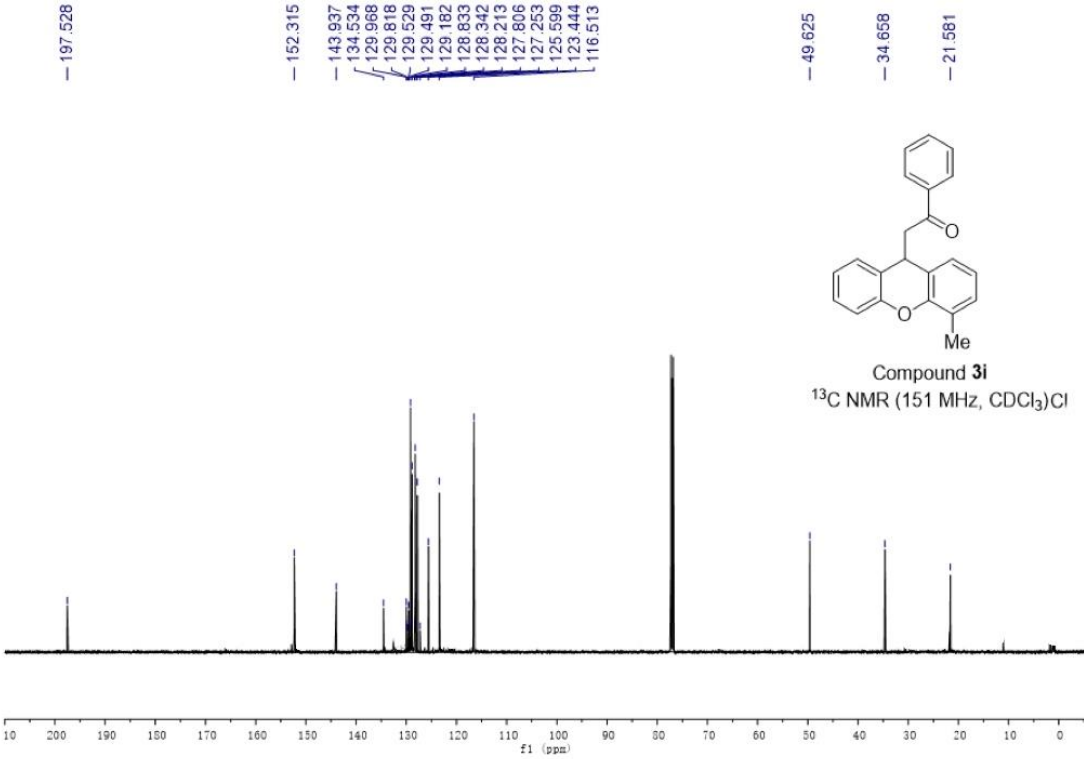

NMR spectra of 2-(4-(*tert*-butyl)-9*H*-xanthen-9-yl)-1-phenylethan-1-one (**3j**)

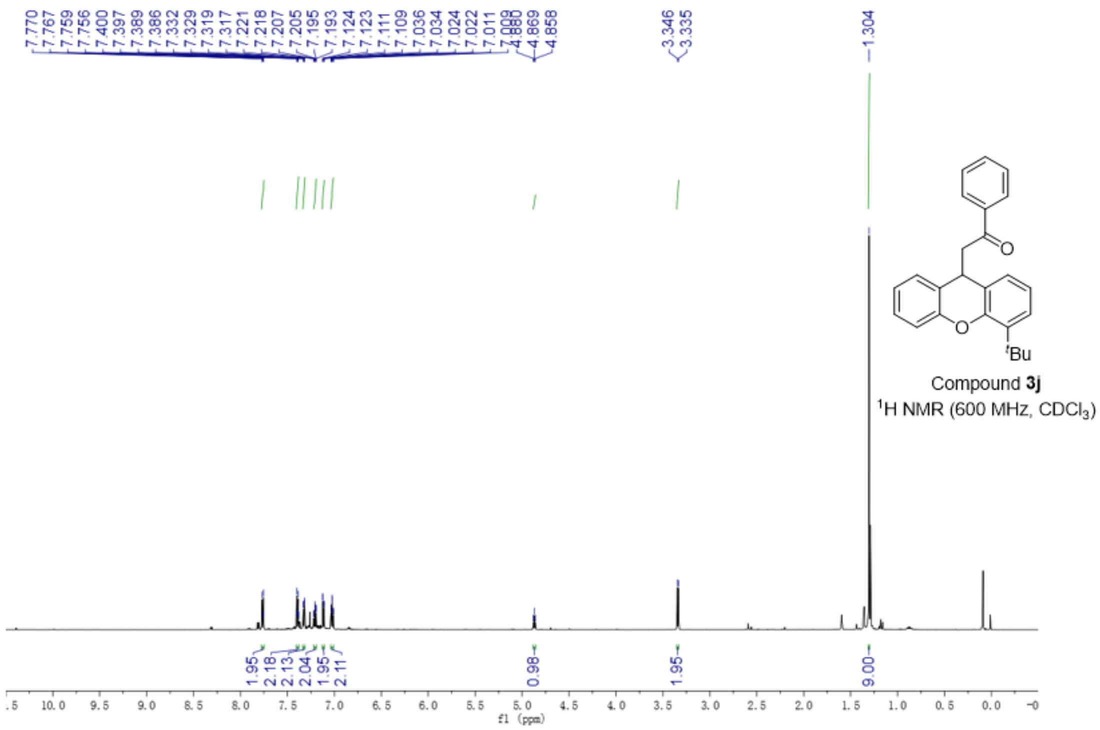

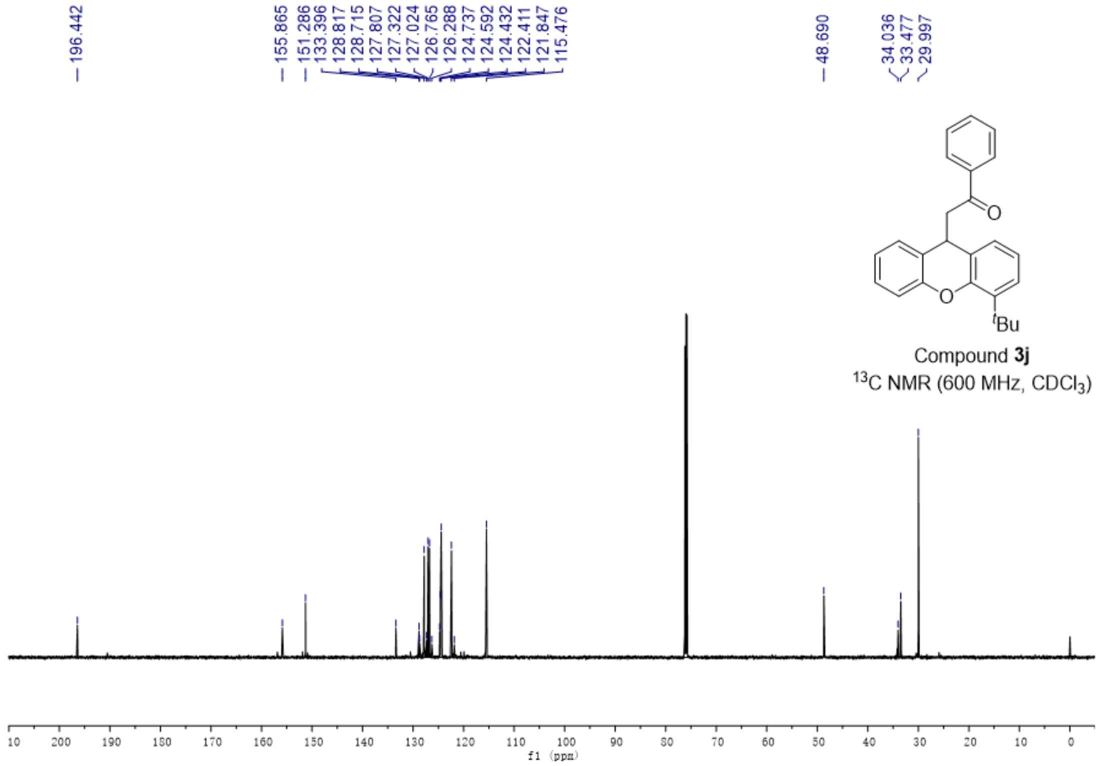

NMR spectra of *N*-(9*H*-xanthen-9-yl)acetamide (**4a**)

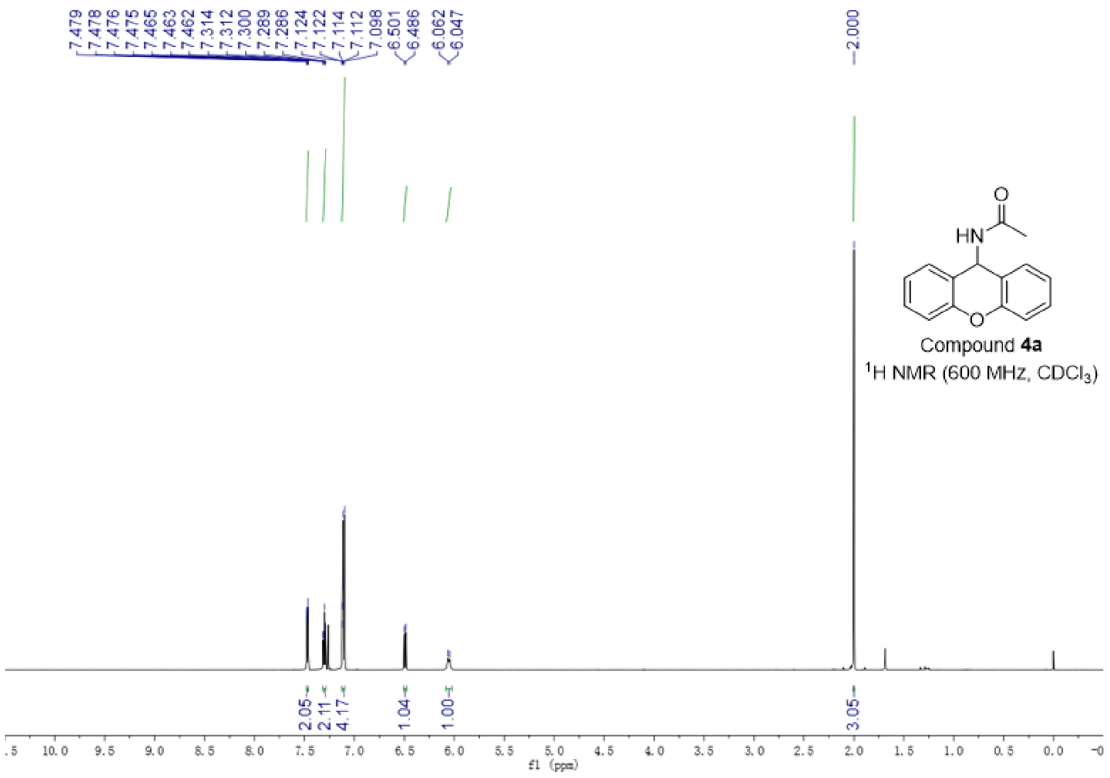

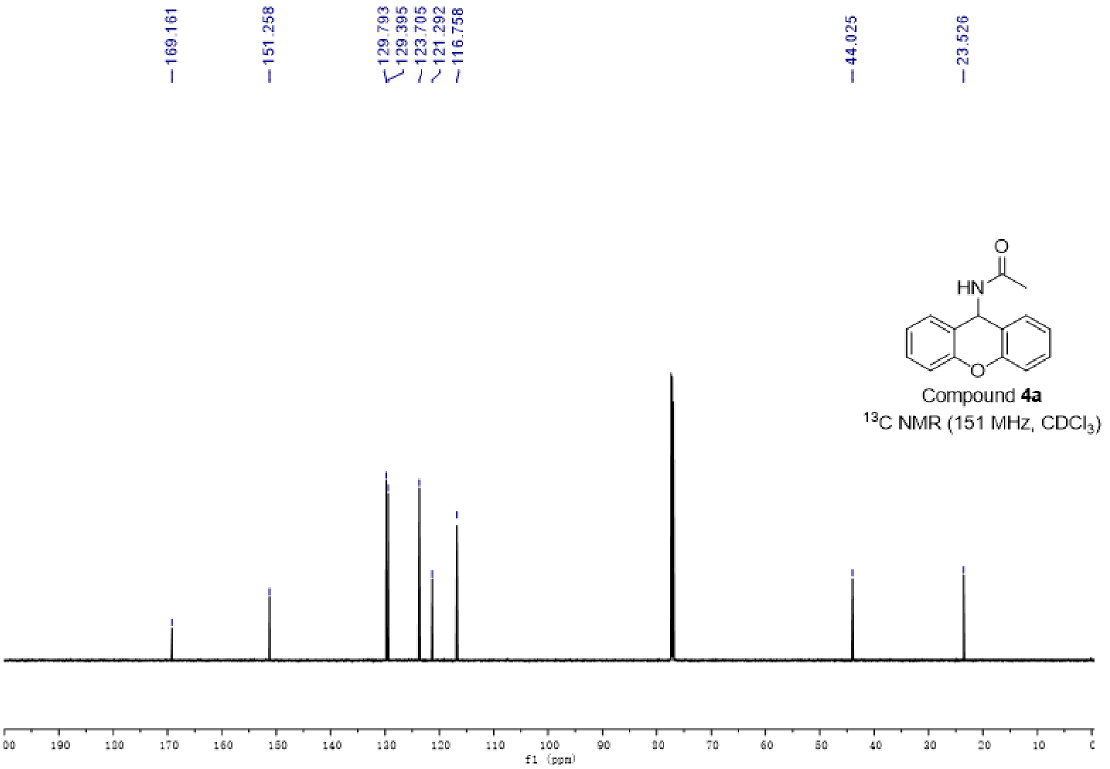

NMR spectra of *N*-(2-methyl-9*H*-xanthen-9-yl)acetamide (**4b**)

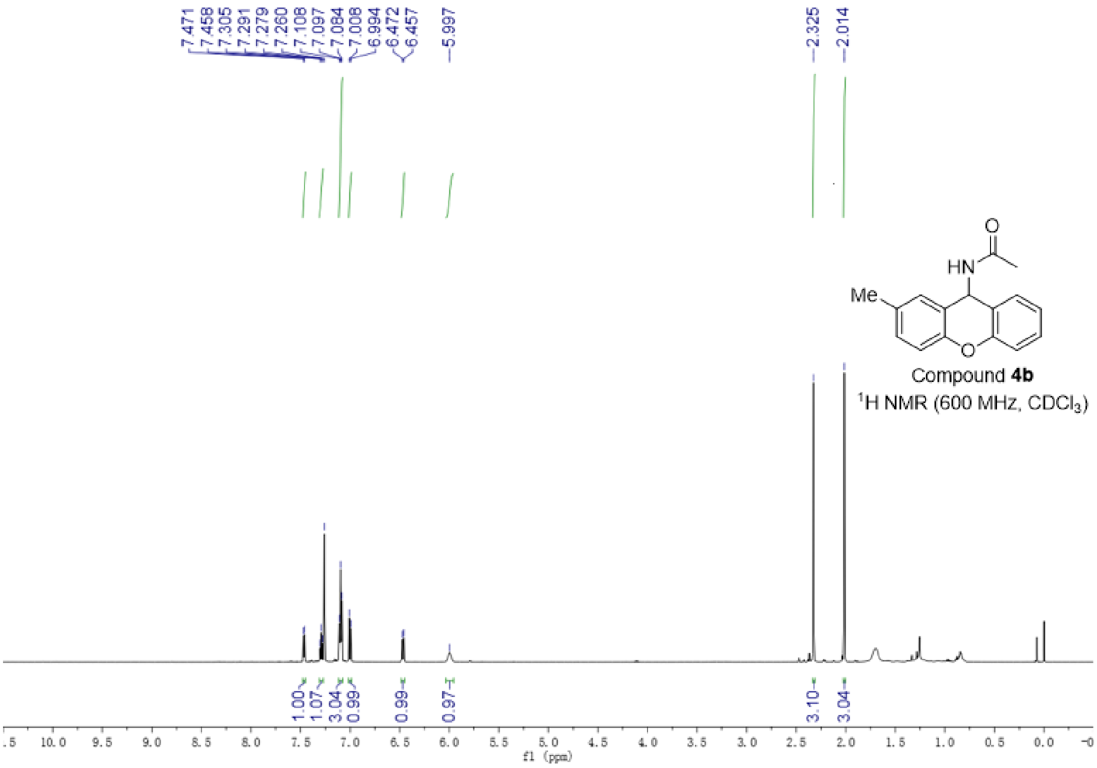

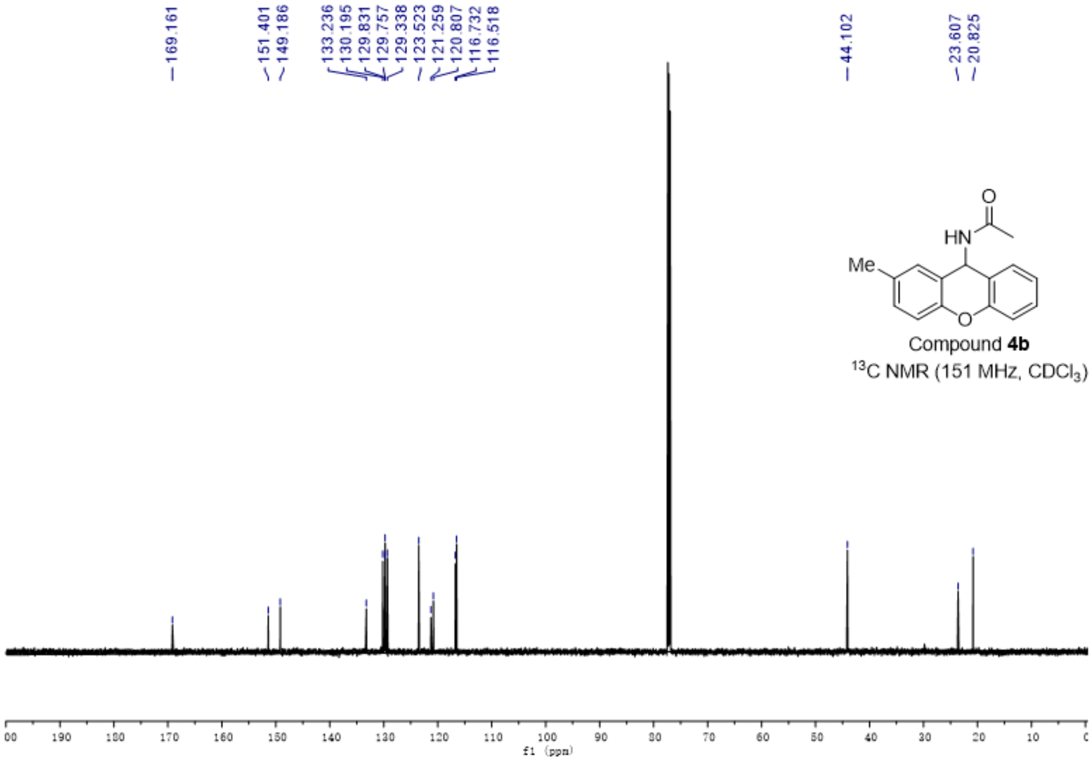

NMR spectra of *N*-(2-methoxy-9*H*-xanthen-9-yl)acetamide (**4c**)

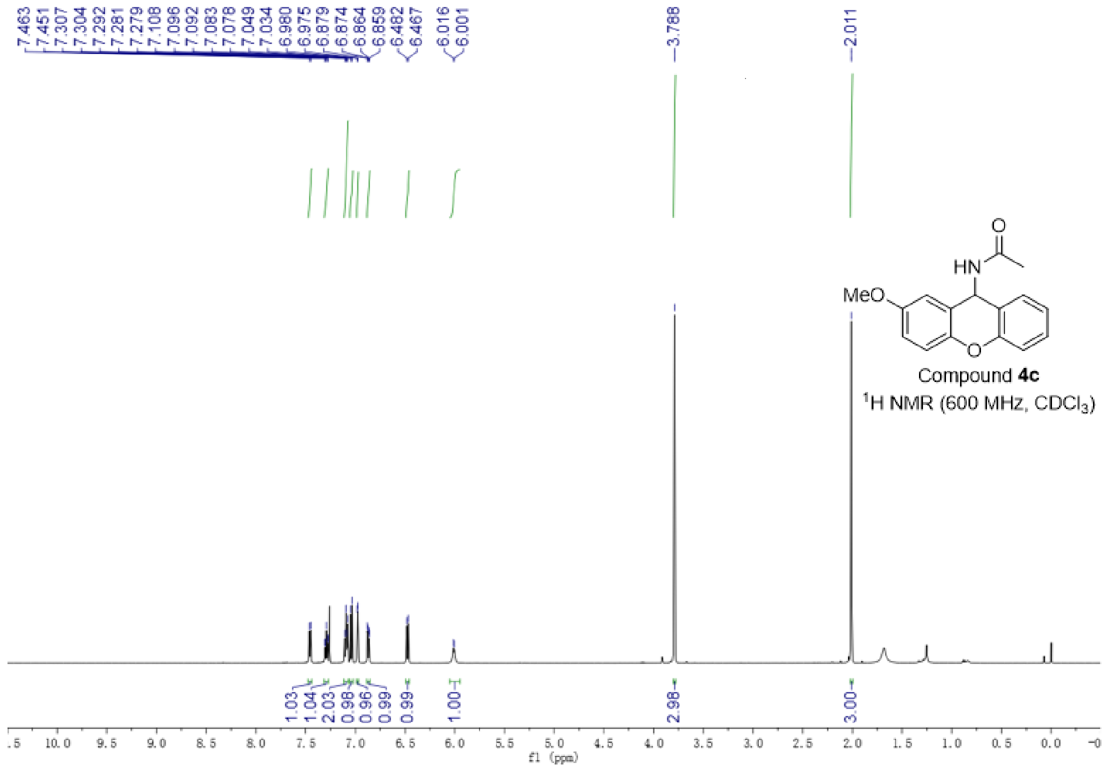

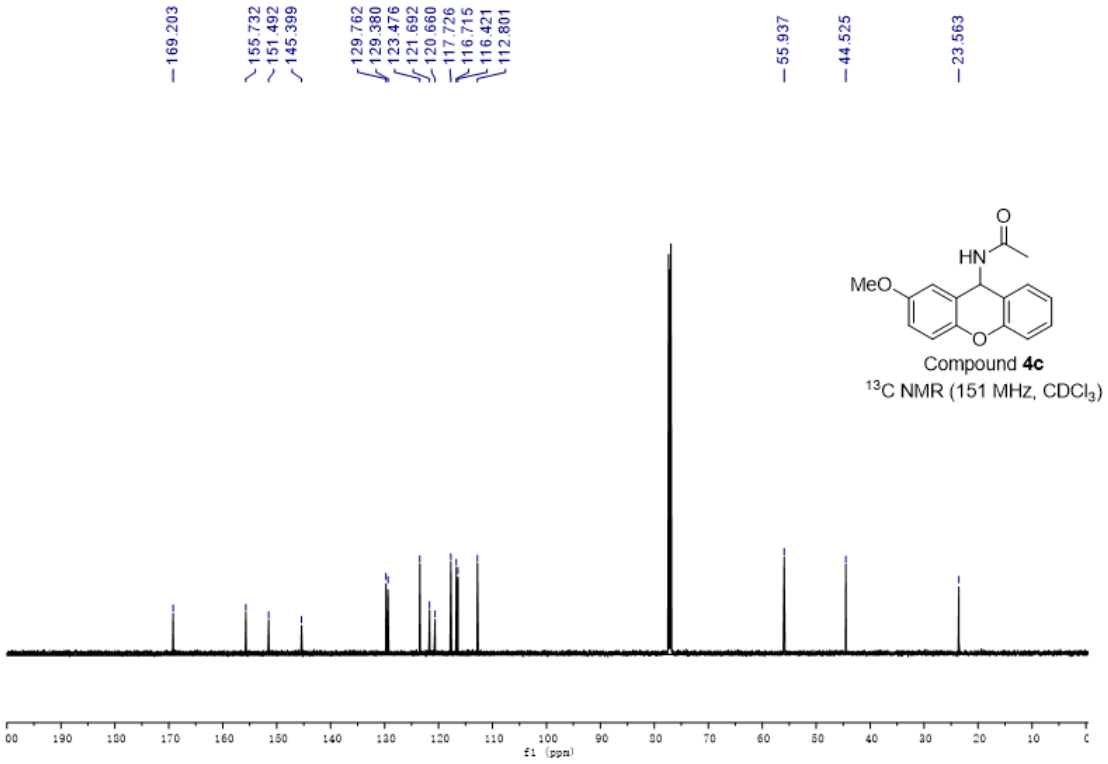

NMR spectra of *N*-(2-phenyl-9*H*-xanthen-9-yl)acetamide (**4d**)

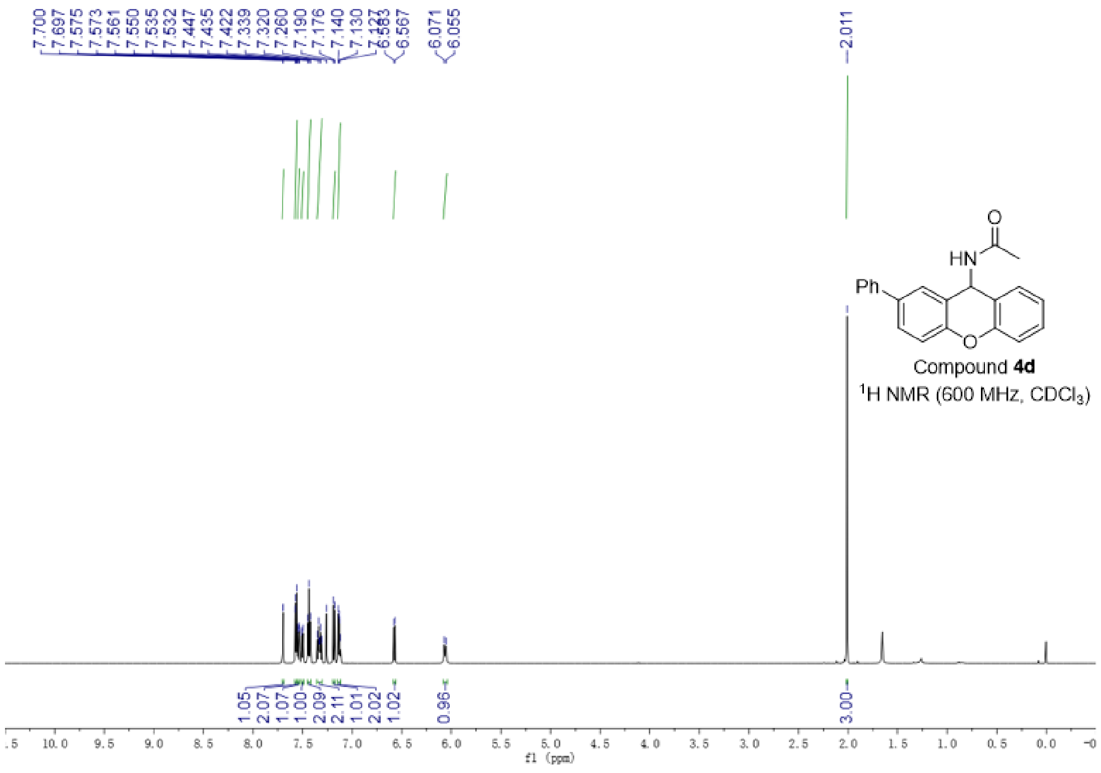

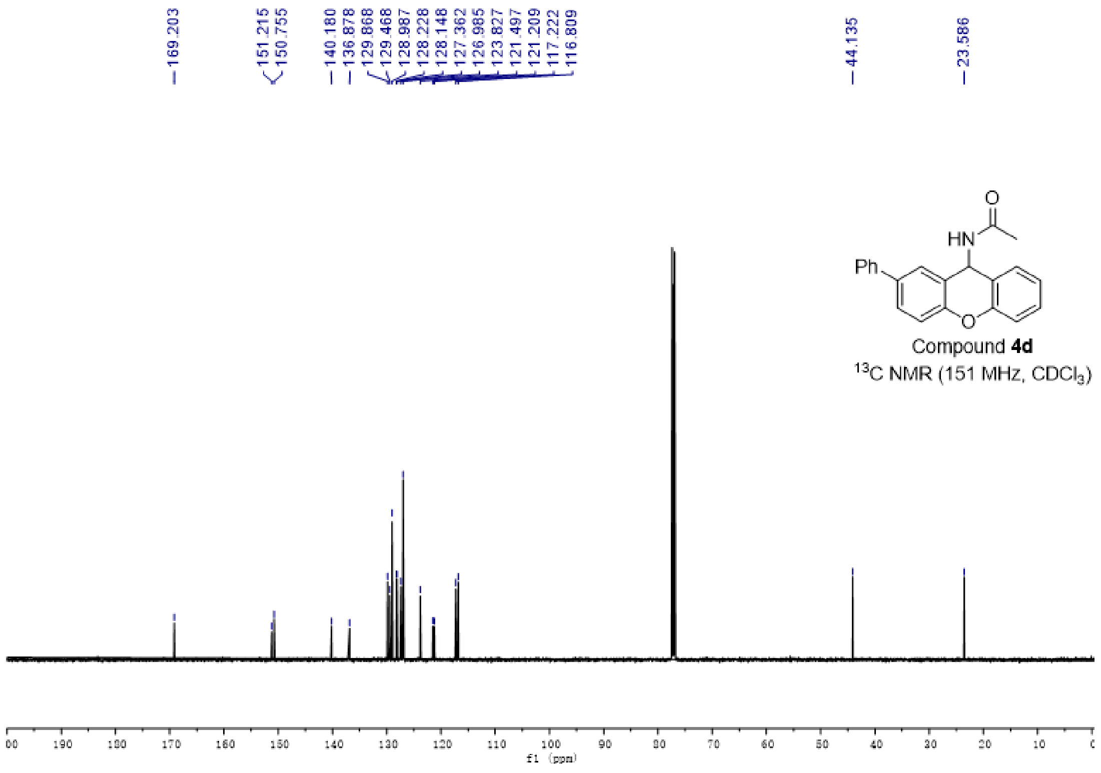

NMR spectra of *N*-(2-(trifluoromethyl)-9*H*-xanthen-9-yl)acetamide (**4e**)

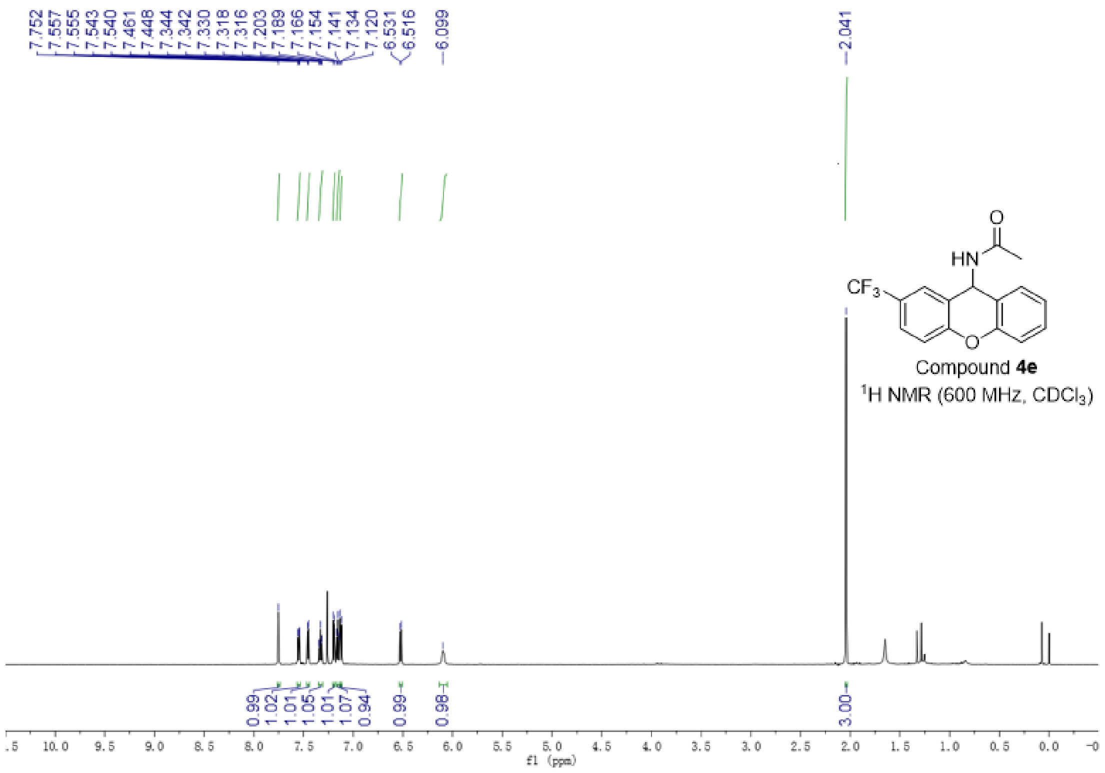

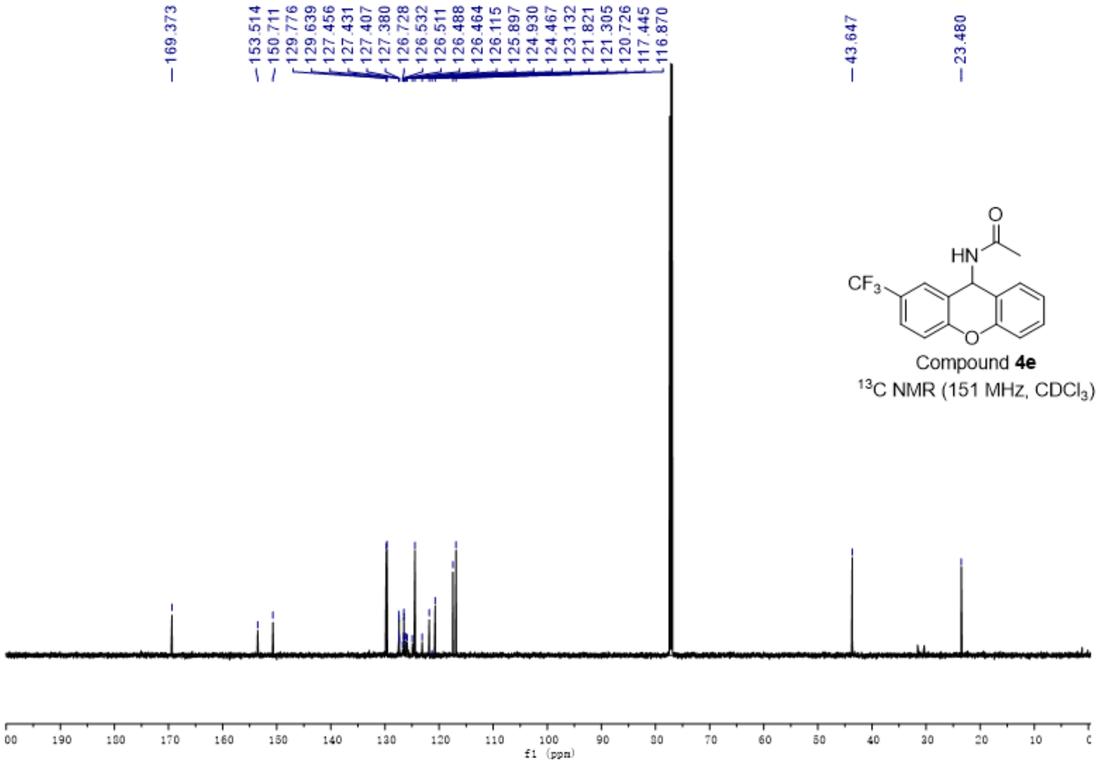

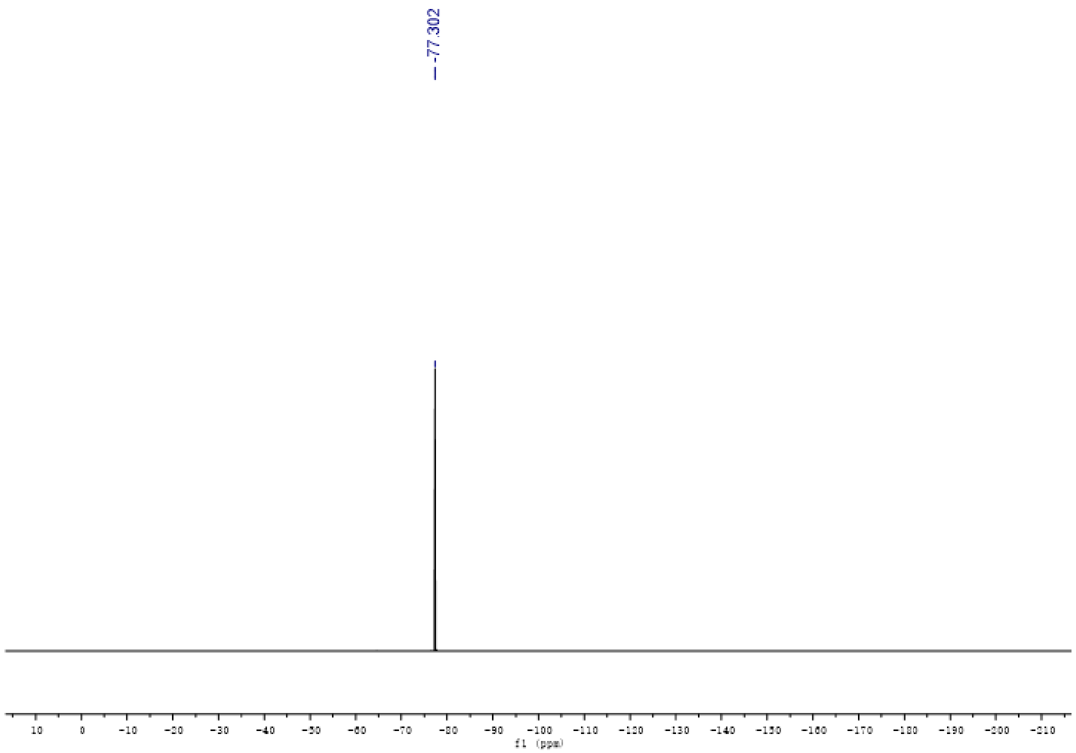

NMR spectra of 2-methyl-4-phenyl-5-(2-phenyl-9*H*-xanthen-9-yl)oxazole (**4f**)

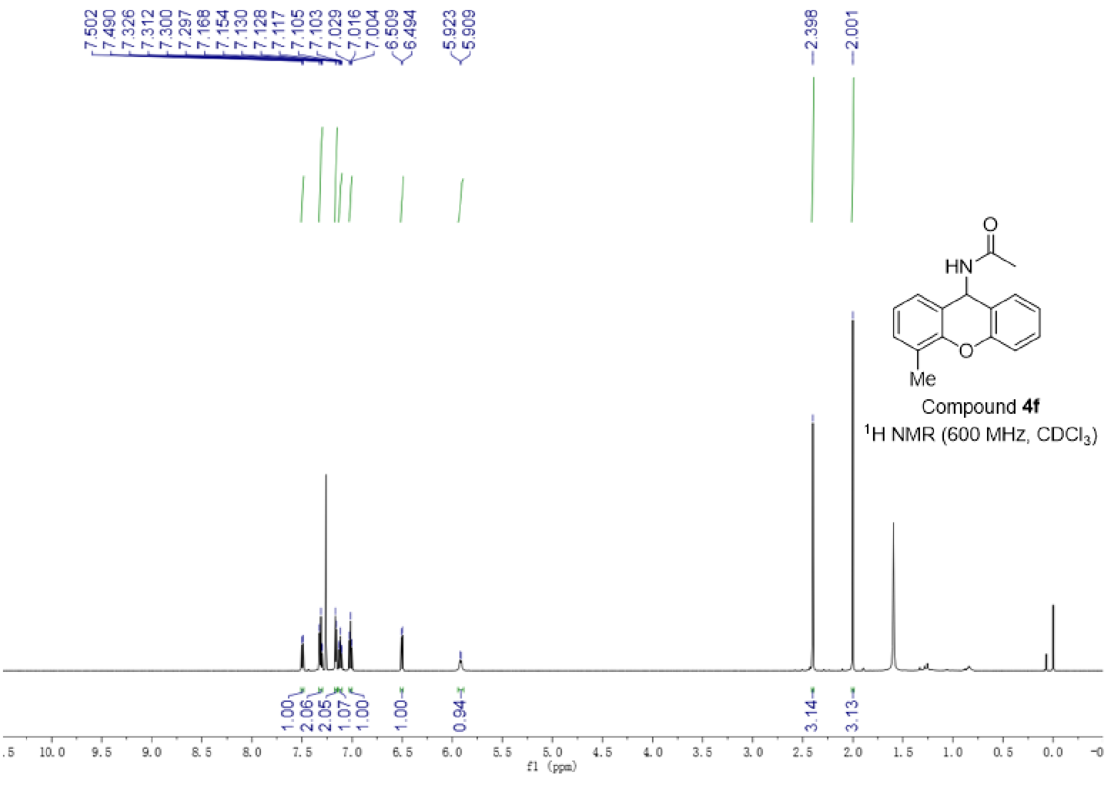

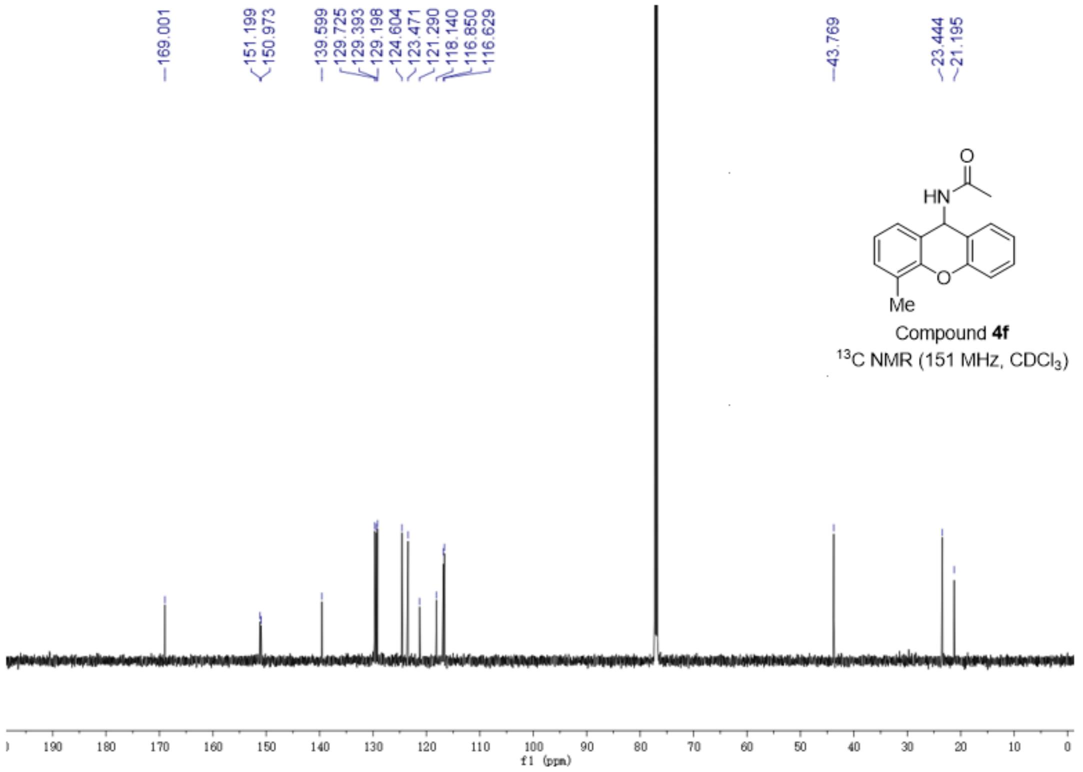

NMR spectra of *N*-(4-phenyl-9*H*-xanthen-9-yl)acetamide (**4g**)

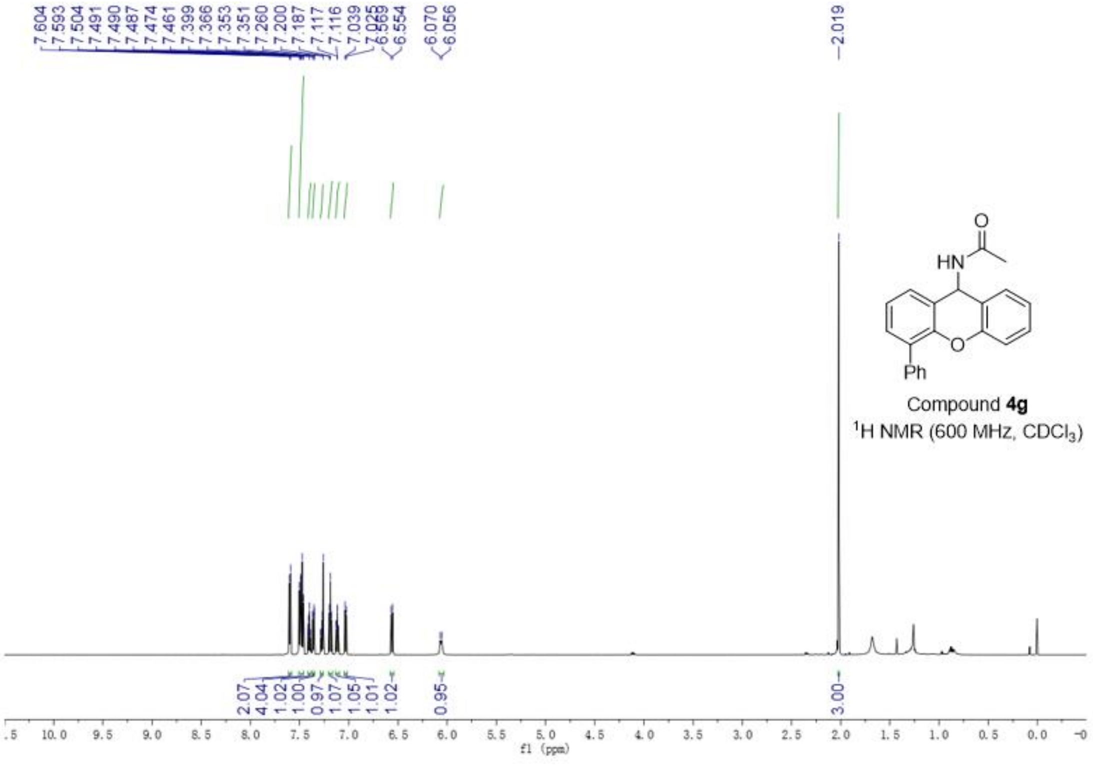

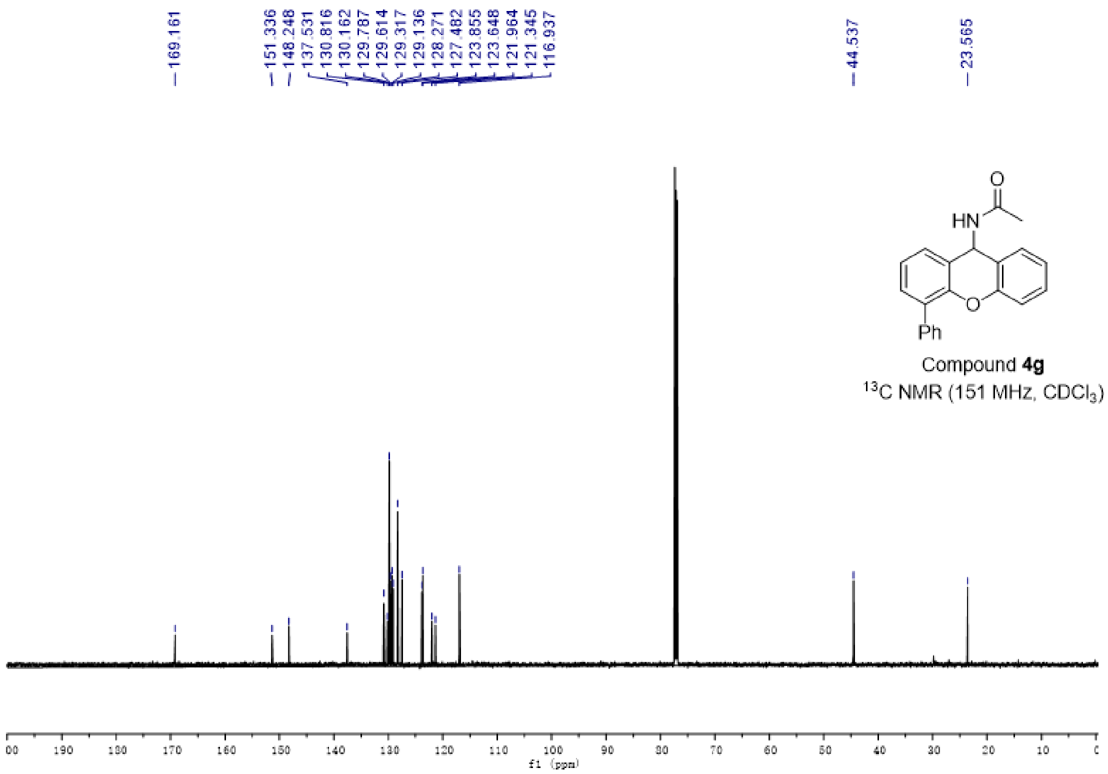

NMR spectra of *N*-(2,3-dimethyl-9,10-dihydroanthracen-9-yl)acetamide (**4h**)

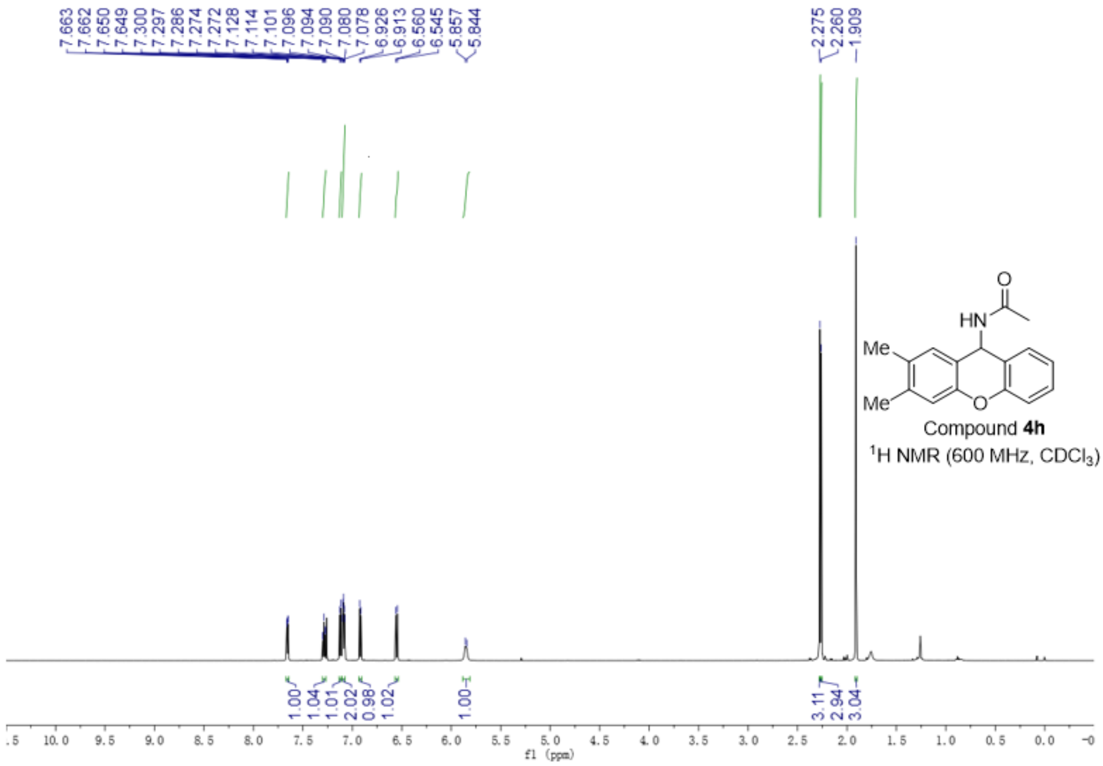

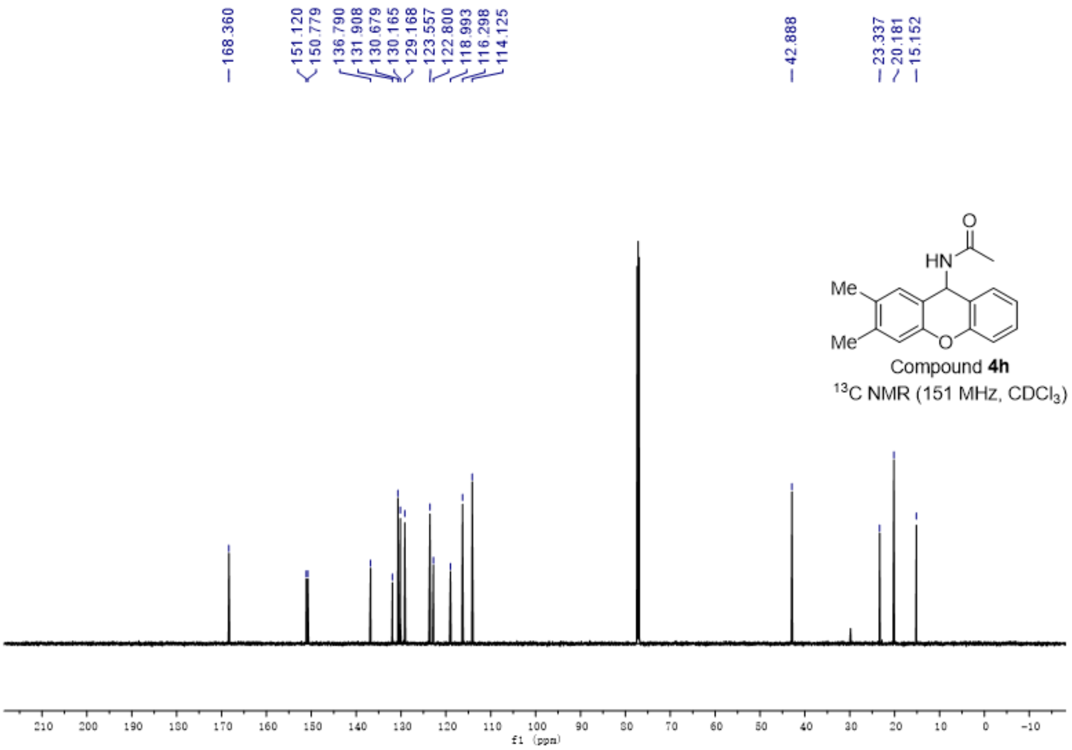

NMR spectra of *N*-(1,3-dimethyl-9,10-dihydroanthracen-9-yl)acetamide (**4i**)

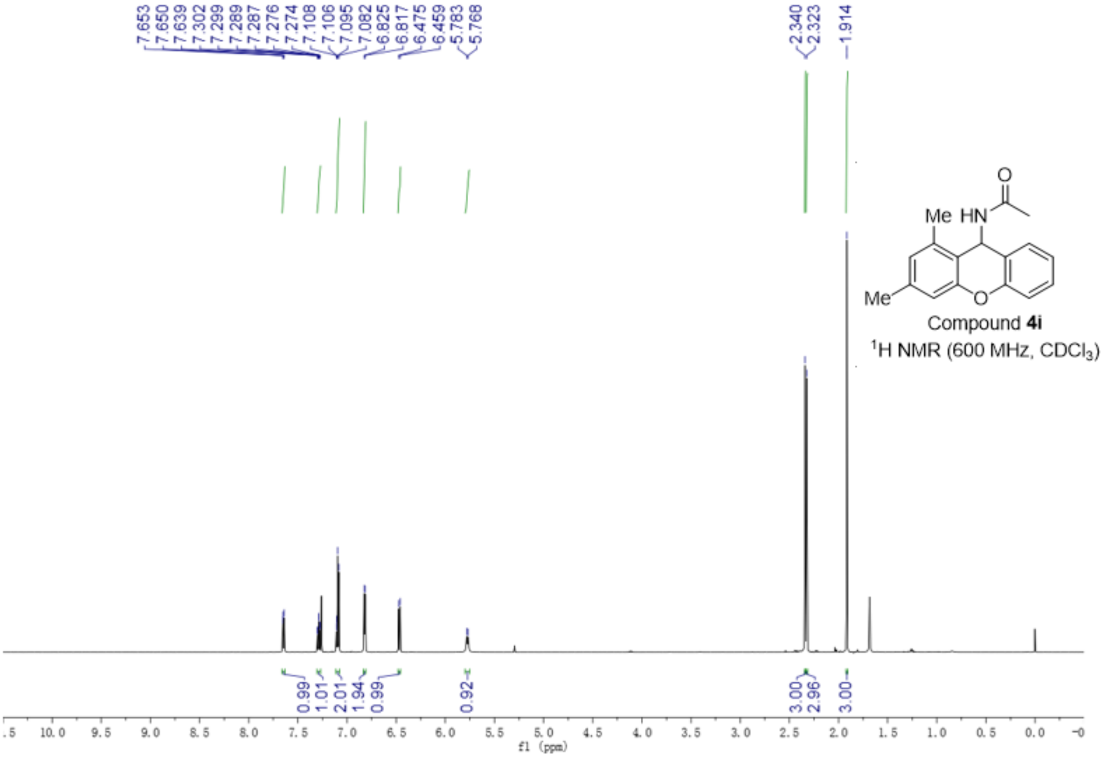

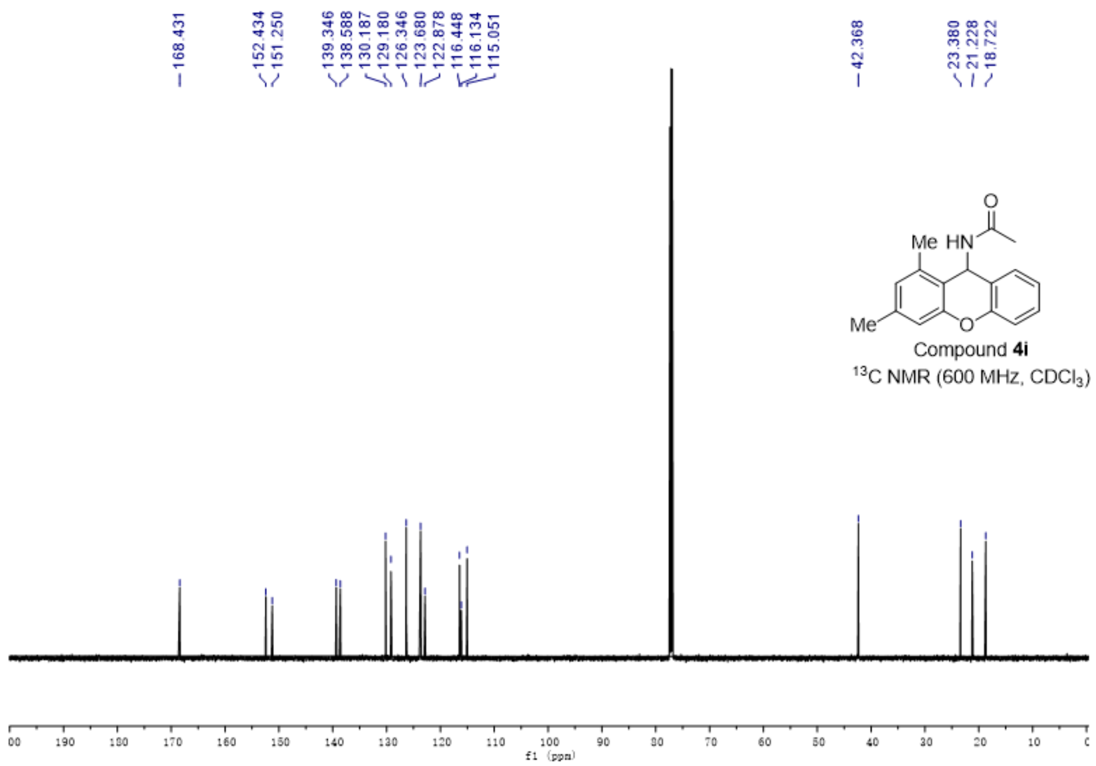

NMR spectra of *N*-(1,2,3,10-tetrahydrocyclopenta[*b*]xanthen-10-yl)acetamide **(4j)**

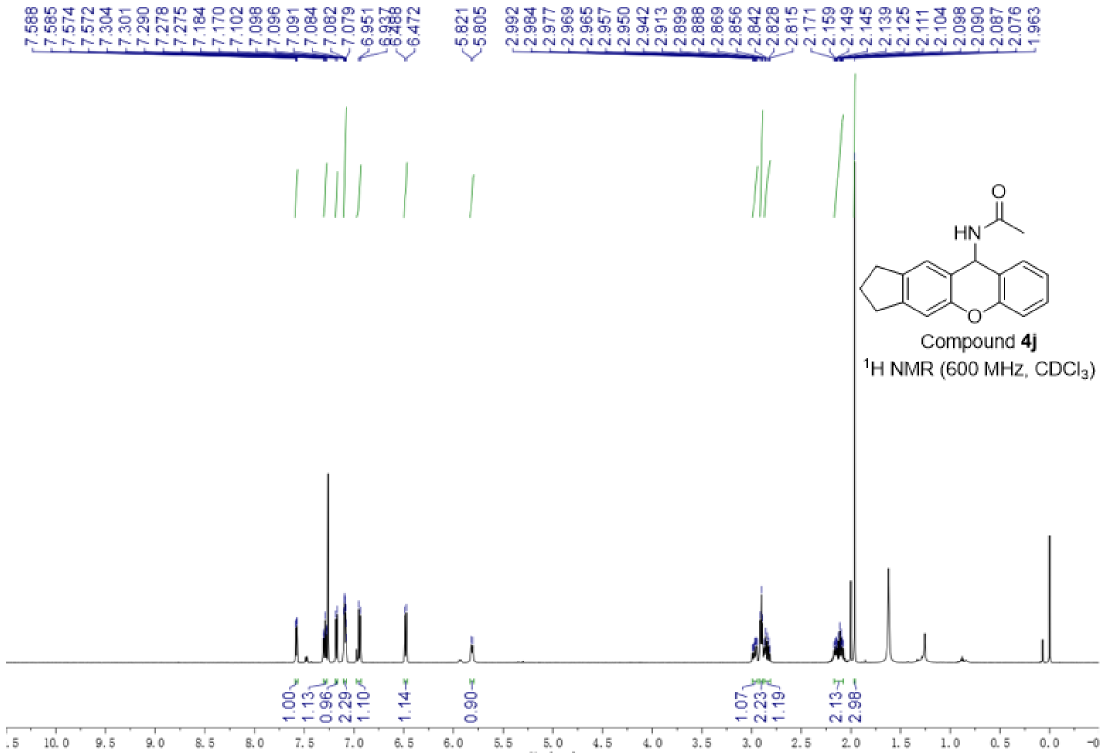

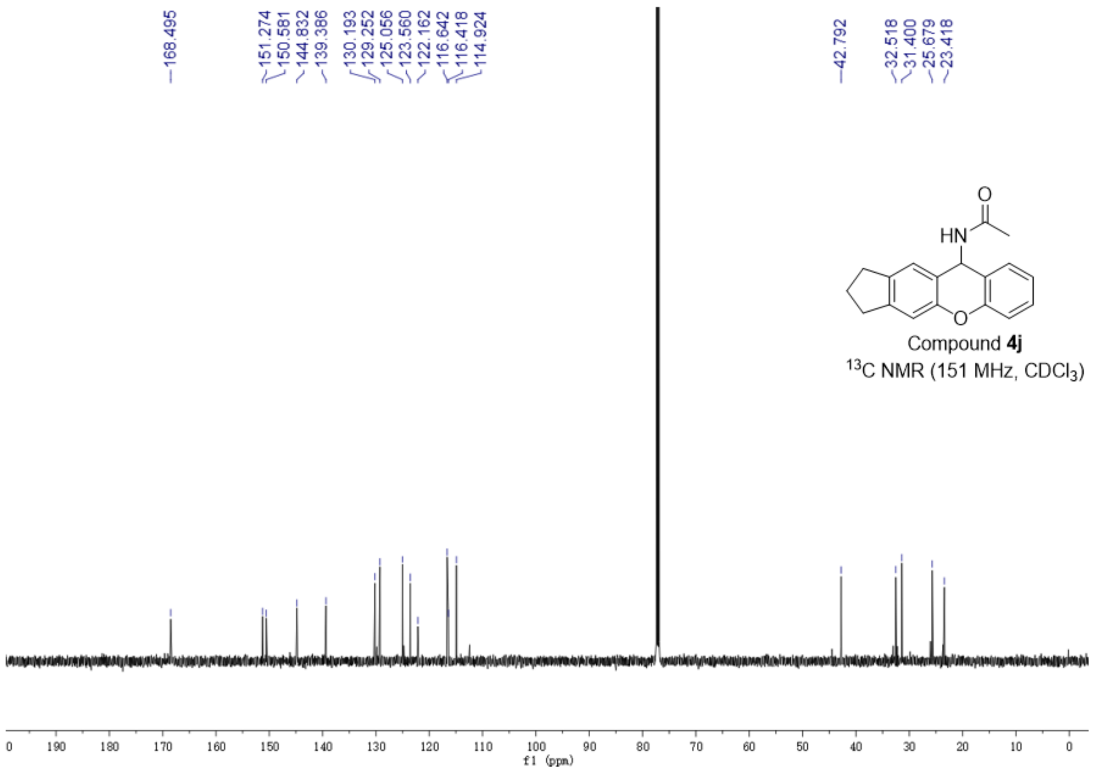

NMR spectra of *N*-(10-methoxy-12*H*-benzo[*a*]xanthen-12-yl)acetamide **(4k)**

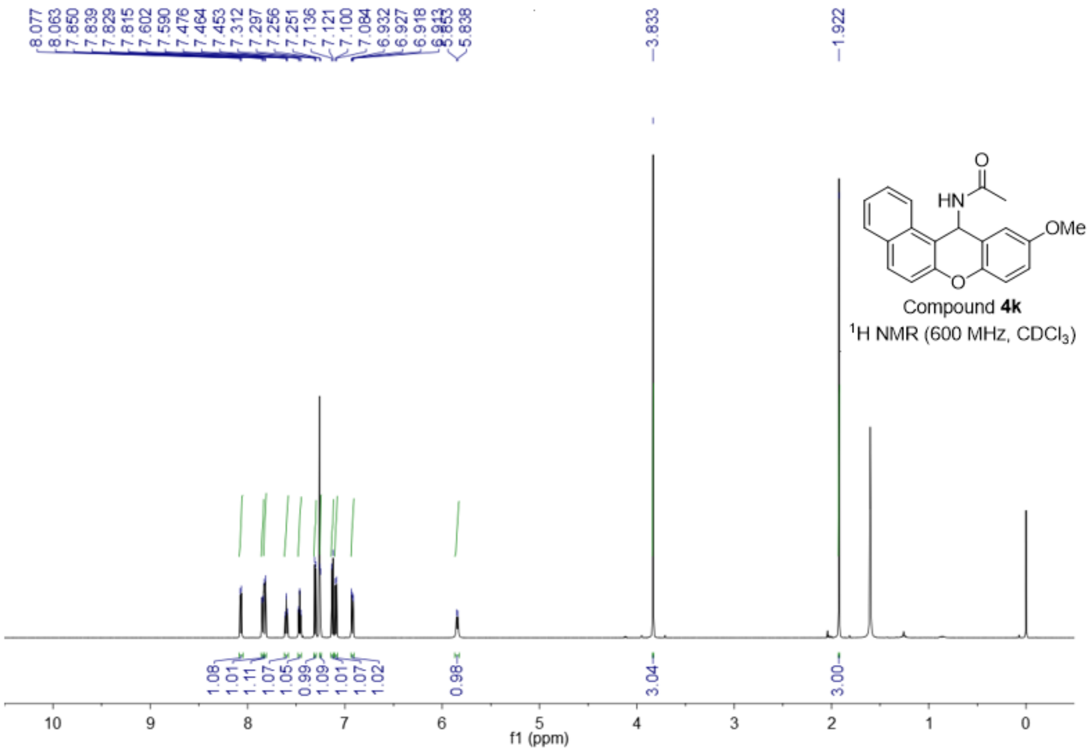

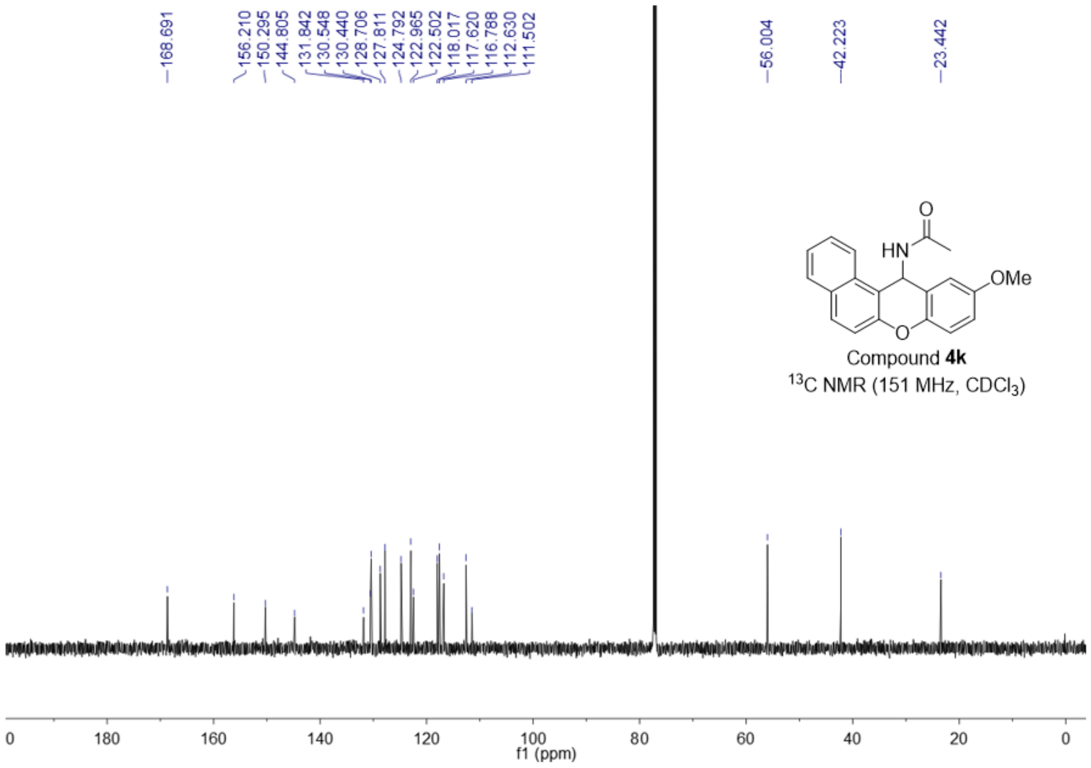

NMR spectra of *N*-(7*H*-benzo[*c*]xanthen-7-yl)acetamide **(4l)**

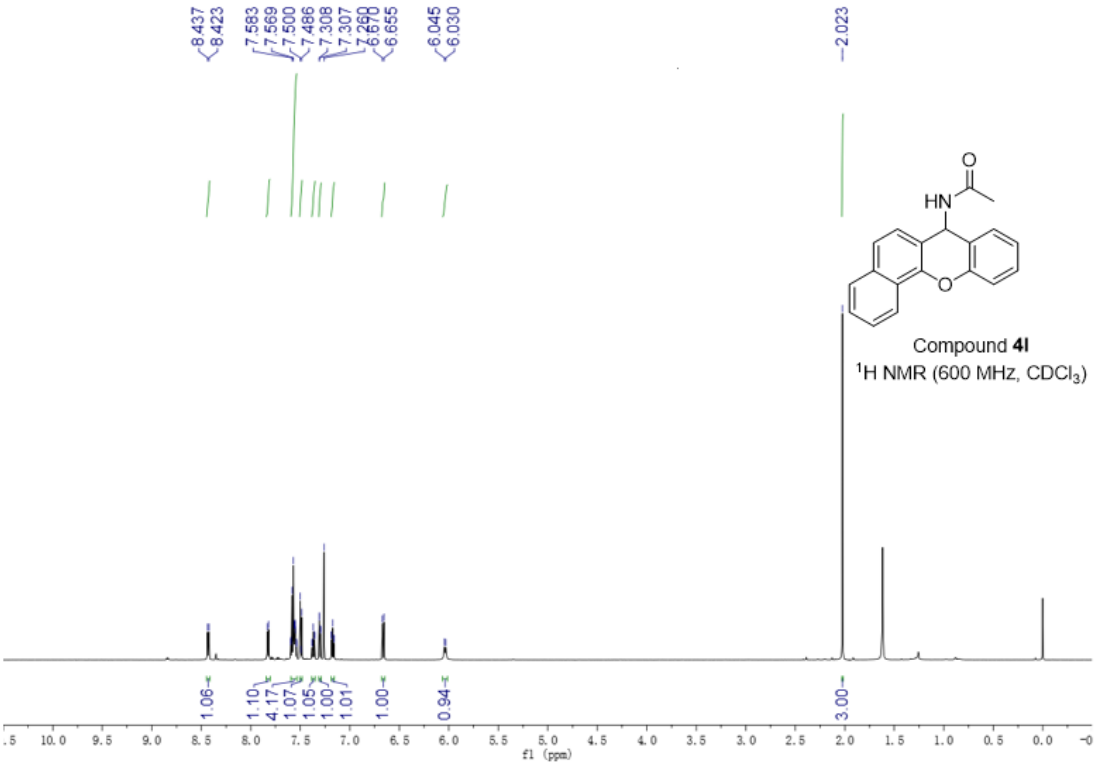

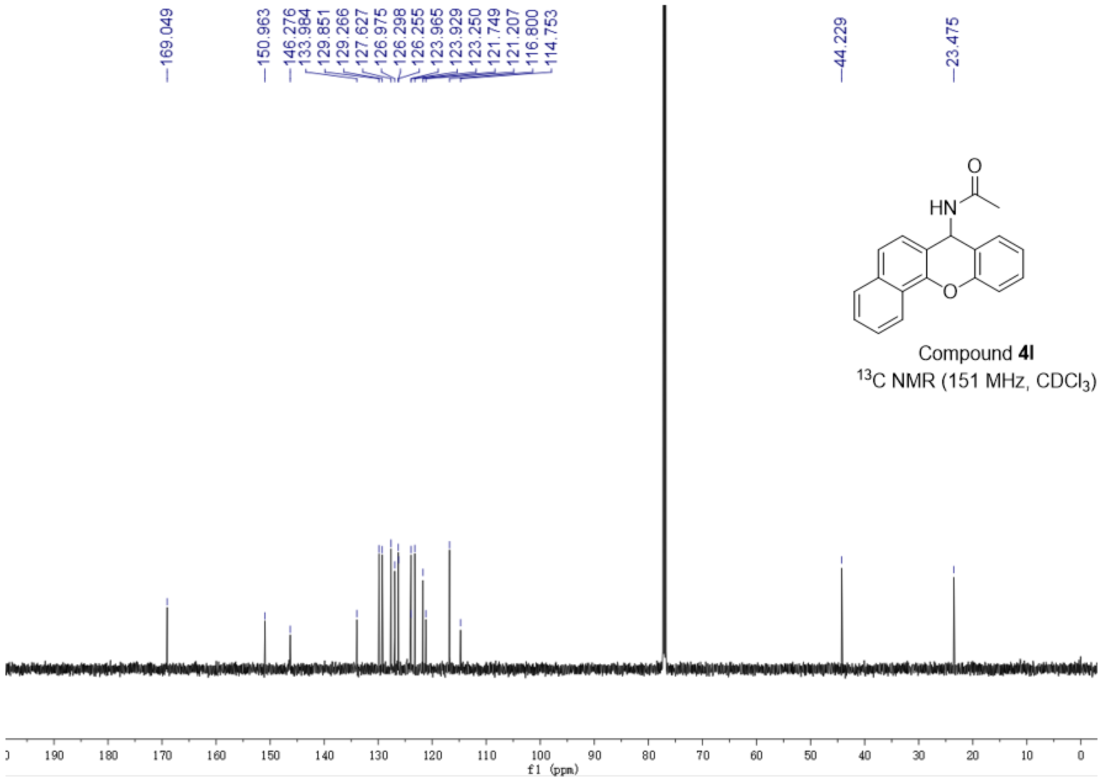

NMR spectra of *N*-(12*H*-benzo[*a*]xanthen-12-yl)acetamide (**4m**)

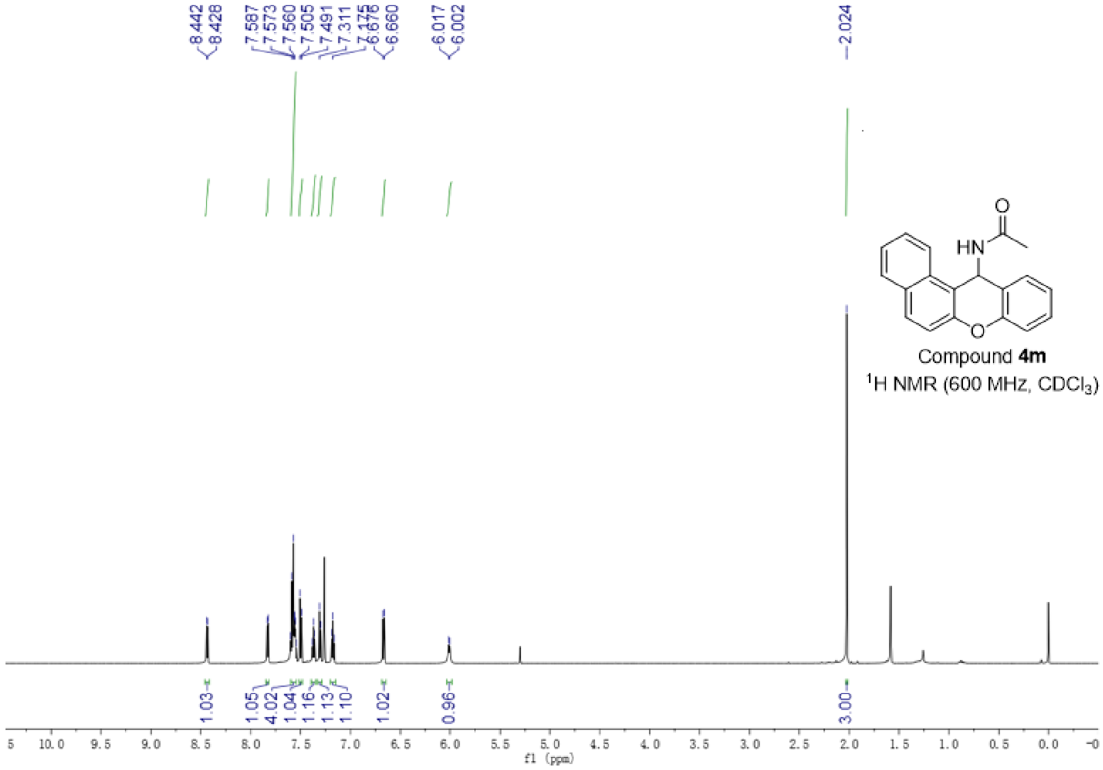

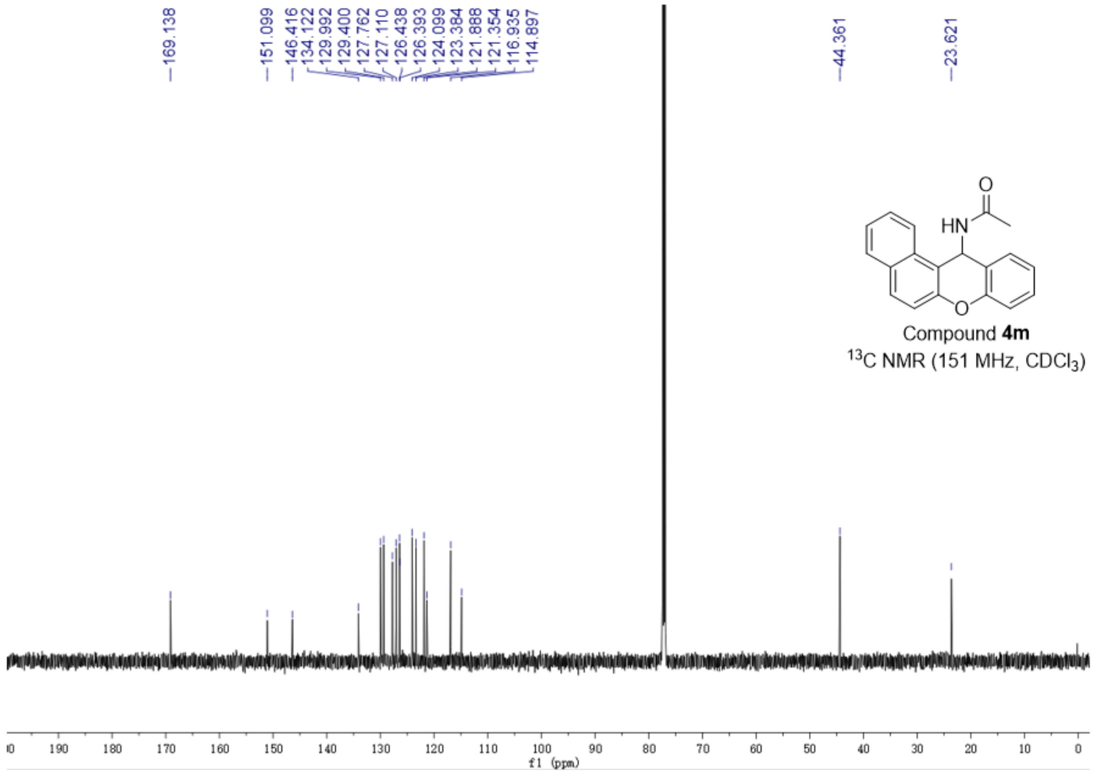

NMR spectra of *N*-(9*H*-xanthen-9-yl)butyramide **(4n)**

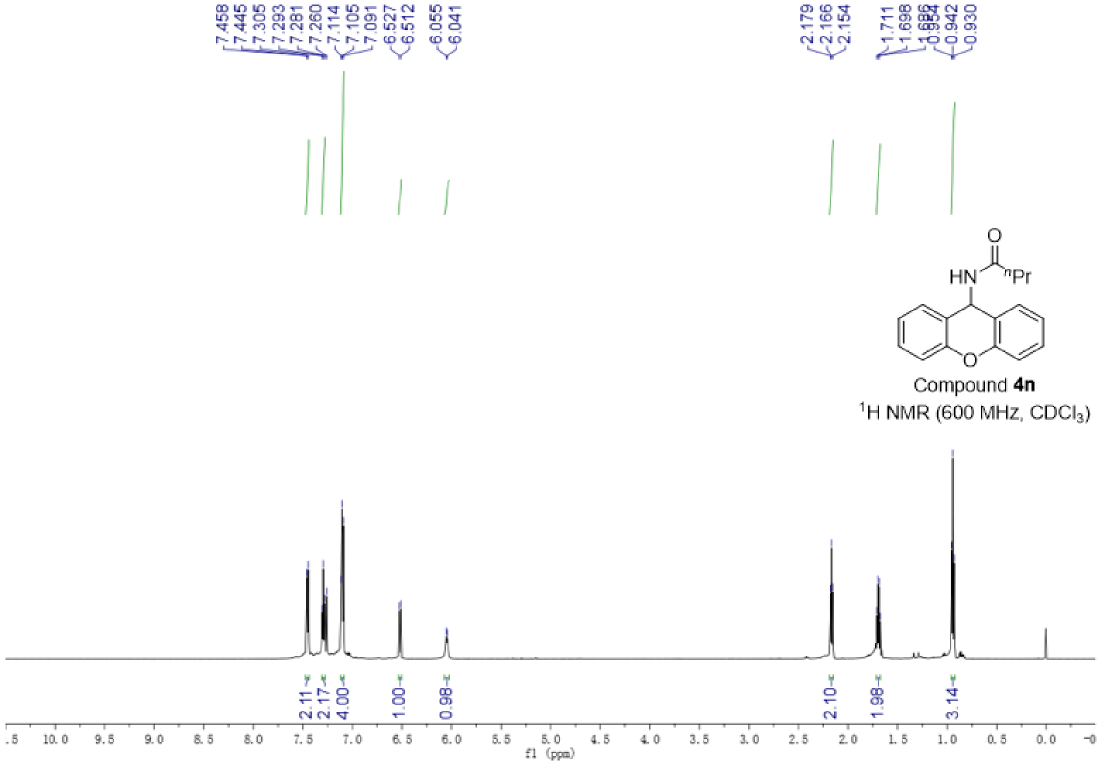

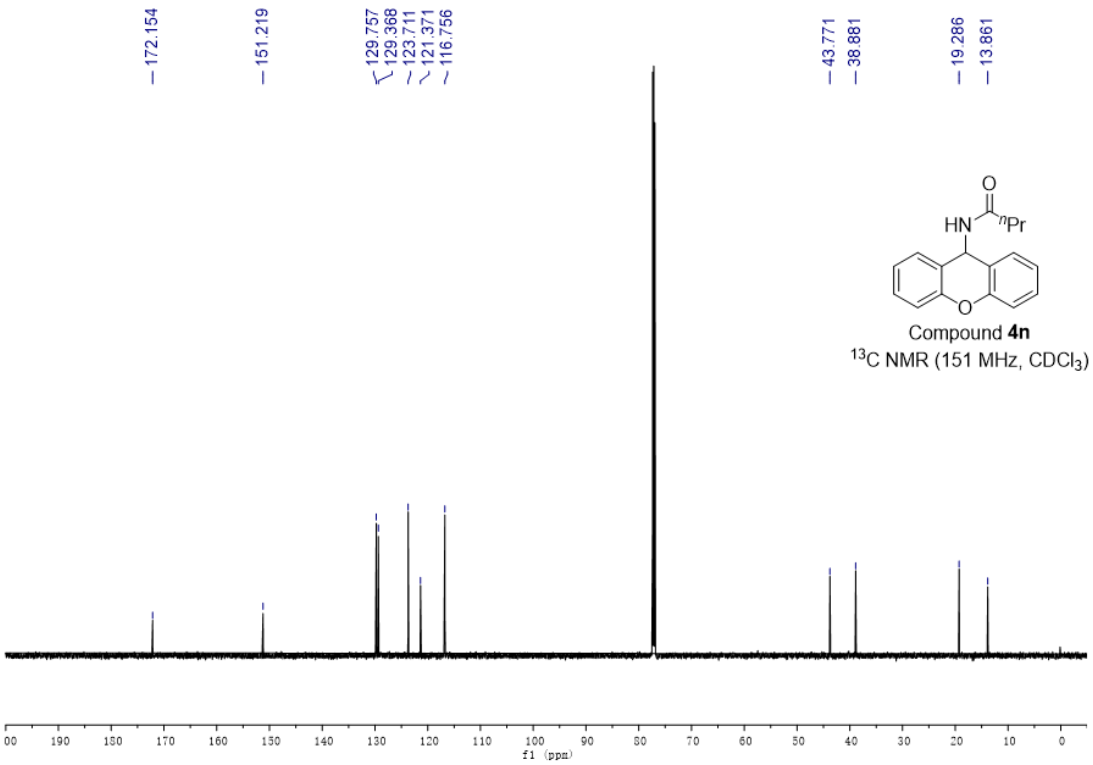

NMR spectra of *N*-(9*H*-thioxanthen-9-yl)acetamide **(4o)**

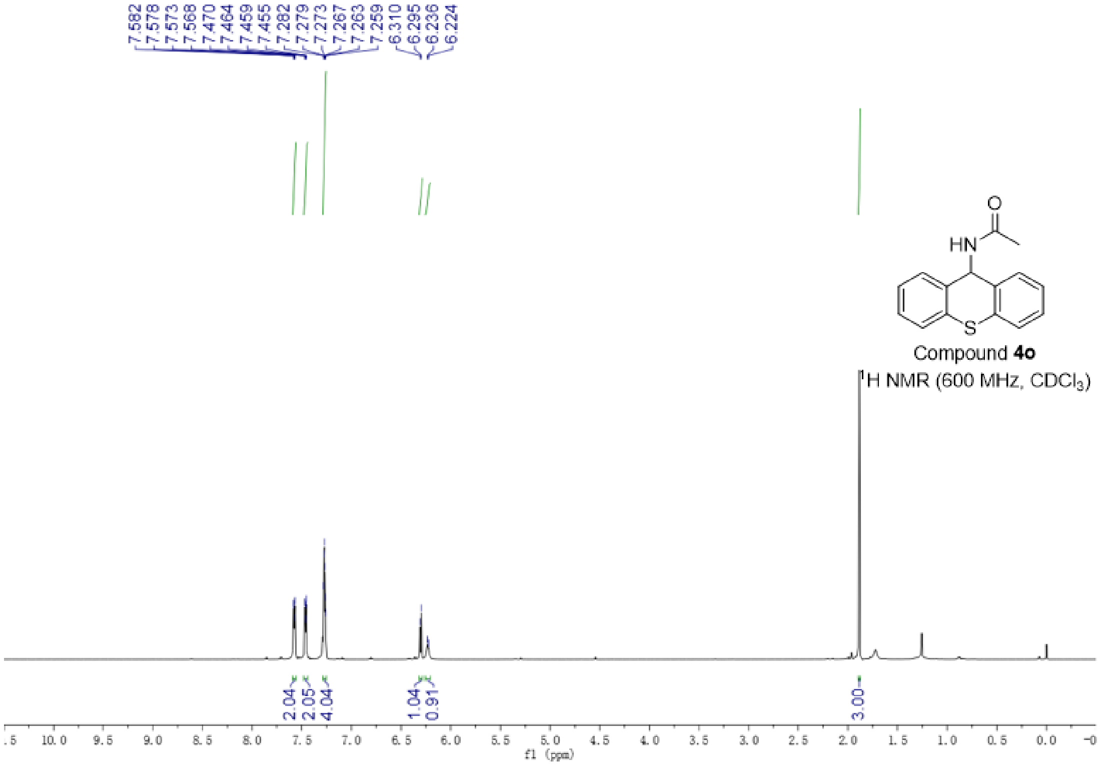

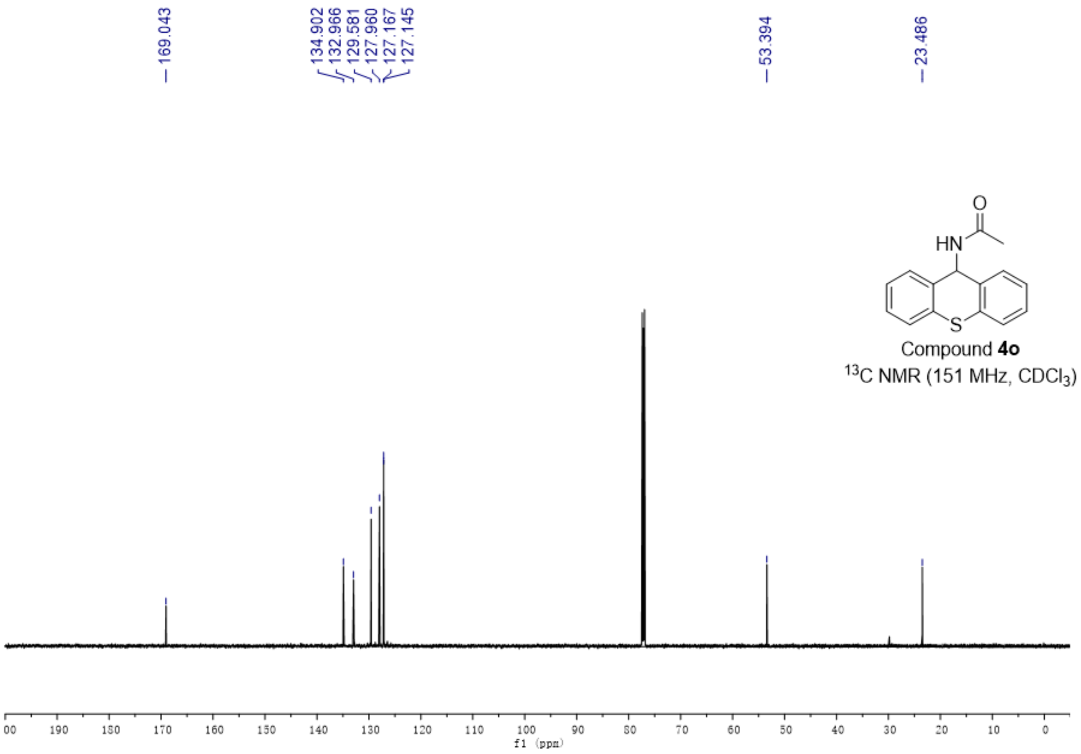

6. Determination of Faraday Efficiency

$$\mathrm{F.E.(\%)} = \frac{\text{n x F x mol of product or intermediate formed}}{\text{acculumated charge (C)}} \times 100\ \%$$

$$= \frac{2 \times 96485\ \mathrm{C\ mol^{-1}} \times 0.3\ \mathrm{mmol} \times 10^{-3} \times 68\ \%}{8\ \mathrm{mA} \times 10^{-3} \times 3\ \mathrm{h} \times 3600} \times 100\ \%$$

$$= 45.6\%$$

The F.E. (%) of the product **3a** was calculated by (1). The F.E. is the proportion of electrons consumed in each electrochemical reaction of the total applied charge and represents the selectivity of the electrochemical system for each reaction. In Eq (1), F is the Faradaic constant (96485 C mol^{-1}), and n is the number of electrons required for the production of products. The yield is the proportion of reactant converted to target product.

$$\text{F.E.}(\%) = \frac{\text{n x F x mol of product or intermediate formed}}{\text{acculumated charge (C)}} \text{ x } 100\ \%$$

$$= \frac{2 \text{ x } 96485 \text{ C mol}^{-1} \text{ x } 0.3 \text{ mmol} \times 10^{-3} \text{ x } 71\ \%}{8 \text{ mA x } 10^{-3} \text{ x } 5 \text{ h x } 3600} \text{ x } 100\ \%$$

$$= 28.5\%$$

The F.E. (%) of the product **4a** was calculated by (1). The F.E. is the proportion of electrons consumed in each electrochemical reaction of the total applied charge and represents the selectivity of the electrochemical system for each reaction. In Eq (1), F is the Faradaic constant (96485 C mol^{-1}), and n is the number of electrons required for the production of products. The yield is the proportion of reactant converted to target product.
